# Molecular Recognition of SARS-CoV-2 Mpro Inhibitors: Insights from Cheminformatics and Quantum Chemistry

**DOI:** 10.3390/molecules30102174

**Published:** 2025-05-15

**Authors:** Adedapo Olosunde, Xiche Hu

**Affiliations:** Department of Chemistry and Biochemistry, University of Toledo, Toledo, OH 43606, USA; adedapo.olosunde@rockets.utoledo.edu

**Keywords:** SARS-CoV-2 main protease (Mpro), covalent and non-covalent inhibitors, quantum mechanics, molecular recognition, π–π stacking interactions, CH–π interactions, hydrogen bonding, rational drug design

## Abstract

The SARS-CoV-2 main protease (Mpro), essential for viral replication, remains a prime target for antiviral drug design against COVID-19 and related coronaviruses. In this study, we present a systematic investigation into the molecular determinants of Mpro inhibition using an integrated approach combining large-scale data mining, cheminformatics, and quantum chemical calculations. A curated dataset comprising 963 high-resolution structures of Mpro–ligand complexes—348 covalent and 615 non-covalent inhibitors—was mined from the Protein Data Bank. Cheminformatics analysis revealed distinct physicochemical profiles for each inhibitor class: covalent inhibitors tend to exhibit higher hydrogen bonding capacity and sp^3^ character, while non-covalent inhibitors are enriched in aromatic rings and exhibit greater aromaticity and lipophilicity. A novel descriptor, Weighted Hydrogen Bond Count (WHBC), normalized for molecular size, revealed a notable inverse correlation with aromatic ring count, suggesting a compensatory relationship between hydrogen bonding and π-mediated interactions. To elucidate the energetic underpinnings of molecular recognition, 40 representative inhibitors (20 covalent, 20 non-covalent) were selected based on principal component analysis and aromatic ring content. Quantum mechanical calculations at the double-hybrid B2PLYP/def2-QZVP level quantified non-bonded interaction energies, revealing that covalent inhibitors derive binding strength primarily through hydrogen bonding (~63.8%), whereas non-covalent inhibitors depend predominantly on π–π stacking and CH–π interactions (~62.8%). Representative binding pocket analyses further substantiate these findings: the covalent inhibitor F2F-2020198-00X exhibited strong hydrogen bonds with residues such as Glu166 and His163, while the non-covalent inhibitor EDG-MED-10fcb19e-1 engaged in extensive π-mediated interactions with residues like His41, Met49, and Met165. The distinct interaction patterns led to the establishment of pharmacophore models, highlighting key recognition motifs for both covalent and non-covalent inhibitors. Our findings underscore the critical role of aromaticity and non-bonded π interactions in driving binding affinity, complementing or, in some cases, substituting for hydrogen bonding, and offer a robust framework for the rational design of next-generation Mpro inhibitors with improved selectivity and resistance profiles.

## 1. Introduction

The coronavirus disease 2019 (COVID-19) pandemic, caused by the severe acute respiratory syndrome coronavirus 2 (SARS-CoV-2), has had a profound global health, economic, and social impact, with millions of illnesses and deaths reported worldwide. The emergence of this new coronavirus underscored the critical need for efficient drug discovery strategies that target key viral proteins [1]. SARS-CoV-2, like other coronaviruses, depends on several viral enzymes essential for its replication and transmission, among which the main protease (Mpro, also known as 3-chymotrypsin-like protease or 3CLpro) stands out as a critical drug target [2,3].

Mpro plays a critical role in the life cycle of SARS-CoV-2 and other highly pathogenic coronaviruses, including SARS-CoV-1 and MERS-CoV. It is essential for processing the two large polyproteins, pp1a and pp1ab, into 16 non-structural proteins (NSPs) required for viral replication and transcription [4]. Specifically, Mpro cleaves these polyproteins at 11 conserved sites to generate key NSPs, such as the RNA-dependent RNA polymerase (RdRp), helicase, exoribonucleases, 2′-O-methyltransferase, and uridine-specific endoribonuclease [5]. The conserved nature of Mpro across coronaviruses and its indispensable role in viral replication make it an attractive drug target [6,7]. Inhibiting Mpro disrupts the maturation of NSPs, effectively halting the virus’s ability to replicate and spread. Furthermore, because there is no human homolog of Mpro, selective inhibitors can be designed with minimal risk of interfering with host proteins, bolstering the therapeutic potential of Mpro inhibitors for treating COVID-19 and other coronavirus-related diseases [8,9].

Structurally, Mpro functions as a homodimer composed of three distinct domains [7]. Domains I and II constitute the catalytic core featuring antiparallel β-barrels, while domain III is primarily α-helical and crucial for dimerization, a prerequisite for its enzymatic activity. The catalytic mechanism of Mpro involves a conserved dyad comprising cysteine (Cys145) and histidine (His41) residues, located at the interface between domains I and II. This catalytic dyad specifically recognizes and cleaves viral polyprotein substrates at sequences containing a glutamine residue at the P1 position. As described in Ref. [10], the active site includes several subsites (S1′, S1, S2, S3, and S4), each playing a crucial role in substrate recognition and inhibitor binding [11].

Antiviral inhibition strategies for Mpro include both covalent and non-covalent agents [10,12]. Covalent inhibitors, such as GC376 and nirmatrelvir, typically incorporate electrophilic warheads (e.g., aldehydes, α-ketoamides, nitriles, and haloacetamides) that form an irreversible covalent bond with the catalytic cysteine (Cys145). These compounds often proceed via a two-step mechanism [13,14]: first, reversible non-bonded interactions between the inhibitor and its interacting protein residues position the inhibitor appropriately; next, the nucleophilic thiol of Cys145 attacks the electrophilic center, forming a stable, often irreversible, adduct. In contrast, non-covalent inhibitors rely on non-bonded intermolecular interactions without forming covalent bonds. Each approach offers distinct advantages: covalent strategies can yield sustained inhibition, while non-covalent inhibitors often allow increased flexibility and fewer host toxicities.

Several clinically advanced therapeutics are based on Mpro inhibition [15], including nirmatrelvir (the active ingredient of Paxlovid™) [16], simnotrelvir (the active ingredient of Xiannuoxin), and ensitrelvir (S-217622) [17]. Additionally, many other compounds such as MG-101, Lycorine HCl, and Nelfinavir mesylate have been identified as potential Mpro inhibitors [18]. Nonetheless, the therapeutic effectiveness of Mpro inhibitors may compromise the emergence of drug-resistant viral variants and potential off-target effects. Mutations at residues such as S144, M165, E166, H172, or Q192 have been associated with reduced susceptibility to current drugs, including nirmatrelvir [19]. These mutations can modify the active site or dimer interface, thereby decreasing inhibitor-binding affinity. Moreover, some Mpro inhibitors, such as calpain inhibitors II and XII, also target host cathepsins [10], raising safety concerns due to unintended disruption of host proteolytic pathways.

These challenges underscore the critical need to develop next-generation Mpro inhibitors with enhanced selectivity and resistance to mutational escape. Addressing these issues requires a comprehensive understanding of the molecular determinants that govern molecular recognition of inhibitors by Mpro. Fortunately, the Protein Data Bank (PDB) contains a wealth of X-ray crystallographic structures of Mpro–inhibitor complexes, offering a unique opportunity to conduct a systematic study of molecular recognition of Mpro inhibitors in protein presented here.

In the present study, we analyze the molecular recognition of Mpro inhibitors using a multifaceted approach integrating data mining, cheminformatics, and high-level quantum chemical calculations. Initially, we mined the PDB to compile a dataset of 963 high-resolution Mpro–inhibitor complexes, comprising 348 covalent and 615 non-covalent inhibitors. This dataset served as the foundation for subsequent analyses.

Then, a cheminformatics analysis of all 963 inhibitors was performed, utilizing a curated set of molecular descriptors [20] as listed in Table 1. These include molecular weight (MW), number of hydrogen bond donors (nHDon), number of hydrogen bond acceptors (nHAcc), calculated logP (cLogP), number of rotatable bonds (RBN), and topological polar surface area (TPSA), which are key parameters influencing drug-likeness, permeability, and oral bioavailability. These descriptors align with established rules, such as Lipinski’s rule of five [21] and Veber’s rule [22], which evaluate drug-likeness based on physicochemical properties.

Beyond evaluating drug-likeness and structural features, this analysis sought to elucidate molecular features distinguishing covalent from non-covalent inhibitors. Additional descriptors such as the number of aromatic rings (nAR), aromatic ratio (ARR), and the fraction of sp^3^ carbon atoms (Fsp^3^) were examined to assess their roles in ligand binding. Hydrogen bonding potential, a pivotal factor in binding affinity, was also carefully considered.

Ultimately, the rational design of effective Mpro inhibitors hinges on understanding the detailed mechanisms of molecular recognition. In this context, we focused our attention on characterizing the non-bonded interactions between Mpro and its inhibitors. As in all ligand–protein complexes, the intricate molecular recognition between inhibitors and Mpro is facilitated through a variety of non-bonded interactions [23,24]. Historically, hydrogen bonding and salt bridges have been the primary focus of research. However, recent insights have highlighted the critical role of π-moiety-related interactions, which introduce additional complexity. These include π–π stacking interactions [25], CH–π interactions [26], cation–π interactions [27], and XH–π interactions [28,29,30] (with XH representing NH, OH, or SH groups). For clarity and ease of reference, we shall collectively denote all these π-moiety-related interactions as non-bonded π interactions hereinafter.

To gain deeper insights into the molecular recognition of Mpro inhibitors, we conducted a comprehensive analysis of a curated subset of 20 covalent and 20 non-covalent inhibitors, selected for their structural diversity and binding characteristics. A detailed examination of their binding pockets enabled us to map out non-bonded interactions (hydrogen bonds, salt bridges, π–π stacking, CH–π, cation–π, and XH–π interactions) that contribute to binding affinity. To quantify these non-bonded interactions, we employed the double-hybrid density-functional theory (DFT) method B2PLYP, coupled with the def2-QZVP basis set. The choice of the B2PLYP/def2-QZVP method is based on a systematic benchmark study [31]. It was found that the double-hybrid functional B2PLYP, in combination with the def2-QZVP basis set, is one of the best DFT methods for the treatment of non-bonded interactions in terms of both accuracy and computational efficiency in comparison with the highly accurate CCSD(T) method [31]. The main objective of this article is to decipher the key non-bonded interaction modes governing ligand recognition within the Mpro active site, as well as the distinct binding forces that distinguish covalent inhibitors from non-covalent inhibitors.

## 2. Results and Discussion

### 2.1. Data Mining and Classification of Mpro Inhibitors

An extensive data-mining analysis (see Section 3) was performed on the Protein Data Bank [32], resulting in a comprehensive dataset of 963 distinct high-resolution crystal complex structures of Mpro bound with inhibitors. These inhibitors were subsequently classified into two categories based on their binding mechanism: 348 covalent inhibitors, which form stable covalent bonds with Mpro, and 615 non-covalent inhibitors, which interact through reversible non-bonded interactions.

Detailed information about each of these 963 Mpro–inhibitor complexes, including the PDB ID, Ligand ID, Ligand Name, and the resolution of the respective X-ray crystal structures (in Å), is provided in Table S1 of the Supplementary Materials.

### 2.2. Cheminformatics Analysis of Mpro Inhibitors

#### 2.2.1. Molecular Descriptors: A Comparative Analysis of Covalent vs. Non-Covalent Inhibitors

A comprehensive cheminformatics analysis was conducted on the 963 distinct Mpro inhibitors identified through extensive data mining of the Protein Data Bank. For all 963 Mpro inhibitors listed in Table S1, molecular descriptors were calculated using the RDKit [33] library from the Python package 3.10.4, along with the cheminformatics programs Data Warrior 5.0 [34] and Dragon 6.0 [35], as described in Section 3. Statistical distributions of all calculated descriptors, including minimum, medium, maximum, and average values, are tabulated in Table 2. Figures S1 and S2 show the distribution of the calculated molecular descriptors in the form of histograms for both covalent and non-covalent inhibitors, respectively.

The molecular descriptors studied can be divided into two main categories [36]. The first category is the bioavailability group, which includes the following descriptors: molecular weight (MW), the number of hydrogen bond donor atoms (nHDon), the number of hydrogen bond acceptor atoms (nHAcc), the number of rotatable bonds (RBN), topological polar surface area (TPSA), and the calculated partition coefficient between octanol and water (cLogP). These descriptors are aligned with established pharmacokinetic criteria such as Lipinski’s rule of five and Veber’s rule, providing essential insights into drug-like properties and potential bioavailability [37]. The second category is the binding affinity related to 2D molecular descriptors. This group includes the fraction of sp3 hybridized carbon atoms (Fsp^3^), the aromatic ratio (ARR), and the number of aromatic rings (nAR). These descriptors, along with hydrogen bound counts, play significant roles in characterizing the binding affinity and potency of a compound.

It is the bioavailability descriptors that form the foundation of Lipinski’s rule of five [21]. It proposes that for a drug to exhibit optimal bioavailability, certain molecular characteristics should be met. Specifically, effective drug candidates should possess a molecular weight of less than 500 Daltons (Da), have five or fewer hydrogen bond donors, and 10 or fewer hydrogen bond acceptors. Additionally, the calculated logarithm of the octanol-water partition coefficient (cLogP) should be less than five, while the topological polar surface area should not exceed 140 Å^2^.

Detailed statistical analyses reveal significant physicochemical distinctions between covalent and non-covalent inhibitors. Covalent inhibitors exhibit notably higher molecular weights, averaging 442.32 Da (range: 112.15–993.30 Da), compared to non-covalent inhibitors, which average 353.76 Da (range: 73.12–630.50 Da). Adherence to Lipinski’s molecular weight criterion (<500 Da) is significantly higher among non-covalent inhibitors (91.1%) compared to covalent inhibitors (59.8%). Hydrogen bonding capacities also differ substantially; covalent inhibitors display greater hydrogen bonding potential with an average of 2.99 hydrogen bond donors and 5.19 acceptors compared to non-covalent inhibitors, which average 1.32 donors and 3.79 acceptors. Notably, as shown in Table 2, an impressive 96.3% of covalent inhibitors feature five or fewer hydrogen bond donors, while all non-covalent inhibitors adhere to Lipinski’s rule, which dictates that they should contain five or fewer donor atoms. Looking at hydrogen bond acceptors (nHAcc), covalent inhibitors demonstrate greater variability, with a minimum of 1 and a maximum soaring to 13. Approximately 99.4% of these inhibitors have 10 or fewer hydrogen bond acceptor atoms, indicating a tendency toward a structure that supports selectivity. On the other hand, non-covalent inhibitors span a range of 0 to 11 for nHAcc. Furthermore, 99.2% of non-covalent inhibitors satisfy the criteria of the rules of five (Ro5).

Both nHDon and nHAcc are critical factors that significantly affect a compound’s ability to bind with its protein target Mpro. Based on a comprehensive analysis, 271 covalent inhibitors (77.9%) and 608 non-covalent inhibitors (99.8%) demonstrated compliance with Veber’s rule. This rule specifies that the total number of hydrogen bond donors and acceptors in a molecule should be fewer than 12. Additionally, the topological polar surface area (TPSA) serves as a crucial metric for assessing a molecule’s polar surface properties. It provides insight into a compound’s potential to cross cell membranes, which is vital for drug absorption. TPSA is calculated by summarizing the surface areas of all polar atoms within the molecule, offering valuable predictions regarding the pharmacokinetic behavior—absorption and transport—of a particular drug [36]. Veber’s rule emphasizes the importance of Total Polar Surface Area (TPSA) and the number of rotatable bonds in determining a compound’s properties. Lower TPSA values (≤140 Å^2^) are linked to increased permeability, which consequently leads to better oral bioavailability. Among the compounds analyzed, 222 covalent inhibitors (63.8%) had a TPSA of ≤140 Å^2^, while 610 non-covalent inhibitors (99.3%) fell into the same category. The number of rotatable bonds in these compounds varies from 1 to 40. Covalent inhibitors have an average of 11 rotatable bonds, compared to non-covalent inhibitors, which average 4 with a range of 1 to 19. This descriptor reflects a compound’s molecular flexibility. Generally, compounds with 10 or fewer rotatable bonds (≤10) are associated with improved oral bioavailability. Veber et al. found that this measure is a more reliable predictor of oral bioavailability than molecular weight.

The logarithm of the octanol-water partition coefficient, known as cLogP, serves as an important indicator of a compound’s lipophilicity. This property significantly impacts both solubility and permeability, which are critical factors for drug absorption in biological systems. Compounds with a moderate level of lipophilicity, defined as having a cLogP value of less than five, typically achieve better bioavailability. This is because they strike a favorable balance between being soluble in aqueous environments and being able to permeate biological membranes effectively. In the analysis conducted, a striking 334 covalent inhibitors—accounting for 96% of the total analyzed—adhere to the principles outlined in Lipinski’s Rule of Five (Ro5) by maintaining a cLogP below the threshold of five. Similarly, around 576 non-covalent inhibitors, representing 93.7% of their category, also fell within the acceptable range set by Ro5. Overall, covalent inhibitors exhibited lower average lipophilicity (average cLogP of 2.12), whereas non-covalent inhibitors typically had higher lipophilicity (average cLogP of 3.22). Ranges were broad for both, spanning from −2.28 to 7.12 for covalent and from −1.49 to 7.24 for non-covalent inhibitors.

Binding affinity-related 2D descriptors also highlighted key structural differences. Non-covalent inhibitors were characterized by significantly higher aromatic content, averaging 2.59 aromatic rings (range: 0–6), compared to covalent inhibitors with an average of 1.41 aromatic rings (range: 0–5). The aromatic ratio (ARR)—calculated as the number of aromatic atoms divided by the total number of heavy atoms—was notably higher in non-covalent inhibitors (average 0.57) than covalent inhibitors (average 0.29), underscoring their propensity for engaging in non-bonded π interactions such as π–π stacking and CH–π interactions. Additionally, covalent inhibitors exhibited higher fractions of sp^3^ hybridized carbon atoms (Fsp^3^), averaging 0.50 compared to non-covalent inhibitors, which averaged 0.24. This difference highlights the greater structural rigidity of non-covalent inhibitors.

Remarkably, only about 0.5% of the non-covalent inhibitors analyzed had no aromatic rings, indicating their prevalent role in this class of compounds. In contrast, around 19.5% of the covalent inhibitors assessed lacked aromatic rings altogether. The covalent inhibitor group displayed a lower average of 1.41 aromatic rings, with a maximum count of five. The inclusion of aromatic rings in a molecular structure can greatly impact its binding properties through several mechanisms. These rings can engage in non-bonded π interactions with their interacting residues in the binding pocket, which promotes stability and binding affinity. Additionally, the structural rigidity provided by aromatic rings can diminish the entropic penalties associated with binding, which can lead to enhanced potency. Moreover, the presence of these rings contributes to increased lipophilicity, significantly influencing membrane permeability and the ability of the compound to bind to proteins, thereby affecting its overall efficacy. However, it is worth noting that an excessive number of aromatic rings can lead to decreased solubility and increased promiscuity, potentially reducing selectivity [38].

Overall, this detailed cheminformatics analysis delineates significant physicochemical and structural variations between covalent and non-covalent Mpro inhibitors. Notably, covalent inhibitors exhibit higher numbers of hydrogen bond donors and acceptors, enhancing their potential for hydrogen bonding interactions, whereas non-covalent inhibitors possess a significantly greater number of aromatic rings, facilitating non-bonded π-interactions critical for their binding affinity.

In the next section, we explore the relationship between aromatic rings and hydrogen bonds.

#### 2.2.2. Relationship Between Aromatic Rings and Hydrogen Bonds

In this analysis, we explored the fascinating relationship between aromatic rings and hydrogen bonding capabilities in SARS-CoV-2 Mpro inhibitors. Table 3 categorizes Mpro inhibitors by aromatic ring counts, elucidating distinct structural patterns between covalent and non-covalent inhibitors. As shown in Table 3, approximately 80.5% of the 348 covalent inhibitors and 99.5% of the 615 non-covalent inhibitors contain aromatic rings. Among covalent inhibitors, a majority (38.51%) possess one aromatic ring, while non-covalent inhibitors predominantly (47.97%) feature three aromatic rings. Compounds containing four or more aromatic rings are relatively less common, corresponding to previously documented challenges in drug development.

**Figure 1:**
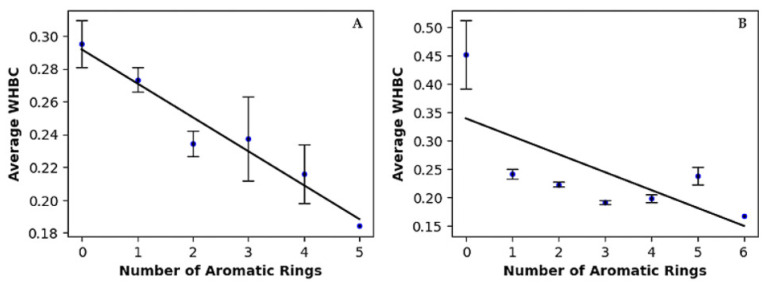
Linear regression of average of weighted hydrogen bond count versus number of aromatic rings for (**A**) covalent inhibitors and (**B**) non-covalent inhibitors.

Hydrogen bonding is widely recognized as a key contributor to ligand binding in proteins. To evaluate the contribution of hydrogen bonding to binding affinity independently of molecular size, we introduced an innovative molecular descriptor named Weighted Hydrogen Bond Count (WHBC) [36]. WHBC provides a clear representation of hydrogen bonding potential relative to molecular size by calculating the sum of hydrogen bond donors and acceptors normalized by the total number of non-hydrogen atoms in the molecule: (nHDon + nHAcc)/nSK. For each aromatic ring count, the average WHBC values were calculated and presented in the last column of Table 3. Figure 1 plots these average WHBC values against the number of aromatic rings, revealing a negative correlation: WHBC decreases as the number of aromatic rings increases. Covalent inhibitors (Figure 1A) exhibit a pronounced negative correlation, with data points tightly clustered along a descending regression line. In contrast, non-covalent inhibitors (Figure 1B) show a broader distribution of data points, indicating a somewhat weaker yet still significant negative correlation. Notably, a validation test conducted on a randomly selected dataset of 1000 compounds from the PubChem library revealed no direct correlation between WHBC and the number of aromatic rings (see Figure S3 and associated description in Supplementary Materials).

**Figure 2:**
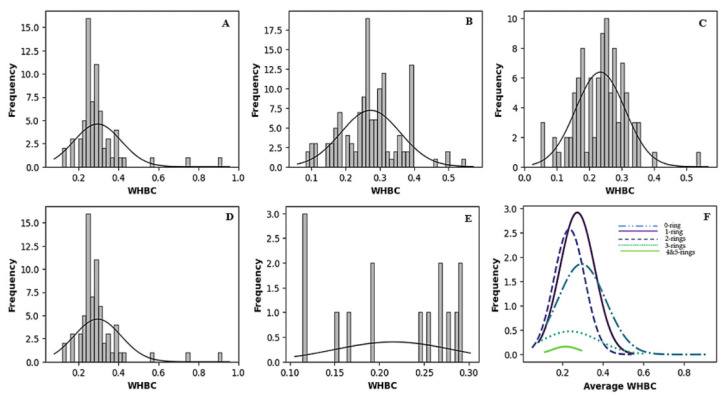
Histogram of the number of weighted hydrogen bond counts versus frequency for different classes of covalent inhibitors. (**A**) Zero aromatic rings, (**B**) one aromatic ring, (**C**) two aromatic rings, (**D**) three aromatic rings, (**E**) four or more aromatic rings, (**F**) normal distribution of the number of hydrogen bond count for the different classes of ligands.

**Figure 3:**
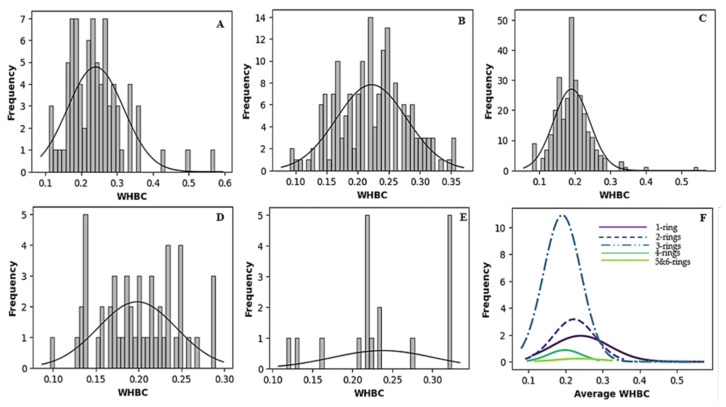
Histogram of the number of weighted hydrogen bond count versus frequency for different classes of non-covalent inhibitors. (**A**) One aromatic ring, (**B**) two aromatic rings, (**C**) three aromatic rings, (**D**) four aromatic rings, (**E**) five or more aromatic rings, (**F**) normal distribution of the number of hydrogen bond count for the different classes of ligands.

Figure 2 and Figure 3 vividly illustrate these trends through histograms. For covalent inhibitors (Figure 2), increasing aromatic ring numbers clearly shifted distributions towards lower WHBC values. The same general trend was observed for non-covalent inhibitors (Figure 3), although interesting fluctuations appeared, particularly noticeable at five aromatic rings.

In summary, our findings highlight a compelling interplay between non-bonded π interactions and the hydrogen bonding capability of inhibitors. The inverse correlation revealed in Figure 1 led us to propose an exchange rule between hydrogen bonding interactions and non-bonded π interactions. Hydrogen bonds are typically strong, directional interactions that play a significant role in binding affinity and specificity. As the number of hydrogen bonds decreases, we might expect a reduction in binding affinity. However, the overall binding affinity may be maintained or even improved due to the compensatory effects of aromatic ring-associated non-bonded interactions, such as π–π stacking, CH–π interactions, and cation-π interactions, which can collectively have a substantial impact (see results in Section 2.3 and Ref. [36]). The presence of this inverse relationship underscores the joint importance of hydrogen bonding and non-bonded π-interactions in molecular recognition between Mpro and its inhibitors. Thus, these non-bonded π interactions may significantly influence binding energetics, complementing the role traditionally attributed to hydrogen bonding.

### 2.3. Binding Modes of Covalent and Non-Covalent Inhibitors

#### 2.3.1. Comparative Analysis of Binding Modes

To elucidate distinct binding modes of SARS-CoV-2 Mpro inhibitors, we performed a detailed comparative analysis of 20 covalent and 20 non-covalent inhibitors. Table 4 provides an extensive list of the selected inhibitors categorized based on their binding mechanism and the methodology of selection. Each group of inhibitors was systematically selected to capture significant structural diversity and varied interaction profiles. Specifically, 10 inhibitors from each category were chosen using the Principal Component Analysis (PCA)-based clustering techniques [39], ensuring chemical heterogeneity that represented the broad chemical space occupied by Mpro inhibitors. The remaining 10 inhibitors in each category were selected based on the frequency of aromatic rings within their structures, ensuring a broad distribution of ring counts to analyze their impact on binding affinity and interaction specificity.

Based on the three-dimensional structures, the binding pockets of all 40 inhibitors within the Mpro enzyme were meticulously analyzed using the Visual Molecular Dynamics (VMD) program to identify residues that engage in non-bonded interactions with each inhibitor. Consistent with the physical nature of each type of non-bonded interaction, a cut-off distance of 3.5 Å between the donor and the acceptor was used for hydrogen bonding. For π–π stacking and CH–π interactions, cut-off distances of 5.6 Å and 5.0 Å were applied, respectively. Previous quantum chemical calculations indicated that solution-phase interaction energies for such π-related contacts generally diminish significantly beyond 5.6 Å [36]. The non-bonded interactions (hydrogen bonding, π–π stacking, and CH–π interactions) so identified were quantified by means of quantum chemical calculations next.

In order to assess the strength and the relative importance of different types of non-bonded interactions, the strengths of the intermolecular interaction energies for all non-bonded interactions were quantified at both the gas phase and the solution phase. The latter aims at a realistic evaluation of the strengths of the intermolecular interactions in aqueous media, where the actual biological interactions occur. The gas-phase interaction energies were calculated at the B2PLYP/def2-QZVP level with the basis set superposition error (BSSE) corrections (see Section 3 for details). The solution-phase interaction energies were obtained indirectly by means of a thermodynamic cycle (see Section 3): ${\Delta E}_{int}^{aq}={\Delta E}_{int}^{g}+{\Delta E}_{Deh}$. The dehydration energy ${\Delta E}_{Deh}$ itself was calculated utilizing the SM5.42R solvation model of Cramer and Truhlar.

As an illustration, two representative cases are presented in detail below. One representative case featured the covalent inhibitor F2F-2020198-00X, while the other contained the non-covalent inhibitor EDG-MED-10fcb19e-1. The binding pocket of the covalent inhibitor was analyzed using the high-resolution 1.35 Å X-ray crystal structure (PDB ID: 8OKN [40]), whereas the non-covalent inhibitor was examined using its corresponding 1.68 Å X-ray crystal structure (PDB ID: 7GL5 [41]). Our goal here is to provide a detailed comparative analysis of the interaction patterns for covalent and non-covalent inhibitors, providing valuable insights into their distinct molecular recognition mechanisms.

**Figure 4:**
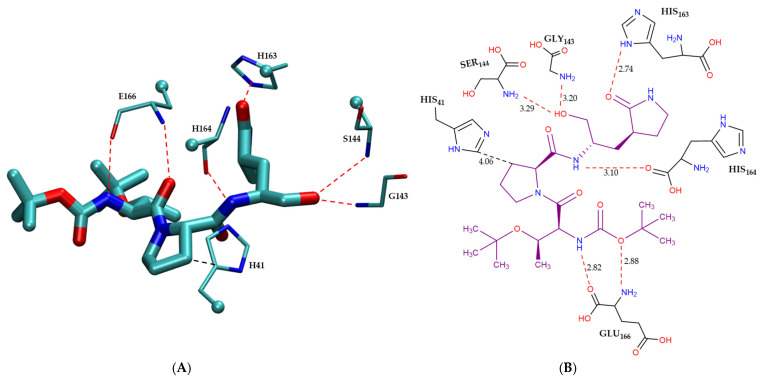
Modes of intermolecular interactions between the covalently bound F2F-2020198-00X and its interacting residues within the Mpro enzyme. (**A**) Structures of residues (in licorice representation) that are involved in hydrogen bonding interactions and CH–π interactions with F2F-2020198-00X (Ligand ID: 83W) based on the 1.35 Å resolution X-ray crystal structure (PDB ID: 8OKN [40]). Dashed lines indicate the closest interatomic distance. The structure was generated with the VMD program [42]. (**B**) A schematic intermolecular interaction map between F2F-2020198-00X and its interacting residues, with dashed lines indicating interatomic distances in Å (color code: hydrogen bonding interactions in red and CH–π interaction in black).

Figure 4 illustrates the intermolecular interactions between the covalent inhibitor F2F-2020198-00X and key interacting residues within the Mpro active site. Figure 4A shows the three-dimensional structural representation of residues involved in hydrogen bonding and CH–π interactions. Figure 4B offers a schematic two-dimensional interaction map, clearly depicting all identified interaction types. As indicated, F2F-2020198-00X predominantly engages the Mpro active site through hydrogen bonding. Residues Ser144, His164, His163, and Gly143 form hydrogen bonds with F2F-2020198-00X at interaction distances of 3.29 Å, 3.10 Å, 2.74 Å, and 3.20 Å, respectively. Notably, His163 establishes a hydrogen bond via its side chain, whereas the remaining residues utilize their main chains. Additionally, Glu166 participates with its main chain in forming a dual hydrogen bond with the inhibitor. Furthermore, His41 contributes to ligand stabilization through a CH–π interaction using its side chain, as detailed in Figure 4B.

**Figure 5:**
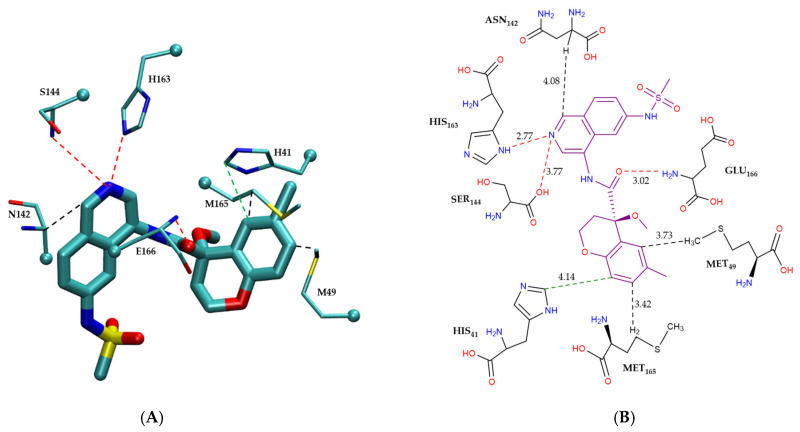
Modes of intermolecular interactions between the non-covalently bound EDG-MED-10fcb19e-1 and its interacting residues within the Mpro enzyme. (**A**) Structures of residues (in licorice representation) involved in hydrogen bonding, π–π stacking and CH–π interactions with EDG-MED-10fcb19e-1 (Ligand ID: R5O) based on the 1.68 Å resolution X-ray crystal structure (PDB ID: 7GL5 [41]). Dashed lines indicate the closest interatomic distance. The structure was generated with the VMD program [42]. (**B**) A schematic intermolecular interaction map between EDG-MED-10fcb19e-1 and its interacting residues, with dashed lines indicating interatomic distances in Å. (color code: hydrogen bonding interactions in red, CH–π interactions in black, and π–π interactions in green).

Figure 5 shows the modes of intermolecular interactions between EDG-MED-10fcb19e-1 and its interacting residues within the Mpro enzyme. Figure 5A presents the three-dimensional structure of the residues that engage in hydrogen bonding, π–π stacking interactions, and CH–π interactions with the non-covalent inhibitor, while Figure 5B provides a schematic two-dimensional intermolecular interaction map between the ligand and interacting residues, showing all modes of interactions. As shown in the figure, EDG-MED-10fcb19e-1 interacts with the Mpro enzyme primarily through π–π stacking and CH–π interactions, along with hydrogen bonds. Notably, the presence of aromatic rings on the ligand enables favorable π–π stacking interactions with His41. Additionally, three distinct CH–π interactions occur between EDG-MED-10fcb19e-1 and residues Asn142, Met49, and Met165. Furthermore, residues His163, Glu166, and Ser144 form hydrogen bonds with the ligand, further stabilizing its interaction within the active site, as illustrated in Figure 5B.

Additional examples highlighting distinct non-bonded interaction patterns between covalent and non-covalent inhibitors are presented in Figure S4.

The strengths of the non-bonded intermolecular energies between the two inhibitors and their surrounding residues inside Mpro were quantified in a pair-wise manner by means of the double-hybrid DFT method B2PLYP/def2-QZVP (see Section 3 for details). The resulting pairwise intermolecular interaction energies between F2F-2020198-00X and EDG-MED-10fcb19e-1 and their interacting residues are detailed in Tables S2 and S3, and categorized on the basis of interaction modes in Table 5 and Table 6.

Table S2 summarizes the intermolecular interaction energies of two distinct ligands, F2F-2020198-00X and EDG-MED-10fcb19e-1, with Mpro. Although both ligands exhibit multiple non-bonded interactions with Mpro residues, significant differences exist in their interaction modes and strengths. The covalent inhibitor F2F-2020198-00X predominantly engages in hydrogen bonding interactions, as detailed in Table S2. Among the interacting residues, E166 forms the strongest hydrogen bond with an interaction energy of −21.7 kcal/mol in the gas phase, which is reduced to −5.8 kcal/mol in the solution phase after significant dehydration energy correction (${\;\Delta E}_{Deh}$). This pronounced interaction strength emphasizes the crucial role of E166 in stabilizing the ligand–protein complex.

In contrast, the non-covalent inhibitor EDG-MED-10fcb19e-1 exhibits a diverse interaction profile, including π–π stacking, CH–π interactions, and hydrogen bonding (Table S3). The interaction profile of this ligand is predominantly hydrophobically driven, facilitated by the presence of aromatic rings, which enable significant non-bonded π interactions. Specifically, π–π stacking occurs between the ligand’s aromatic ring and the imidazole ring of histidine residues, enhancing ligand stability. Additionally, CH–π interactions are observed between the ligand’s π-electron cloud and the C-H groups of methionine residues (M49 and M165), as well as asparagine (N142), further contributing to hydrophobic stabilization.

The relative sensitivities of these interactions to solvent conditions also vary significantly. Hydrogen bonds are highly susceptible to disruption by water molecules due to competitive hydrogen bonding. As indicated in Table S2, hydrogen bonding interactions incur substantial dehydration penalties (ΔE_Deh_), weakening their overall strength in aqueous environments. Conversely, CH–π and π–π interactions exhibit lower dehydration costs, making them less influenced by solvent displacement. Consequently, EDG-MED-10fcb19e-1 benefits from stronger hydrophobic stabilization, maintaining interaction energy more effectively under aqueous conditions [43].

Table 5 and Table 6 tabulate the contributions of specific intermolecular interaction types to the overall binding affinities of both ligands with Mpro. Hydrogen bonding contributes significantly (−12.4 kcal/mol), accounting for approximately 82.1% of the total binding affinity for F2F-2020198-00X, whereas non-bonded π interactions contribute only −2.7 kcal/mol (17.9%). In contrast, EDG-MED-10fcb19e-1 relies heavily on non-bonded π interactions (CH–π and π–π stacking), contributing −12.6 kcal/mol (65.6%), compared to −6.0 kcal/mol from hydrogen bonding.

Similarly, each of the remaining 38 Mpro inhibitors was analyzed individually using the same approach applied to the two representative inhibitors. Comprehensive binding energy analysis uncovered significant differences between the binding mechanisms employed by covalent and non-covalent inhibitors. The last column of Table 4 lists solution-phase interaction energies ${\Delta E}_{Int}^{aq}$, calculated at the B2PLYP/def2-QZVP level of theory. Summarizing ${\Delta E}_{Int}^{aq}$ in Table 4, 20 covalent inhibitors yielded a total calculated binding energy of −241.93 kcal/mol, averaging approximately −12.1 kcal/mol per inhibitor. Within these covalent inhibitors, hydrogen bonding was the predominant mode of interaction, contributing notably to their stability by accounting for approximately 63.84% of the total interaction energy. A total of 108 intermolecular interactions were observed, 77 of which were hydrogen bonds (H-bonds). Contrastingly, non-bonded π interactions (including 6 π–π stacking and 24 CH–π interactions, as well as 1 NH–π interaction) contributed significantly less, accounting for approximately 36.16% of the overall interaction energy. This disparity in interaction-type contribution is directly linked to the aromatic content of the inhibitors; covalent inhibitors presented an average of 1.75 aromatic rings per molecule, limiting their potential for extensive non-bonded π interactions. Consequently, covalent inhibitors appeared to compensate for this limitation by forming robust hydrogen bond networks facilitated by interactions with polar residues such as Glu166, His164, Gly143, and His163, reinforcing ligand stability within the active site.

In marked contrast, non-covalent inhibitors demonstrated significantly stronger total binding energy of −289.48 kcal/mol, with an average solution-phase interaction energy (${\Delta E}_{Int}^{aq})$ of −14.47 kcal/mol per inhibitor (see Table 4). This notable increase in binding strength underscores their reliance on non-bonded π interactions as a primary mode of binding stabilization. The analysis revealed 116 total interactions, with 50 hydrogen bonds and a significantly larger number of non-bonded π interactions (14 π–π stacking, 51 CH–π interactions, and 1 NH–π interaction). Hydrogen bonding played a less dominant role, accounting for only 37.16% of the total interaction energy. In contrast, π–π stacking and CH–π interactions emerged as the primary stabilizing forces, contributing a substantial 62.84% (−181.92 kcal/mol). Supporting these findings, non-covalent inhibitors presented a higher average aromatic ring count of approximately 2.55 per ligand. This higher aromatic content significantly enhanced their capacity for engaging in extensive and energetically favorable non-bonded π-interactions, predominantly involving critical residues such as His41, Met49, Met165, and Asn142. Such non-bonded π interactions substantially strengthened binding affinities through stabilized hydrophobic interactions and aromatic stacking within the Mpro active site.

Table 7 presents a comparative overview of the energetic statistics for both covalent and non-covalent inhibitors. In this table, the first and second columns list the interacting Mpro residues and the types of non-bonded interactions with the inhibitors, respectively. The third column indicates how frequently each type of non-bonded interaction occurs, as not all 20 inhibitors necessarily interact with every residue. Finally, the fourth column provides the average solution-phase interaction energy for the specified non-bonded interaction.

As can be seen from Table 7, the molecular recognition of SARS-CoV-2 Mpro inhibitors hinges on a set of critical residues that govern ligand binding and stabilization. These residues can be broadly classified into those commonly involved in stabilizing both covalent and non-covalent inhibitors and those uniquely important for one inhibitor class. Understanding these interactions not only clarifies the mechanistic underpinnings of Mpro inhibition but also serves as a blueprint for designing both broad-spectrum and highly specific inhibitors.

Our comprehensive analysis of multiple covalent and non-covalent inhibitors underscores the significance of four residues consistently involved in stabilizing both inhibitor classes, namely H41, S144, H163, and E166. Among these, H41 often engages in π–π stacking or CH–π interactions with the aromatic portions of the inhibitors, thereby leveraging ring-based contacts for enhanced stability and binding affinity. E166, H163, and S144 principally form hydrogen bonds—either via side chains (H163) or backbone atoms (S144 and E166)—reinforcing the integrity of the binding pocket. These polar contacts, in tandem with ring-based interactions, point to a synergistic interplay between hydrogen bonding and non-bonded π interactions in stabilizing the ligands. The ability of these four residues to facilitate multiple interaction modes underscores their importance in molecular recognition and suggests they are high-value targets for designing inhibitors that retain potency across different Mpro variants [44].

Beyond these shared residues, certain residues proved uniquely critical for covalent inhibitor stabilization. G143 and H164, in particular, consistently participated in hydrogen bonding with covalent ligands. G143 frequently helped orient the inhibitor so that its reactive (warhead) moiety was precisely aligned with C145, the catalytic residue, enabling robust and long-lived covalent bond formation. H164 further stabilized these ligands by maintaining productive hydrogen bonds, securing their reactive groups in an ideal position to engage with C145 [45]. These residues are critical in facilitating covalent modifications, making them valuable targets for designing highly specific and potent inhibitors.

In contrast, non-covalent inhibitors displayed a more pronounced reliance on aromaticity and hydrophobic surfaces, evidenced by the critical role of N142, M49, and M165. Through CH–π interactions with the inhibitors’ aromatic rings, these residues provided significant additional stabilization while allowing for reversibility of binding. Notably, Met49 and Met165 formed especially strong CH–π contacts in multiple non-covalent ligands, offering hydrophobic anchoring without the need for a covalent linkage. The adaptability of these residues in accommodating diverse aromatic scaffolds highlights their potential as design nodes for next-generation non-covalent antivirals, particularly those aimed at achieving potent but reversible inhibition [46].

Quantitative evaluation of intermolecular energies further clarifies these distinctions. Covalent inhibitors typically feature fewer aromatic rings and thus compensate by forming strong hydrogen bonds, particularly with residues E166, H163, G143, S144, and H164. This compensatory mechanism balances their reduced capacity for π-mediated interactions. In contrast, non-covalent ligands typically featured more extended aromatic cores, enabling multiple π–π and CH–π interactions (often with H41, M49, and M165). Although these π-based contacts are individually weaker than strong hydrogen bonds, they become collectively significant in aqueous media due to their lower dehydration penalties and additive nature. Taken together, these observations reveal two complementary routes to high-affinity binding: (1) a covalent linkage complemented by robust hydrogen bonding and (2) extensive π–π and CH–π contacts supporting a non-covalent, but often equally potent, inhibitor–Mpro interface.

In summary, a multi-faceted computational approach was employed to study the molecular recognition of Mpro inhibitors in proteins. A large-scale data mining of the Protein Data Bank yielded an in-house database of 963 non-redundant, high-resolution crystal structures of Mpro inhibitors bound to proteins. Notably, the dataset comprises 348 covalent inhibitors and 615 non-covalent inhibitors, which provides us with a unique opportunity to perform a comparative study of molecular recognition of covalent inhibitors against non-covalent inhibitors. A systematic analysis of non-bonded intermolecular interactions, including hydrogen bonding, π–π stacking, CH–π interactions, NH–π interactions, OH–π interactions, and cation-π interactions, together with subsequent quantification of strengths of those non-bonded interactions, provided key insights into the molecular recognition underpinning Mpro inhibitor binding. These insights bolster the development of the following pharmacophore models for Mpro inhibition.

##### Covalent Inhibitor Pharmacophore Model

**Figure 6:**
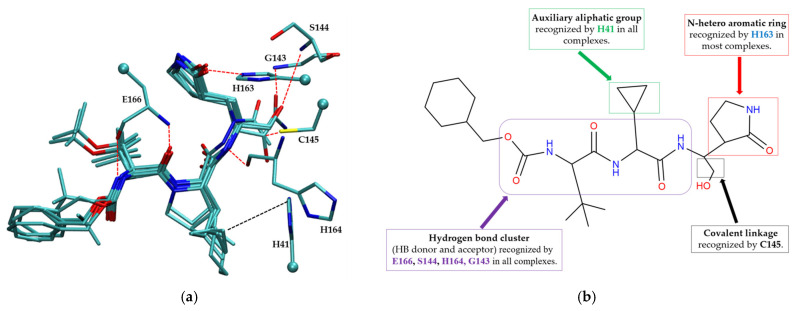
Pharmacophore model for covalent inhibitors. (**a**) Three-dimensional alignment of covalent inhibitors showing pharmacophore model with interacting residues. For clarity, only ligands 83W, 8Z1, 7VB, 7YI, GJ3, and 81L are shown (color code: hydrogen bonding interactions in red and CH–π interactions in black), (**b**) 2D schematic representation of the pharmacophore model. Residues G143, S144, H164, and E166 form hydrogen bonds with the hydrogen bond cluster, H41 forms CH–π interaction with the auxiliary aliphatic group, and H163 forms hydrogen bond with the oxygen of the aromatic amide.

The pharmacophore model for covalent inhibitors, depicted in Figure 6, is characterized by four essential interaction motifs. First, a covalent bonding motif anchors the inhibitor to the catalytic residue C145, irreversibly inhibiting the enzymatic activity of Mpro. Second, a hydrogen-bonding cluster, comprising multiple hydrogen bond donors and acceptors, interacts specifically with residues G143, S144, H164, and E166, thus stabilizing the inhibitor’s precise orientation within the active site. Third, an N-heteroaromatic ring facilitates hydrogen bonding with the ε-nitrogen atom of residue H163. Finally, an auxiliary hydrophobic motif provides additional stabilization to the inhibitor–Mpro complex through CH–π interactions with residue H41.

##### Non-Covalent Inhibitor Pharmacophore Model

**Figure 7:**
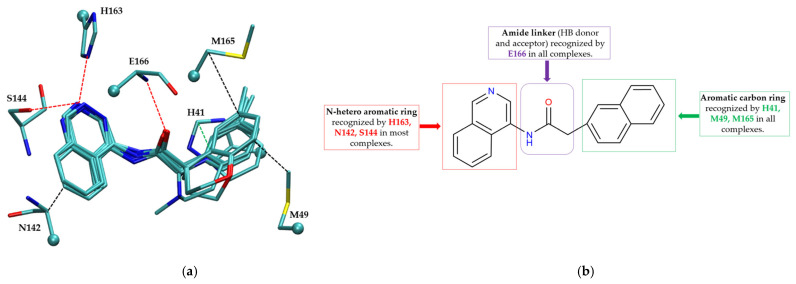
Pharmacophore model for non-covalent inhibitors. (**a**) Three-dimensional alignment of non-covalent inhibitors showing pharmacophore model with interacting residues. For clarity, only ligands KZX, M50, OSI, QT3, QM9, NQO, and NJE are shown (color code: hydrogen bonding interactions in red, CH–π interactions in black and π–π interaction in green), (**b**) 2D schematic representation of the pharmacophore model. H163 and S144 form hydrogen bonds with the nitrogen atom on the N-hetero aromatic ring, and the N-hetero aromatic ring is involved in CH–π interactions with N142. E166 forms hydrogen bond with the amide linker, and H41 forms π–π interaction, while M49 and M165 form CH–π interaction with the aromatic carbon ring.

The non-covalent inhibitor pharmacophore model, as shown in Figure 7, features an amide linker flanked by two aromatic motifs: an N-heteroaromatic ring on one side and an aromatic carbon ring on the other. The aromatic carbon ring establishes key π–π stacking interactions with residue H41 and engages in CH–π interactions with residues M49 and M165, collectively constituting the primary contributors to binding affinity. Complementing these interactions, the amide linker forms hydrogen bonds with residue E166, enhancing the stability of the inhibitor within the active site. Additionally, the nitrogen atom of the N-heteroaromatic ring facilitates multiple hydrogen bonds with residues S144 and H163, and the N-hetero aromatic ring is involved in CH–π interactions with N142. To a certain extent, the presence of extensive π–π stacking and CH–π interactions compensates effectively for the lack of covalent bonding, underscoring their essential role in stabilizing the non-covalent inhibitor–Mpro complex.

These detailed pharmacophore models, emphasizing motif–residue interactions, significantly enhance our understanding of inhibitor binding specificity. The models illustrate how effective inhibitor design requires strategic integration of aromatic functionalities and hydrogen bond-capable groups to optimize interaction strength, specificity, and therapeutic efficacy against SARS-CoV-2 Mpro.

From a drug design perspective, these findings highlight the importance of optimizing the number of aromatic rings to balance hydrogen bonding and π interactions for maximum binding efficiency. While covalent inhibitors achieve stability through direct bond formation and strong hydrogen bonding, non-covalent inhibitors benefit from aromatic stacking and hydrophobic stabilization. Incorporating both π interactions and hydrogen bonding elements in future drug candidates may lead to more potent, selective, and bioavailable Mpro inhibitors.

## 3. Theory and Methods

### 3.1. Data Mining of Mpro Inhibitors

A large-scale data mining was conducted on the Protein Data Bank to create a database of inhibitors that bind to the Mpro enzyme. Only high-resolution X-ray crystal structures of Mpro complexed with bound inhibitors, specifically those with a resolution of 2.8 Å or better, were selected for further analysis. This resulted in a total of 963 distinct high-resolution crystal structures of Mpro–inhibitor complexes. A systematic analysis of these 963 complexes identified 348 inhibitors as covalent, forming stable covalent bonds with Mpro, and 615 inhibitors as non-covalent, interacting through reversible non-covalent interactions with Mpro.

### 3.2. Cheminformatics Analysis: Molecular Descriptors

The RDKit [33] library from the Python package 3.10.4, along with the cheminformatics programs Data Warrior 5.0 [34] and Dragon 6.0 [35], were used to determine a variety of molecular descriptors for all 963 Mpro inhibitors. These descriptors include: molecular weight (MW), number of HB donors (nHDon), number of HB acceptors (nHAcc), total surface area (SA), topological polar surface area (TPSA), number of non-H atoms (nSK), number of aromatic atoms (nAA), number of rotatable bonds (RBN), aromatic ratio (ARR), calculated partition coefficient between octanol and water (cLogP), number of aromatic rings (nAR), and the fraction of sp^3^ carbon atoms (Fsp^3^). Additionally, the weighted hydrogen bond count (WHBC) was calculated using the formula [(nHDon + nHAcc)/(nSK)].

### 3.3. Quantum Chemical Calculations of Intermolecular Interaction Energies

The conceptual framework for the ligand–protein complex formation in solution is illustrated by the following scheme:

(1) $$P(\mathrm{aq})\;\;\;\;\;+\;\;\;\;\;L(\mathrm{aq})\;\overset{{\Delta E}_{int\;}^{aq}}{\to }\;\;\;\;PL(aq)\;{\Delta G}_{P}^{sol}↑\;{\;\;\;\;\;\;\;\Delta G}_{L}^{sol}↑\;\;\;\;\;\;\;\;\;\;\;\;\;\;\;\;\;\;↑{\Delta G}_{PL}^{sol}\;P(g)\;\;\;\;\;\;+\;\;\;\;\;\;L(g)\;\;\;\underset{{\Delta E}_{int\;}^{g}}{\to }\;\;\;\;PL(g)$$

This served as a foundation for our analysis of the binding affinity of inhibitors in the main protease enzyme. It is important to note that a similar approach was previously employed to calculate solution-phase binding affinities for various ligand–protein complexes [36,47,48]. Initially, both the ligand and protein exist in a hydrated environment, stabilized by surrounding water molecules. Upon binding, partial displacement of this hydration shell occurs, introducing an energetic penalty known as the dehydration energy. The binding energy in solution was thus evaluated via gas-phase intermolecular interaction energy ${\Delta E}_{Int}^{gas}$ corrected for dehydration energy ${\;\Delta E}_{Deh}$:

(2) $${\Delta E}_{Int}^{aq}={\Delta E}_{Int}^{gas}+{\Delta E}_{Deh}$$

Gas-phase interaction energies were calculated using the supermolecular approach. In this approach, the energy of interaction between the protein (P) and ligand (L) is defined as the difference between the energy of the interacting dimer $E_{PL}$ and the energies of the monomers $E_{P}$ and $E_{L}$.

(3) $${\Delta E}_{Int}^{gas}=E_{PL}-E_{P}-E_{L}$$

**Figure 8:**
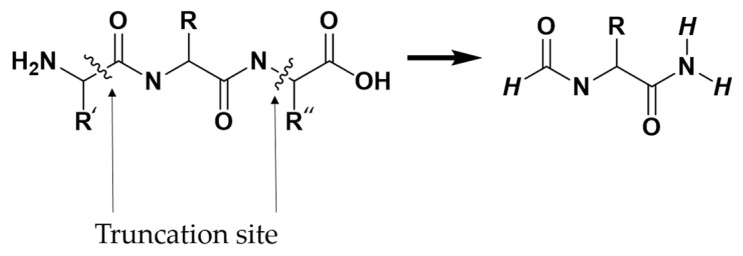
The –(CO)_i−1_–N_i_–Cα_i_–(CO)_i_–N_i+1_– scheme for truncating amino acids.

Due to the extensive number of atoms present in the protein, performing a full quantum-chemical calculation of the total interaction energy between a ligand and the entire protein was computationally infeasible. Instead, interaction energies were computed through multiple pairwise intermolecular interaction models. Each pairwise model included the complete ligand (L) and a single interacting protein residue (P). As illustrated in Figure 8, protein residues selected for interaction energy calculations were truncated following the –(CO)_i−1_–N_i_–Cα_i_–(CO)_i_–N_i+1_– scheme, where “i” represents the residue number of interest. Thus, each truncated residue comprised the backbone C and O atoms of the preceding residue (i − 1), the N, Cα, C, and O atoms of the residue itself (i), and the N atom of the succeeding residue (i + 1), along with the side chain atoms of residue i. Hydrogen atoms were patched at the truncation sites to satisfy the valence.

Coordinates for heavy (non-hydrogen) atoms in all pairwise intermolecular models were taken directly from the corresponding crystal structures. Protonation states for all residues were assigned, and missing hydrogen atoms were added using the PlayMolecule protein preparation tool [49]. Subsequently, the positions of hydrogen atoms were optimized using ab initio geometry optimization at the HF/6-31+G* level of theory, with heavy atoms constrained to their original positions. These optimizations were conducted using the Gaussian 16 software package [50].

The calculations of intermolecular interaction energy were conducted using the ORCA 4.0 program [51] and employed the double-hybrid density functional method B2PLYP [52,53], along with the def2-QZVP basis set [54]. To account for dispersion forces and long-range electron correlation effects, Grimme’s D3BJ dispersion correction [55] was applied. For improved efficiency, the B2PLYP method was implemented using the resolution of identity (RI) approximation during the perturbation step, and RIJK [56] was used for the SCF step. Additionally, the basis set superposition error (BSSE) was corrected using the Boys and Bernardi’s Counter Poise Method [57]. The energy of dehydration was estimated using the SM5.42R solvation continuum model developed by Cramer and Truhlar [58], applied at the Hartree–Fock level of theory.

(4) $${\Delta E}_{Deh}={\Delta G}_{PL}^{Sol}{-\Delta G}_{P}^{Sol}-{\Delta G}_{L}^{Sol}$$

## 4. Conclusions

In this comprehensive study, we systematically investigated the molecular recognition mechanisms of SARS-CoV-2 main protease (Mpro) inhibitors using an integrative framework encompassing large-scale data mining, cheminformatics analysis, and high-level quantum chemical calculations. A curated dataset of 963 high-resolution Mpro-inhibitor complexes—comprising 348 covalent and 615 non-covalent inhibitors—served as the basis for comparative analysis between these two distinct inhibitor classes.

### 4.1. Major Findings and Comparative Insights

Cheminformatics profiling revealed fundamental physicochemical differences between covalent and non-covalent inhibitors. Covalent inhibitors exhibited greater numbers of hydrogen bond donors (mean 2.99) and acceptors (mean 5.19), and a higher fraction of sp^3^-hybridized carbon atoms (Fsp^3^ ≈ 0.50). In contrast, non-covalent inhibitors displayed increased aromaticity, with an average of 2.59 aromatic rings per molecule and a higher aromatic ratio (ARR ≈ 0.57), accompanied by a lower Fsp^3^ (≈0.24), signifying greater molecular planarity and rigidity. A statistically significant inverse correlation was observed between the number of aromatic rings and the weighted hydrogen bond count (WHBC), indicating a compensatory relationship wherein aromatic π systems substitute for extensive hydrogen bonding. This trend was particularly pronounced in non-covalent inhibitors, where 99.5% of compounds contained at least one aromatic ring, underscoring the critical role of π-mediated interactions in binding affinity.

To elucidate mechanisms of molecular recognition, a representative set of 20 covalent and 20 non-covalent inhibitors was methodically selected based on structural diversity (via PCA-based clustering) and aromatic ring frequency. This dual selection strategy ensured comprehensive sampling of chemical space and interaction modes, enabling robust comparison of binding energetics across the two inhibitor classes. A comprehensive evaluation of non-bonded intermolecular interactions—encompassing hydrogen bonds, π–π stacking, CH–π, NH–π, OH–π, and cation–π interactions—along with a quantitative assessment of their binding energies, yielded critical insights into the molecular recognition mechanisms governing binding of Mpro inhibitor. It was found that covalent inhibitors derive most of their binding strength from hydrogen bonding (~63.8% of total binding energy), especially with residues E166, H163, S144, and G143, which support covalent bond formation with C145. In contrast, non-covalent inhibitors are dominated by π–π stacking and CH–π interactions (~62.8% of binding energy), largely involving residues such as H41, M49, M165, and N142, thereby relying on aromatic-driven stabilization within the binding cleft.

### 4.2. Pharmacophore Models

This work led to the establishment of two distinct pharmacophore models to encapsulate the key interaction motifs for each inhibitor class:

- **Covalent Inhibitor Pharmacophore Model**
  The covalent pharmacophore is characterized by:
  1. **Covalent anchor point** for nucleophilic attack by Cys145 (e.g., nitrile, aldehyde, or α-ketoamide warheads);
  2. **Hydrogen bond cluster** involving polar groups interacting with backbone atoms of residues G143, S144, H164, and E166, stabilizing ligand positioning;
  3. **N-heteroaromatic ring** enabling a directional hydrogen bond with the side chain of H163;
  4. **Auxiliary hydrophobic motif** capable of CH–π interaction with H41 to enhance hydrophobic stabilization.
- **Non-Covalent Inhibitor Pharmacophore Model**
  The non-covalent pharmacophore emphasizes:
  1. **Aromatic scaffold** flanking an amide linker, providing π–π stacking with H41 and CH–π interactions with M49 and M165;
  2. **Amide linker functionality** forming hydrogen bonds with E166, a conserved interaction point for ligand anchoring;
  3. **N-heteroaromatic ring** engaging in both CH–π interaction with N142 and hydrogen bonding with S144 and H163;
  4. **Balanced π-stacking and polar features**, allowing strong yet reversible binding optimized for selectivity and bioavailability.

### 4.3. Implications for Rational Drug Design

These findings offer critical insights into the rational design of next-generation Mpro inhibitors. Covalent inhibitors benefit from stable, irreversible binding augmented by robust hydrogen bond networks, favoring sustained inhibition. Non-covalent inhibitors, while reversible, exploit aromaticity to achieve high-affinity binding through π-mediated interactions with reduced solvation penalties. The dual pharmacophore models serve as design templates to optimize selectivity, potency, and resistance evasion by targeting conserved Mpro residues through distinct interaction mechanisms. The molecular level insights gained here encourage the future design of hybrid molecules that combine covalent warheads with aromatic-rich frameworks, enabling potent, selective, and mutation-resilient therapeutics against SARS-CoV-2 and emerging coronaviruses.

## Figures and Tables

**Table 1:** List of studied molecular descriptors.

Name	Description
MW	Molecular weight
nHDon	Number of hydrogen bond donors
nHAcc	Number of hydrogen bond acceptors
SA	Total surface area
TPSA	Topological polar surface area
nSK	Number of non-hydrogen atoms
RBN	Number of rotatable bonds
nAR	Number of aromatic rings
cLogP	Calculated partition coefficient between octanol and water
NAA	Number of Aromatic atoms
ARR	Aromatic ratio
Nsp^3^	Number of sp^3^ hybridized carbon atom
Fsp^3^	Fraction of sp^3^ carbon atoms

**Table 2:** Comparison of the statistical distribution of molecular descriptors.

Covalent Mpro Inhibitors						
Molecular Descriptor	Min	Median	Max	Average	Ro5 ^a^	Veber ^b^
MW (Da)	112.15	473.55	993.30	442.32 ± 7.69	59.8%	
nHDon	0	4	9	2.99 ± 0.11	96.3%	77.9% ^c^
nHAcc	1	5	13	5.19 ± 0.11	99.4%	
RBN	1	11	40	10.27 ± 0.30		49.1%
cLogP	−2.28	1.86	7.12	2.12 ± 0.08	96.0%	
NAA	0	6	25	7.83 ± 0.30		
ARR	0	0.27	0.92	0.29 ± 0.01		
SA (Å^2^)	129.80	533.26	1163.24	492.71 ± 8.84		
nAR	0	1	5	1.41 ± 0.06		
TPSA (Å^2^)	0	116.76	300.78	111.61 ± 2.77		63.8%
nSK	7	33	67	31.02 ± 0.55		
Nsp^3^	0	12	36	11.80 ± 0.39		
Fsp^3^	0	0.52	1	0.50 ± 0.01		
**Non-covalent Mpro inhibitors**						
**Molecular Descriptor**	**Min**	**Median**	**Max**	**Average**	**Ro5 ^a^**	**Veber ^b^**
MW (Da)	73.12	345.79	630.50	353.76 ± 3.80	91.1%	
nHDon	0	1	5	1.32 ± 0.03	100%	98.9% ^c^
nHAcc	0	4	11	3.79 ± 0.06	99.2%	
RBN	1	4	19	4.66 ± 0.09		98.9%
cLogP	−1.49	3.32	7.24	3.22 ± 0.05	93.7%	
NAA	0	16	30	14.23 ± 0.20		
ARR	0	0.59	0.86	0.57 ± 0.01		
SA (Å^2^)	117.44	356.86	664.73	367.64 ± 3.61		
nAR	0	3	6	2.59 ± 0.04		
TPSA (Å^2^)	12.27	64.11	167.86	67.77 ± 0.96		99.3%
nSK	5	24	44	24.84 ± 0.26		
Nsp^3^	0	4	19	4.37 ± 0.11		
Fsp^3^	0	0.2	1	0.24 ± 0.01		

^a^ Percentage of molecular descriptors that obey Lipinski’s rule of five (Ro5); ^b^ percentage of molecular descriptors that obey Veber’s rule. ^c^ Based on combined count of hydrogen bond donors and acceptors.

**Table 3:** Distribution of Mpro inhibitors by number of aromatic rings and corresponding average WHBC.

Covalent Mpro Inhibitors			
Number of Aromatic Rings	Number of Ligands	Percentage (%)	Average of WHBC
0	68	19.54	0.295 ± 0.014
1	134	38.51	0.273 ± 0.008
2	99	28.45	0.234 ± 0.008
3	31	8.91	0.238 ± 0.026
4	15	4.30	0.216 ± 0.018
5	1	0.29	0.184 ± 0.000
**Non-covalent Mpro inhibitors**			
**Number of Aromatic Rings**	**Number of Ligands**	**Percentage (%)**	**Average of WHBC**
0	3	0.49	0.452 ± 0.060
1	81	13.17	0.240 ± 0.009
2	166	26.99	0.222 ± 0.004
3	295	47.97	0.191 ± 0.003
4	51	8.29	0.198 ± 0.007
5	18	2.93	0.238 ± 0.016
6	1	0.16	0.167 ± 0.000

**Table 4:** List of Mpro inhibitors studied.

Covalent Inhibitors					
Selection Method	PDB ID	Resolution (Å)	Ligand ID	Ligand Structure	${\Delta E}_{Int}^{aq}$ (kcal/mol)
PCA clustering	5RFJ	1.8	T7A	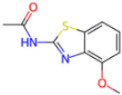	−3.5
	5RGN	1.86	U1A	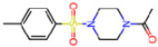	−3.6
	7AWU	2.07	S8B	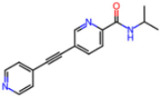	−3.7
	7GDX	1.84	N1U	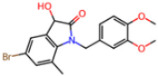	−11.0
	7GFV	1.73	OJO	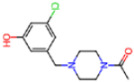	−4.5
	7UUC	1.6	81L	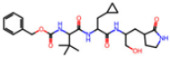	−16.2
	7SF1	1.85	8ZI	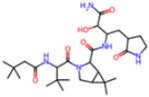	−13.7
	8BGA	1.98	QQL	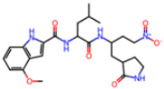	−12.1
	8OKN	1.35	83W	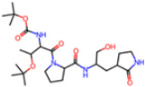	−15.1
	8TPE	1.61	JK0	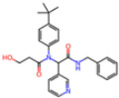	−12.1
Frequency of aromatic rings	5RFO	1.83	T7S	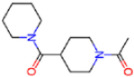	−3.7
	6XR3	1.45	V7G	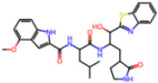	−13.7
	7FAZ	2.1	2RI	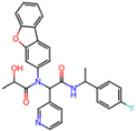	−21.2
	7GJ7	1.88	Q0I	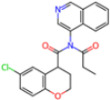	−14.0
	7NT1	2.85	UQW	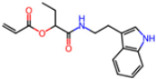	−13.2
	7RVN	1.63	7VB	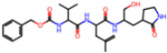	−13.5
	7RVX	1.85	7YI	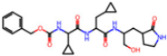	−16.1
	7SH8	1.8	GJ3	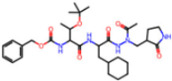	−17.0
	8DSU	1.86	V2M	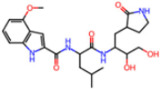	−19.9
	8GXI	1.69	0BO	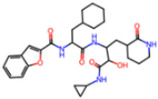	−14.1
**Non-covalent Inhibitors**					
**Selection Method**	**PDB ID**	**Resolution (Å)**	**Ligand ID**	**Ligand Structure**	${\Delta E}_{Int}^{aq}$ **(kcal/mol)**
PCA clustering	5REV	1.6	T4J	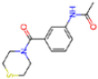	−3.3
	7GDJ	1.77	M50	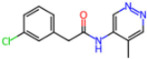	−15.6
	7GEF	1.18	NJE	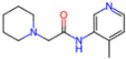	−10.7
	7GEM	1.32	NQO	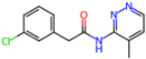	−13.0
	7GGA	1.49	OSI	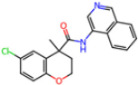	−18.1
	7GI4	1.72	QCC	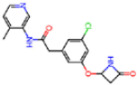	−18.7
	7GJ2	1.87	QK3	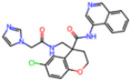	−19.5
	7GK3	2.19	QT3	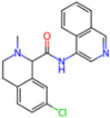	−18.1
	7GKV	1.88	R0F	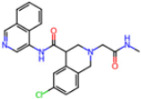	−20.0
	7GL5	1.68	R5O	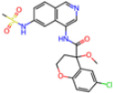	−19.2
Frequency of aromatic rings	5RF2	1.53	HVB	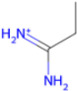	−1.2
	5RG1	1.65	T9J	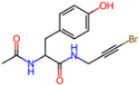	−11.3
	7ANS	1.7	RNW	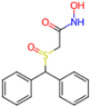	−8.3
	7GAZ	1.75	KL6	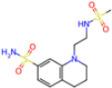	−3.2
	7GBS	1.54	KZX	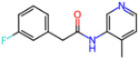	−16.5
	7GHR	1.655	OZC	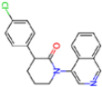	−13.7
	7GJD	1.79	QM9	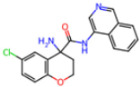	−16.7
	7GNN	1.81	S39	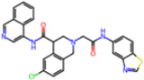	−18.0
	7GNQ	1.531	S5L	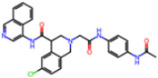	−22.8
	7US4	2.07	O69	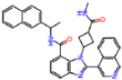	−21.6

**Table 5:** Contribution of the different modes of intermolecular interactions to the binding affinity between F2F-2020198-00X and its interacting residues in Mpro.

Catagory	Residue	Interaction Mode	${\Delta E}_{Int}^{aq}$ (kcal/mol)	Combined Energy (kcal/mol)
Non-bonded π-interactions	H41	CH–π	−2.7	−2.7
Hydrogen bonding	S144	HB	−2.1	
	E166	HB	−5.8	
	G143	HB	−1.5	−12.4
	H163	HB	−1.9	
	H164	HB	−1.1	

**Table 6:** Contribution of the different modes of intermolecular interactions to the binding affinity between EDG-MED-10fcb19e-1 and its interacting residues from Mpro.

Catagory	Residue	Interaction Mode	${\Delta E}_{Int}^{aq}$ (kcal/mol)	Combined Energy (kcal/mol)
Non-bonded π-interactions	H41	π–π	−2.4	
	M49	CH–π	−3.4	−12.6
	M165	CH–π	−3.4	
	N142	CH–π	−3.4	
Hydrogen bonding	S144	HB	−0.5	
	H163	HB	−2.8	−6.6
	E166	HB	−3.3	

**Table 7:** The average intermolecular interaction energies calculated at the B2PLYP/def2-QZVP level.

Covalent Inhibitors			
Residue	Interaction Mode	Occurrence	Average ${\Delta E}_{Int}^{aq}$ (kcal/mol)
E166	HB	10	−5.52
H163	HB	15	−2.29
H164	HB	10	−1.26
G143	HB	17	−1.22
S144	HB	18	−1.02
H41	HB	6	−2.1
N142	HB	1	−0.6
H41	π–π, CH–π	17	−2.98
N142	CH–π	3	−4.30
A191	CH–π	7	−2.26
M49	CH–π	1	−0.1
M165	CH–π	1	−5.2
E166	CH–π	1	−2.2
Q192	NH–π	1	−0.6
**Non-covalent Inhibitors**			
**Residue**	**Interaction Mode**	**Occurrence**	**Average** ${\Delta E}_{Int}^{aq}$ **(kcal/mol)**
E166	HB	17	−3.36
H163	HB	16	−2.55
S144	HB	16	−0.59
G143	HB	1	−0.2
H41	π–π, CH–π	19	−1.99
N142	CH–π	16	−2.96
M49	CH–π	15	−2.70
M165	CH–π	15	−3.71
E166	NH–π	1	−0.6

## Data Availability

The database of 963 Mpro inhibitors resulting from data mining of the PDB is provided in the Supplementary Materials.

## Supplementary Materials

The following supporting information can be downloaded at: https://www.mdpi.com/article/10.3390/molecules30102174/s1, Table S1: List of 963 Mpro inhibitors; Table S2: Intermolecular interaction energies between F2F-2020198-00X and its interacting residues from Mpro; Table S3: Intermolecular interaction energies between EDG-MED-10fcb19e-1 and its interacting residues from Mpro; Figure S1: Distribution of molecular descriptors for covalent inhibitors; Figure S2: Distribution of molecular descriptors for non-covalent inhibitors; Figure S3: Plot of average of weighted hydrogen bond count versus number of aromatic rings for a dataset of 1000 compounds randomly extracted from the PubChem library, augmented with a detailed description (Validation of WHBC Descriptor); Figure S4: Three-dimensional representations of distinct non-bonded interactions for representative covalent and non-covalent inhibitors. Refs. [59,60] are cited in the Supplementary Materials.
