# Supplementary material for: Molecular Recognition of SARS-CoV-2 Mpro Inhibitors: Insights from Cheminformatics and Quantum Chemistry
Source: Molecules. 2025 May 15;30(10):2174. doi: 10.3390/molecules30102174 (PMC12114402; doi:10.3390/molecules30102174)
Supplement: Supplementary file 1 [file molecules-30-02174-s001.zip › molecules-3597705-supplementary.pdf]

Table S1: List of MPro Inhibitors

348 Covalent Inhibitors			
PDB ID	Ligand ID	Ligand Name	Resolution (Å)
5RFF	T6M	1-{4-[(4-chlorophenyl)sulfonyl]piperazin-1-yl}ethan-1-one	1.78
5RFG	T6V	N-[(3S)-1,1-dioxo-2,3-dihydro-1H-1lambda~6~-thiophen-3-yl]-N-phenylacetamide	2.32
5RFH	T6Y	1-{4-[(5-chlorothiophen-2-yl)methyl]piperazin-1-yl}ethan-1-one	1.58
5RFI	T71	1-{4-[(2,5-dimethylphenyl)sulfonyl]piperazin-1-yl}ethan-1-one	1.69
5RFJ	T7A	N-(4-methoxy-1,3-benzothiazol-2-yl)acetamide	1.8
5RFK	T7D	N-(1-acetylpiperidin-4-yl)benzamide	1.75
5RFL	T7G	1-acetyl-N-(2-hydroxyphenyl)piperidine-4-carboxamide	1.64
5RFM	T7J	N-[(3R)-1,1-dioxo-2,3-dihydro-1H-1lambda~6~-thiophen-3-yl]-N-(4-methylphenyl)acetamide	2.06
5RFN	T7P	N-[(3R)-1,1-dioxo-2,3-dihydro-1H-1lambda~6~-thiophen-3-yl]-N-(4-fluorophenyl)acetamide	1.8
5RFO	T7S	1-[4-(piperidine-1-carbonyl)piperidin-1-yl]ethan-1-one	1.83
5RFP	T7V	N-[(1S)-1-(3-chlorophenyl)ethyl]acetamide	2.03
5RFQ	T7Y	N-[3-(2-oxopyrrolidin-1-yl)phenyl]acetamide	1.76
5RFR	T81	1-{4-[(5-bromothiophen-2-yl)methyl]piperazin-1-yl}ethan-1-one	1.71
5RFS	T84	1-{4-[(thiophen-3-yl)methyl]piperazin-1-yl}ethan-1-one	1.7
5RFT	T8A	1-[(4S)-4-phenyl-3,4-dihydroisoquinolin-2(1H)-yl]ethan-1-one	1.58
5RFU	T8D	1-{4-[(5-chlorothiophen-2-yl)sulfonyl]piperazin-1-yl}ethan-1-one	1.53
5RFV	T8J	1-[4-(thiophene-2-carbonyl)piperazin-1-yl]ethan-1-one	1.48
5RFW	T8M	1-{4-[(thiophen-2-yl)methyl]piperazin-1-yl}ethan-1-one	1.43
5RFX	T8P	1-[4-(4-methoxyphenyl)piperazin-1-yl]ethan-1-one	1.55
5RFY	T8S	1-acetyl-N-methyl-N-(propan-2-yl)piperidine-4-carboxamide	1.9
5RFZ	T8V	N-(2-chloropyridin-3-yl)acetamide	1.68
5RG0	T8Y	1,1'-(piperazine-1,4-diyl)di(ethan-1-one)	1.72
5RG2	T9M	N~2~-acetyl-N-prop-2-en-1-yl-D-allothreoninamide	1.63
5RG3	T9P	N~2~-acetyl-N~1~-prop-2-en-1-yl-L-aspartamide	1.58
5RGL	U0Y	1-[4-(4-methylbenzene-1-carbonyl)piperazin-1-yl]ethan-1-one	1.76
5RGM	U1D	N'-acetyl-4,5,6,7-tetrahydro-1-benzothiophene-2-carbohydrazide	2.04
5RGN	U1A	1-{4-[(4-methylphenyl)sulfonyl]piperazin-1-yl}ethan-1-one	1.86
5RGO	U1G	1-[4-(furan-2-carbonyl)piperazin-1-yl]ethan-1-one	1.74
5RGP	U1M	1-{4-[(2,4-dimethylphenyl)sulfonyl]piperazin-1-yl}ethan-1-one	2.07
5RGR	K1G	N,1-dimethyl-N-(propan-2-yl)-1H-pyrazolo[3,4-d]pyrimidin-4-amine	1.41
5RGT	UHS	N-[(1R)-2-(tert-butylamino)-2-oxo-1-(pyridin-3-yl)ethyl]-N-(5-tert-butyl-1,2-oxazol-3-yl)propanamide	2.22
5RH5	UHV	N-(5-tert-butyl-1,2-oxazol-3-yl)-N-[(1R)-2-[(4-methoxy-2-methylphenyl)amino]-2-oxo-1-(pyridin-3-yl)ethyl]propanamide	1.72
5RH6	UHY	N-[(1R)-2-[(2-ethyl-6-methylphenyl)amino]-2-oxo-1-(pyridin-3-yl)ethyl]-N-[6-(propan-2-yl)pyridin-3-yl]propanamide	1.6
5RH7	UJ1	N-(5-tert-butyl-1H-pyrazol-3-yl)-N-[(1R)-2-[(2-ethyl-6-methylphenyl)amino]-2-oxo-1-(pyridin-3-yl)ethyl]propanamide	1.71
5RH9	UJ4	N-{4-[(1S)-1-methoxyethyl]phenyl}-N-[(1R)-2-[(4-methoxy-2-methylphenyl)amino]-2-oxo-1-(pyridin-3-yl)ethyl]propanamide	1.91
5RHA	T8M	1-{4-[(thiophen-2-yl)methyl]piperazin-1-yl}ethan-1-one	1.51
5RHF	UPJ	1-acetyl-N-methyl-N-phenylpiperidine-4-carboxamide	1.76
5RL1	VEJ	N-(4-tert-butylphenyl)-N-[(1R)-2-[(3-methoxypropyl)amino]-2-oxo-1-(pyridin-3-yl)ethyl]propanamide	1.65
5RL2	VEM	N-(4-tert-butylphenyl)-N-[(1R)-2-[(2-methoxyethyl)amino]-2-oxo-1-(pyridin-3-yl)ethyl]propanamide	1.48

5RL3	VEP	N-(4-tert-butylphenyl)-N-[(1R)-2-[(oxan-4-yl)amino]-2-oxo-1-(pyridin-3-yl)ethyl]propanamide	1.51
5RL4	VEV	N-(4-tert-butylphenyl)-N-[(1R)-2-(methylamino)-2-oxo-1-(pyridin-3-yl)ethyl]propanamide	1.53
5RL5	VEY	N-(4-tert-butylphenyl)-N-[(1R)-2-(ethylamino)-2-oxo-1-(pyridin-3-yl)ethyl]propanamide	1.58
6WTJ	K36	(1S,2S)-2-({N-[(benzyloxy)carbonyl]-L-leucyl}amino)-1-hydroxy-3-[(3S)-2-oxopyrrolidin-3-yl]propane-1-sulfonic acid	1.9
6WTK	UED	N~2~-[(benzyloxy)carbonyl]-N-{(2S)-1-hydroxy-3-[(3S)-2-oxopyrrolidin-3-yl]propan-2-yl}-L-leucinamide	2
6WTT	K36	(1S,2S)-2-({N-[(benzyloxy)carbonyl]-L-leucyl}amino)-1-hydroxy-3-[(3S)-2-oxopyrrolidin-3-yl]propane-1-sulfonic acid	2.15
6WTT	B1S	(1R,2S)-2-({N-[(benzyloxy)carbonyl]-L-leucyl}amino)-1-hydroxy-3-[(3S)-2-oxopyrrolidin-3-yl]propane-1-sulfonic acid	2.15
6XHM	V2M	N-[(2S)-1-({(2S,3S)-3,4-dihydroxy-1-[(3S)-2-oxopyrrolidin-3-yl]butan-2-yl}amino)-4-methyl-1-oxopentan-2-yl]-4-methoxy-1H-indole-2-carboxamide	1.406
6XMK	QYS	(1S,2S)-2-[(N-{[(4,4-difluorocyclohexyl)methoxy]carbonyl}-L-leucyl)amino]-1-hydroxy-3-[(3S)-2-oxopyrrolidin-3-yl]propane-1-sulfonic acid	1.7
6XR3	V7G	N-[(2S)-1-({(1S,2S)-1-(1,3-benzothiazol-2-yl)-1-hydroxy-3-[(3S)-2-oxopyrrolidin-3-yl]propan-2-yl}amino)-4-methyl-1-oxopentan-2-yl]-4-methoxy-1H-indole-2-carboxamide	1.45
6Y2F	O6K	~{tert}-butyl ~{N}-[1-[(2~{S})-3-cyclopropyl-1-oxidanylidene-1-[[(2~{S},3~{R})-3-oxidanyl-4-oxidanylidene-1-[(3~{S})-2-oxidanylidenepyrrolidin-3-yl]-4-[(phenylmethyl)amino]butan-2-yl]amino]propan-2-yl]-2-oxidanylidene-pyridin-3-yl]carbamate	1.95
6Y2G	O6K	~{tert}-butyl ~{N}-[1-[(2~{S})-3-cyclopropyl-1-oxidanylidene-1-[[(2~{S},3~{R})-3-oxidanyl-4-oxidanylidene-1-[(3~{S})-2-oxidanylidenepyrrolidin-3-yl]-4-[(phenylmethyl)amino]butan-2-yl]amino]propan-2-yl]-2-oxidanylidene-pyridin-3-yl]carbamate	2.2
6YNQ	P6N	(2~{S})-2-methyl-3,4-dihydro-2~{H}-naphthalen-1-one	1.8
6Z2E	Q5T	(4~{S})-4-[[(2~{S})-2-[[(2~{S})-2-[[(2~{S})-2-[3-[2-[2-[2-[2-[5-[(3~{a}~{S},4~{R},6~{a}~{R})-2-oxidanylidene-3,3~{a},4,6~{a}-tetrahydro-1~{H}-thieno[3,4-d]imidazol-4-yl]pentanoylamino]ethoxy]ethoxy]ethoxy]ethoxy]propanoylamino]butanoyl]amino]-3,3-dimethyl-butanoyl]amino]-4-methyl-pentanoyl]amino]-6-methylsulfonyl-hexanamide	1.7
6ZRT	SV6	(1S,3aR,6aS)-2-[(2S)-2-({(2S)-2-cyclohexyl-2-[(pyrazin-2-ylcarbonyl)amino]acetyl}amino)-3,3-dimethylbutanoyl]-N-[(2R,3S)-1-(cyclopropylamino)-2-hydroxy-1-oxohexan-3-yl]octahydrocyclopenta[c]pyrrole-1-carboxamide	2.1
6ZRU	U5G	boceprevir (bound form)	2.1
7.00E+18	HUR	N-[(2S)-1-[[(2S)-1-(1,3-benzothiazol-2-yl)-1-oxidanylidene-3-[(3S)-2-oxidanylidenepyrrolidin-3-yl]propan-2-yl]amino]-4-methyl-1-oxidanylidene-pentan-2-yl]-4-methoxy-1H-indole-2-carboxamide	1.65
7.00E+19	HUO	(phenylmethyl) N-[(2S)-1-[[(2S)-1-[[(2S)-1-(1,3-benzothiazol-2-yl)-1-oxidanylidene-3-[(3S)-2-oxidanylidenepyrrolidin-3-yl]propan-2-yl]amino]-4-methyl-1-oxidanylidene-pentan-2-yl]amino]-3-methyl-1-oxidanylidene-butan-2-yl]carbamate	2.15
7ADW	R7Q	2-methyl-1-(4-methylphenyl)propan-1-one	1.63
7AEH	R8H	(2~{R})-5-oxidanylidene-~{N}-[(2~{R},3~{S})-3-oxidanyl-4-oxidanylidene-1-phenyl-4-(pyridin-2-ylmethylamino)butan-2-yl]-1-(phenylmethyl)pyrrolidine-2-carboxamide	1.3
7AHA	SIN	SUCCINIC ACID	1.68
7AK4	CB1	5-(AZIRIDIN-1-YL)-2,4-DINITROBENZAMIDE	1.63

7ARF	RVW	(2~{S},3~{R},4~{R},5~{S},6~{S})-2-(hydroxymethyl)-6-sulfanyl-oxane-3,4,5-triol	2
7AWS	S8E	5-[[(2-bromoethylamino)-(ethylamino)phosphoryl]oxymethyl]-1-methyl-~{N},~{N}-bis(oxidanyl)imidazol-2-amine	1.81
7AWU	S8B	~{N}-propan-2-yl-5-(2-pyridin-4-ylethynyl)pyridine-2-carboxamide	2.07
7AX6	S8H	(2~{S})-2-azanyl-5-oxidanylidene-5-[[(2~{S})-1-oxidanylidene-1-[(2-oxidanylidene-2-propan-2-yloxy-ethyl)amino]-3-sulfanyl-propan-2-yl]amino]pentanoic acid	1.95
7AY7	S8T	9-fluoranyl-3-propan-2-yl-5,6-dihydrobenzo[b][1]benzothiepine	1.55
7B3E	MYC	3,5,7-TRIHYDROXY-2-(3,4,5-TRIHYDROXYPHENYL)-4H-CHROMEN-4-ONE	1.77
7BE7	ALD	N-[(benzyloxy)carbonyl]-L-leucyl-N-[(2S)-1-hydroxy-4-methylpentan-2-yl]-L-leucinamide	1.68
7BFB	9JT	N-phenyl-2-selanylbenzamide	2.05
7BGP	ALD	N-[(benzyloxy)carbonyl]-L-leucyl-N-[(2S)-1-hydroxy-4-methylpentan-2-yl]-L-leucinamide	1.68
7BRP	U5G	boceprevir (bound form)	1.8
7C6S	U5G	boceprevir (bound form)	1.6
7C6U	K36	(1S,2S)-2-({N-[(benzyloxy)carbonyl]-L-leucyl}amino)-1-hydroxy-3-[(3S)-2-oxopyrrolidin-3-yl]propane-1-sulfonic acid	2
7C7P	SV6	(1S,3aR,6aS)-2-[(2S)-2-({(2S)-2-cyclohexyl-2-[(pyrazin-2-ylcarbonyl)amino]acetyl}amino)-3,3-dimethylbutanoyl]-N-[(2R,3S)-1-(cyclopropylamino)-2-hydroxy-1-oxohexan-3-yl]octahydrocyclopenta[c]pyrrole-1-carboxamide	1.74
7C8R	TG3	ethyl (4R)-4-[[(2S)-4-methyl-2-[[(2S,3R)-3-[(2-methylpropan-2-yl)oxy]-2-(phenylmethoxycarbonylamino)butanoyl]amino]pentanoyl]amino]-5-[(3S)-2-oxidanylidenepyrrolidin-3-yl]pentanoate	2.3
7C8T	NOL	N-[(BENZYLOXY)CARBONYL]-O-(TERT-BUTYL)-L-THREONYL-3-CYCLOHEXYL-N-[(1S)-2-HYDROXY-1-{[(3S)-2-OXOPYRROLIDIN-3-YL]METHYL}ETHYL]-L-ALANINAMIDE	2.05
7CB7	K36	(1S,2S)-2-({N-[(benzyloxy)carbonyl]-L-leucyl}amino)-1-hydroxy-3-[(3S)-2-oxopyrrolidin-3-yl]propane-1-sulfonic acid	1.69
7CBT	K36	(1S,2S)-2-({N-[(benzyloxy)carbonyl]-L-leucyl}amino)-1-hydroxy-3-[(3S)-2-oxopyrrolidin-3-yl]propane-1-sulfonic acid	2.346
7COM	U5G	boceprevir (bound form)	2.25
7CUU	ALD	N-[(benzyloxy)carbonyl]-L-leucyl-N-[(2S)-1-hydroxy-4-methylpentan-2-yl]-L-leucinamide	1.68
7CX9	GKF	3-iodanyl-1~{H}-indazole-7-carbaldehyde	1.73
7D1M	K36	(1S,2S)-2-({N-[(benzyloxy)carbonyl]-L-leucyl}amino)-1-hydroxy-3-[(3S)-2-oxopyrrolidin-3-yl]propane-1-sulfonic acid	1.35
7D1O	NNA	(1R,2S,5S)-3-[N-({1-[(tert-butylsulfonyl)methyl]cyclohexyl}carbamoyl)-3-methyl-L-valyl]-N-{(1S)-1-[(1R)-2-(cyclopropylamino)-1-hydroxy-2-oxoethyl]pentyl}-6,6-dimethyl-3-azabicyclo[3.1.0]hexane-2-carboxamide	1.78
7D3I	GQU	(3~{S},3~{a}~{S},6~{a}~{R})-2-[3-[3,5-bis(fluoranyl)phenyl]propanoyl]-~{N}-[(2~{S})-1-oxidanylidene-3-[(3~{S})-2-oxidanylidenepyrrolidin-3-yl]propan-2-yl]-3,3~{a},4,5,6,6~{a}-hexahydro-1~{H}-cyclopenta[c]pyrrole-3-carboxamide	2.004
7DGB	EOF	(2~{S})-4-methyl-~{N}-[(2~{S})-1-oxidanylidene-3-[(3~{S})-2-oxidanylidenepyrrolidin-3-yl]propan-2-yl]-2-[[(~{E})-3-phenylprop-2-enoyl]amino]pentanamide	1.678
7DGF	H60	(2~{S})-~{N}-[(2~{S})-1-oxidanylidene-3-[(3~{S})-2-oxidanylidenepiperidin-3-yl]propan-2-yl]-2-[[(~{E})-3-phenylprop-2-enoyl]amino]hexanamide	1.639

7DGG	H63	(2~{S})-~{N}-[(2~{S})-1-oxidanylidene-3-[(3~{S})-2-oxidanylidenepyrrolidin-3-yl]propan-2-yl]-2-[[(~{E})-3-phenylprop-2-enoyl]amino]hexanamide	2.004
7DGH	H6F	~{N}-[(2~{S})-3-methyl-1-[[(2~{S})-4-methyl-1-oxidanylidene-1-[[(2~{S})-1-oxidanylidene-3-[(3~{S})-2-oxidanylidenepiperidin-3-yl]propan-2-yl]amino]pentan-2-yl]amino]-1-oxidanylidene-butan-2-yl]naphthalene-2-carboxamide	1.968
7DGI	H6L	~{N}-[(2~{S})-3-methyl-1-[[(2~{S})-4-methyl-1-oxidanylidene-1-[[(2~{S})-1-oxidanylidene-3-[(3~{S})-2-oxidanylidenepiperidin-3-yl]propan-2-yl]amino]pentan-2-yl]amino]-1-oxidanylidene-butan-2-yl]-4-nitro-benzamide	1.898
7DHJ	H6R	(2~{S})-~{N}-[(2~{S})-1-oxidanylidene-3-[(3~{S})-2-oxidanylidenepyrrolidin-3-yl]propan-2-yl]-2-[[(~{E})-3-phenylprop-2-enoyl]amino]pent-4-ynamide	1.962
7DPP	MYC	3,5,7-TRIHYDROXY-2-(3,4,5-TRIHYDROXYPHENYL)-4H-CHROMEN-4-ONE	2.1
7DPU	HER	7-methoxy-3,5-bis(oxidanyl)-2-[3,4,5-tris(oxidanyl)phenyl]chromen-4-one	1.75
7DPV	HF0	(2S,3S)-3,5-dihydroxy-7-methoxy-2-(3,4,5-trihydroxyphenyl)chroman-4-one	2.35
7E6K	HYR	N-(2-phenoxyethyl)methanethioamide	1.6
7FAY	2XI	(2~{R})-~{N}-[(1~{R})-2-(~{tert}-butylamino)-2-oxidanylidene-1-pyridin-3-yl-ethyl]-~{N}-(4-~{tert}-butylphenyl)-2-oxidanyl-propanamide	2.1
7FAZ	2RI	(2~{R})-~{N}-dibenzofuran-3-yl-~{N}-[(1~{R})-2-[[(1~{S})-1-(4-fluorophenyl)ethyl]amino]-2-oxidanylidene-1-pyridin-3-yl-ethyl]-2-oxidanyl-propanamide	2.1
7GB1	KMF	N-[2-(4-acetylpiperazin-1-yl)ethyl]naphthalene-1-carboxamide	1.289
7GB2	KMX	1-{4-[(2-benzyl-1,3-thiazol-5-yl)methyl]piperazin-1-yl}ethan-1-one	1.5
7GB3	KNU	N-[(1S)-1-(3-chloro-5-fluorophenyl)ethyl]acetamide	1.379
7GBA	KS0	1-{4-[(4-fluorophenyl)methyl]piperazin-1-yl}propan-1-one	1.699
7GBB	KSI	1-[(3S)-4-[(3-chlorophenyl)methyl]-3-(2-methylpropyl)piperazin-1-yl]ethan-1-one	1.737
7GC5	L8F	N-(2-{7-[(4-acetylpiperazin-1-yl)methyl]-1H-indol-3-yl}ethyl)acetamide	1.57
7GC6	L8O	1-[(4R)-4-(3-methylphenyl)-3,4-dihydroisoquinolin-2(1H)-yl]ethan-1-one	1.69
7GCM	LQU	3-[(4R)-2-acetyl-1,2,3,4-tetrahydroisoquinolin-4-yl]benzonitrile	1.571
7GCP	LM0	(3S)-N',2-diacetyl-1,2,3,4-tetrahydroisoquinoline-3-carbohydrazide	1.56
7GCR	LRN	1-[(3R)-3-{(cyclohexylmethyl)[(1r,4R)-4-hydroxycyclohexyl]amino}piperidin-1-yl]ethan-1-one	1.67
7GCY	LW6	1H-indole-4-carbaldehyde	1.618
7GD9	LXF	N-{4-[(2-phenylethyl)sulfamoyl]-1,3-benzothiazol-2-yl}acetamide	1.621
7GDH	M5X	(3S)-3-hydroxy-2-oxo-2,3-dihydro-1H-indole-5-sulfonamide	1.891
7GDQ	MWN	methyl (3R)-5-bromo-3-hydroxy-2-oxo-2,3-dihydro-1H-indole-7-carboxylate	1.721
7GDR	MX9	(3-methylphenyl)methyl (3R)-3-hydroxy-2-oxo-2,3-dihydro-1H-indole-7-carboxylate	1.661
7GDS	MZF	(3R)-5-bromo-3-hydroxy-1-[(1,2,4-oxadiazol-3-yl)methyl]-1,3-dihydro-2H-indol-2-one	1.46
7GDT	N00	(2R)-2-(6-chloro-1-methyl-9H-carbazol-2-yl)propanoic acid	1.509
7GDX	N1U	(3S)-5-bromo-1-[(3,4-dimethoxyphenyl)methyl]-3-hydroxy-7-methyl-1,3-dihydro-2H-indol-2-one	1.839
7GDY	N2X	(3S)-5-bromo-1-[(2-ethoxyphenyl)methyl]-3-hydroxy-1,3-dihydro-2H-indol-2-one	1.611

7GF7	O3U	N-[(1R)-1,5-dicyano-4-(methylsulfanyl)-3-azaspiro[5.5]undeca-2,4-dien-2-yl]acetamide	1.941
7GF8	O4L	N-(3-acetyl-2,5-dimethyl-1H-pyrrol-1-yl)-4-oxo-3,4-dihydrophthalazine-1-carboxamide	1.43
7GF9	O5C	1-[(4S)-3-(4-fluorobenzoyl)-2-methylindolizin-1-yl]ethan-1-one	1.97
7GFV	OJO	1-{4-[(3-chloro-5-hydroxyphenyl)methyl]piperazin-1-yl}ethan-1-one	1.73
7GFW	OK9	N-{(1Z)-1-[5-(morpholin-4-yl)thiophen-2-yl]-3-oxoprop-1-en-2-yl}thiophene-2-carboxamide	1.569
7GFY	OLX	[(3R)-5-ethyl-3-hydroxy-2-oxo-2,3-dihydro-1H-indol-1-yl]acetic acid	1.55
7GG3	OOL	(E)-1-(4,6-dimethoxypyrimidin-2-yl)methanimine	1.42
7GH2	Q0I	(4R)-6-chloro-N-(isoquinolin-4-yl)-N-propanoyl-3,4-dihydro-2H-1-benzopyran-4-carboxamide	1.62
7GHA	Q4F	1-{(1S,4S)-5-[(3-chlorophenyl)methyl]-2,5-diazabicyclo[2.2.1]heptan-2-yl}ethan-1-one	1.72
7GHG	Q69	N-[(1R)-1-(3-chlorophenyl)-2-hydroxyethyl]acetamide	1.45
7GHH	Q6U	1-[4-(prop-2-yn-1-yl)piperazin-1-yl]ethan-1-one	1.56
7GHI	Q7C	N~3~-acetyl-N~3~-[(3S)-1,1-dioxo-1lambda~6~-thiolan-3-yl]-beta-alaninamide	1.65
7GHJ	Q7R	1-[4-(diphenylmethyl)piperazin-1-yl]ethan-1-one	1.526
7GI3	Q0I	(4R)-6-chloro-N-(isoquinolin-4-yl)-N-propanoyl-3,4-dihydro-2H-1-benzopyran-4-carboxamide	2.1
7GJ7	Q0I	(4R)-6-chloro-N-(isoquinolin-4-yl)-N-propanoyl-3,4-dihydro-2H-1-benzopyran-4-carboxamide	1.88
7GJE	QML	(3S)-5-chloro-N-(isoquinolin-4-yl)-N-propanoyl-2,3-dihydro-1-benzofuran-3-carboxamide	2.03
7JKV	V7G	N-[(2S)-1-({(1S,2S)-1-(1,3-benzothiazol-2-yl)-1-hydroxy-3-[(3S)-2-oxopyrrolidin-3-yl]propan-2-yl}amino)-4-methyl-1-oxopentan-2-yl]-4-methoxy-1H-indole-2-carboxamide	1.25
7JP0	VJA	N-[(benzyloxy)carbonyl]-L-valyl-N-{(2R)-1-hydroxy-3-[(3S)-2-oxopyrrolidin-3-yl]propan-2-yl}-L-leucinamide	1.65
7JPZ	GHX	(phenylmethyl) N-[(2S)-1-oxidanylidene-1-[[(2S)-1-oxidanyl-3-[(3S)-2-oxidanylidenepyrrolidin-3-yl]propan-2-yl]amino]-3-phenyl-propan-2-yl]carbamate	1.6
7JQ0	VHV	N-[(benzyloxy)carbonyl]-L-valyl-N-{(2S)-1-hydroxy-3-[(3S)-2-oxopyrrolidin-3-yl]propan-2-yl}-L-leucinamide	1.65
7JQ1	VHJ	N-[(benzyloxy)carbonyl]-L-valyl-N-{(2S)-1-hydroxy-3-[(3R)-2-oxo-3,4-dihydro-2H-pyrrol-3-yl]propan-2-yl}-L-phenylalaninamide	1.65
7JQ2	VHM	N-[(benzyloxy)carbonyl]-L-valyl-3-cyclohexyl-N-{(2S)-1-hydroxy-3-[(3S)-2-oxopyrrolidin-3-yl]propan-2-yl}-L-alaninamide	1.4
7JQ3	VHP	N-[(benzyloxy)carbonyl]-O-tert-butyl-L-threonyl-N-{(2S)-1-hydroxy-3-[(3S)-2-oxopyrrolidin-3-yl]propan-2-yl}-L-leucinamide	2.1
7JQ4	XM2	N-[(benzyloxy)carbonyl]-O-tert-butyl-L-threonyl-N-{(2S)-1-hydroxy-3-[(3S)-2-oxopyrrolidin-3-yl]propan-2-yl}-L-phenylalaninamide	1.65
7JQ5	NOL	N-[(BENZYLOXY)CARBONYL]-O-(TERT-BUTYL)-L-THREONYL-3-CYCLOHEXYL-N-[(1S)-2-HYDROXY-1-{[(3S)-2-OXOPYRROLIDIN-3-YL]METHYL}ETHYL]-L-ALANINAMIDE	1.9
7JSU	UED	N~2~-[(benzyloxy)carbonyl]-N-{(2S)-1-hydroxy-3-[(3S)-2-oxopyrrolidin-3-yl]propan-2-yl}-L-leucinamide	1.83
7JT0	LW1	thiophene-2-carbaldehyde	1.73
7JT7	TG3	ethyl (4R)-4-[[(2S)-4-methyl-2-[[(2S,3R)-3-[(2-methylpropan-2-yl)oxy]-2-(phenylmethoxycarbonylamino)butanoyl]amino]pentanoyl]amino]-5-[(3S)-2-oxidanylidenepyrrolidin-3-yl]pentanoate	1.94
7JW8	TG3	ethyl (4R)-4-[[(2S)-4-methyl-2-[[(2S,3R)-3-[(2-methylpropan-2-yl)oxy]-2-	1.84

		(phenylmethoxycarbonylamino)butanoyl]amino]pentanoyl]amino]-5-[(3S)-2-oxidanylidenepyrrolidin-3-yl]pentanoate	
7JYC	NNA	(1R,2S,5S)-3-[N-({1-[(tert-butylsulfonyl)methyl]cyclohexyl}carbamoyl)-3-methyl-L-valyl]-N-{(1S)-1-[(1R)-2-(cyclopropylamino)-1-hydroxy-2-oxoethyl]pentyl}-6,6-dimethyl-3-azabicyclo[3.1.0]hexane-2-carboxamide	1.79
7K0E	K36	(1S,2S)-2-({N-[(benzyloxy)carbonyl]-L-leucyl}amino)-1-hydroxy-3-[(3S)-2-oxopyrrolidin-3-yl]propane-1-sulfonic acid	1.9
7K0F	VR4	N-{(2S,3R)-4-(benzylamino)-3-hydroxy-4-oxo-1-[(3S)-2-oxopyrrolidin-3-yl]butan-2-yl}-N~2~-[(benzyloxy)carbonyl]-L-leucinamide	1.65
7K40	U5G	boceprevir (bound form)	1.35
7K6D	SV6	(1S,3aR,6aS)-2-[(2S)-2-({(2S)-2-cyclohexyl-2-[(pyrazin-2-ylcarbonyl)amino]acetyl}amino)-3,3-dimethylbutanoyl]-N-[(2R,3S)-1-(cyclopropylamino)-2-hydroxy-1-oxohexan-3-yl]octahydrocyclopenta[c]pyrrole-1-carboxamide	1.48
7K6E	SV6	(1S,3aR,6aS)-2-[(2S)-2-({(2S)-2-cyclohexyl-2-[(pyrazin-2-ylcarbonyl)amino]acetyl}amino)-3,3-dimethylbutanoyl]-N-[(2R,3S)-1-(cyclopropylamino)-2-hydroxy-1-oxohexan-3-yl]octahydrocyclopenta[c]pyrrole-1-carboxamide	1.63
7KYU	XC4	1-[(1H-indole-5-carbonyl)oxy]-1H-benzotriazole	1.48
7L8I	AG7	4-{2-(4-FLUORO-BENZYL)-6-METHYL-5-[(5-METHYL-ISOXAZOLE-3-CARBONYL)-AMINO]-4-OXO-HEPTANOYLAMINO}-5-(2-OXO-PYRROLIDIN-3-YL)-PENTANOIC ACID ETHYL ESTER	2.1
7L8J	AG7	4-{2-(4-FLUORO-BENZYL)-6-METHYL-5-[(5-METHYL-ISOXAZOLE-3-CARBONYL)-AMINO]-4-OXO-HEPTANOYLAMINO}-5-(2-OXO-PYRROLIDIN-3-YL)-PENTANOIC ACID ETHYL ESTER	2.45
7LCO	XTJ	(3-fluorophenyl)methyl [(2S)-3-cyclopropyl-1-oxo-1-({(2S)-1-oxo-3-[(3S)-2-oxopyrrolidin-3-yl]propan-2-yl}amino)propan-2-yl]carbamate	1.9
7LCR	XTM	N~2~-{[(3-fluorophenyl)methoxy]carbonyl}-N-{(2S)-1-hydroxy-3-[(3S)-2-oxopyrrolidin-3-yl]propan-2-yl}-L-leucinamide	1.95
7LCS	XTP	benzyl [(2S)-3-cyclopropyl-1-({(2S)-1-hydroxy-3-[(3S)-2-oxopyrrolidin-3-yl]propan-2-yl}amino)-1-oxopropan-2-yl]carbamate	1.85
7LDL	XV4	N-[(2S)-1-({(2S)-1-hydroxy-3-[(3S)-2-oxopyrrolidin-3-yl]propan-2-yl}amino)-4-methyl-1-oxopentan-2-yl]-4-methoxy-1H-indole-2-carboxamide	2
7LTN	YCV	2-[4-(1~{H}-indazol-4-yl)-2-methanoyl-6-methoxy-phenoxy]-~{N},~{N}-dimethyl-ethanamide	1.79
7LYH	YHJ	benzyl (1S,3aR,6aS)-1-({(2S)-1-hydroxy-3-[(3S)-2-oxopyrrolidin-3-yl]propan-2-yl}carbamoyl)hexahydrocyclopenta[c]pyrrole-2(1H)-carboxylate	1.9
7LYI	YHI	benzyl (1R,2S,5S)-2-({(2S)-1-hydroxy-3-[(3S)-2-oxopyrrolidin-3-yl]propan-2-yl}carbamoyl)-6,6-dimethyl-3-azabicyclo[3.1.0]hexane-3-carboxylate	1.9
7MAT	H37	D-phenylalanyl-N-[(3S)-6-carbamimidamido-1-chloro-2-oxohexan-3-yl]-L-phenylalaninamide	2.74
7MAU	YVP	N-{3-[(prop-2-yn-1-yl)oxy]propanoyl}-D-phenylalanyl-N-[(3S)-6-carbamimidamido-2-oxohexan-3-yl]-L-phenylalaninamide	1.95
7MAV	YVY	N-{3-[(prop-2-yn-1-yl)oxy]propanoyl}-D-phenylalanyl-N-[(3S)-6-(carbamoylamino)-2-oxohexan-3-yl]-L-phenylalaninamide	1.91
7MAW	YVA	ethyl (4R)-4-({3-cyclopropyl-N-[(2E)-3-(4-ethynylphenyl)prop-2-enoyl]-L-alanyl}amino)-5-[(3S)-2-oxopyrrolidin-3-yl]pentanoate	2.07
7MAX	YV7	D-phenylalanyl-N-{(2R)-5-ethoxy-5-oxo-1-[(3S)-2-oxopyrrolidin-3-yl]pentan-2-yl}-4-fluoro-L-phenylalaninamide	1.98

7MAZ	YVD	4-fluoro-D-phenylalanyl-N-{(2R)-5-ethoxy-5-oxo-1-[(3S)-2-oxopyrrolidin-3-yl]pentan-2-yl}-4-fluoro-L-phenylalaninamide	1.7
7MB0	YVG	D-phenylalanyl-N-{(2R)-5-ethoxy-5-oxo-1-[(3S)-2-oxopyrrolidin-3-yl]pentan-2-yl}-L-phenylalaninamide	1.54
7MB1	YVJ	4-fluoro-D-phenylalanyl-N-{(2R)-5-ethoxy-5-oxo-1-[(3S)-2-oxopyrrolidin-3-yl]pentan-2-yl}-L-phenylalaninamide	1.43
7MB2	YVM	4-fluoro-N-{3-[(prop-2-yn-1-yl)oxy]propanoyl}-D-phenylalanyl-N-{(2R)-5-ethoxy-5-oxo-1-[(3S)-2-oxopyrrolidin-3-yl]pentan-2-yl}-L-phenylalaninamide	1.89
7MB3	YVV	N-{3-[(prop-2-yn-1-yl)oxy]propanoyl}-D-phenylalanyl-N-{(2R)-5-ethoxy-5-oxo-1-[(3S)-2-oxopyrrolidin-3-yl]pentan-2-yl}-L-phenylalaninamide	1.81
7MBI	YWJ	4-methoxy-N-[(2S)-4-methyl-1-oxo-1-({(2S)-3-oxo-1-[(3S)-2-oxopiperidin-3-yl]butan-2-yl}amino)pentan-2-yl]-1H-indole-2-carboxamide	2.15
7MLF	C7A	N-(4-tert-butylphenyl)-2-chloro-N-[(1R)-2-(cyclohexylamino)-2-oxo-1-(pyridin-3-yl)ethyl]acetamide	2.6
7MLG	ZJ1	(2R)-2-[(4-tert-butylphenyl)(ethanesulfonyl)amino]-N-cyclohexyl-2-(pyridin-3-yl)acetamide	2.5
7NBR	U5G	boceprevir (bound form)	2.4
7NBS	SV6	(1S,3aR,6aS)-2-[(2S)-2-({(2S)-2-cyclohexyl-2-[(pyrazin-2-ylcarbonyl)amino]acetyl}amino)-3,3-dimethylbutanoyl]-N-[(2R,3S)-1-(cyclopropylamino)-2-hydroxy-1-oxohexan-3-yl]octahydrocyclopenta[c]pyrrole-1-carboxamide	1.7
7NBY	U88	5-nitro-1,3-thiazole	1.93
7NF5	ALD	N-[(benzyloxy)carbonyl]-L-leucyl-N-[(2S)-1-hydroxy-4-methylpentan-2-yl]-L-leucinamide	1.94
7NG3	ALD	N-[(benzyloxy)carbonyl]-L-leucyl-N-[(2S)-1-hydroxy-4-methylpentan-2-yl]-L-leucinamide	1.8
7NG6	ALD	N-[(benzyloxy)carbonyl]-L-leucyl-N-[(2S)-1-hydroxy-4-methylpentan-2-yl]-L-leucinamide	1.87
7NT1	UQW	[(2R)-1-[2-(1H-indol-3-yl)ethylamino]-1-oxidanylidene-butan-2-yl] prop-2-enoate	2.85
7NT2	URK	[(1S)-2-[(2,3-dimethoxyphenyl)methylamino]-1-(4-nitrophenyl)-2-oxidanylidene-ethyl] prop-2-enoate	2.145
7NTQ	35J	N-(pyridin-3-ylmethyl)thioformamide	1.495
7NTV	US8	2-acetamido-N-cyclopropyl-5-phenyl-thiophene-3-carboxamide	2.065
7NUK	USH	2-[2-chloranylethanoyl(propyl)amino]-~{N}-(2-methoxyphenyl)ethanamide	2.19
7NW2	USZ	~{N}-(4-~{tert}-butylphenyl)-~{N}-[(1~{R})-2-[2-(3-fluorophenyl)ethylamino]-2-oxidanylidene-1-pyridin-3-yl-ethyl]propanamide	2.1
7P35	AG7	4-{2-(4-FLUORO-BENZYL)-6-METHYL-5-[(5-METHYL-ISOXAZOLE-3-CARBONYL)-AMINO]-4-OXO-HEPTANOYLAMINO}-5-(2-OXO-PYRROLIDIN-3-YL)-PENTANOIC ACID ETHYL ESTER	2.256
7P51	5P9	N-(5-chloropyridin-2-yl)-3-oxo-2,3-dihydro-1H-indene-1-carboxamide	1.474
7Q5E	90I	benzyl (S)-2-(((S)-3,4-dioxo-1-((S)-2-oxopyrrolidin-3-yl)-4-(phenethylamino)butan-2-yl)carbamoyl)pyrrolidine-1-carboxylate	1.67
7Q5F	90X	(S)-1-(2-(2,4-dichlorophenoxy)acetyl)-N-((S)-3,4-dioxo-1-((S)-2-oxopyrrolidin-3-yl)-4-(phenethylamino)butan-2-yl)pyrrolidine-2-carboxamide	1.72
7QKA	UED	N~2~-[(benzyloxy)carbonyl]-N-{(2S)-1-hydroxy-3-[(3S)-2-oxopyrrolidin-3-yl]propan-2-yl}-L-leucinamide	1.8

7QL8	I70	(1R,2S,5S)-N-{(2S,3R)-4-amino-3-hydroxy-4-oxo-1-[(3S)-2-oxopyrrolidin-3-yl]butan-2-yl}-3-[N-(tert-butylcarbamoyl)-3-methyl-L-valyl]-6,6-dimethyl-3-azabicyclo[3.1.0]hexane-2-carboxamide	1.807
7QT7	UHV	N-(5-tert-butyl-1,2-oxazol-3-yl)-N-[(1R)-2-[(4-methoxy-2-methylphenyl)amino]-2-oxo-1-(pyridin-3-yl)ethyl]propanamide	2.25
7QT8	R8H	(2~{R})-5-oxidanylidene-~{N}-[(2~{R},3~{S})-3-oxidanyl-4-oxidanylidene-1-phenyl-4-(pyridin-2-ylmethylamino)butan-2-yl]-1-(phenylmethyl)pyrrolidine-2-carboxamide	2.01
7QT9	UJ1	N-(5-tert-butyl-1H-pyrazol-3-yl)-N-[(1R)-2-[(2-ethyl-6-methylphenyl)amino]-2-oxo-1-(pyridin-3-yl)ethyl]propanamide	2.43
7R7H	4IT	N-[(2S)-1-({(1E,2S)-1-imino-3-[(3S)-2-oxopyrrolidin-3-yl]propan-2-yl}amino)-4-methyl-1-oxopentan-2-yl]-4-methoxy-1H-indole-2-carboxamide	2.15
7RBZ	4IJ	5-chloropyridin-3-yl 2,3-dihydro-1H-indole-4-carboxylate	1.65
7RC0	4I9	5-chloro-4-methylpyridin-3-yl 1H-indole-4-carboxylate	1.65
7RFR	4W8	(1R,2S,5S)-N-{(1S,2S)-1-(1,3-benzothiazol-2-yl)-1-hydroxy-3-[(3S)-2-oxopyrrolidin-3-yl]propan-2-yl}-3-(4-methoxy-1H-indole-2-carbonyl)-6,6-dimethyl-3-azabicyclo[3.1.0]hexane-2-carboxamide	1.626
7RFS	4WI	(1R,2S,5S)-N-{(1E,2S)-1-imino-3-[(3S)-2-oxopyrrolidin-3-yl]propan-2-yl}-6,6-dimethyl-3-[3-methyl-N-(trifluoroacetyl)-L-valyl]-3-azabicyclo[3.1.0]hexane-2-carboxamide	1.91
7RFU	4YG	(1R,2S,5S)-N-{(1S,2S)-1-(1,3-benzothiazol-2-yl)-1-hydroxy-3-[(3S)-2-oxopyrrolidin-3-yl]propan-2-yl}-3-[N-(methanesulfonyl)-L-valyl]-6,6-dimethyl-3-azabicyclo[3.1.0]hexane-2-carboxamide	2.498
7RFW	4WI	(1R,2S,5S)-N-{(1E,2S)-1-imino-3-[(3S)-2-oxopyrrolidin-3-yl]propan-2-yl}-6,6-dimethyl-3-[3-methyl-N-(trifluoroacetyl)-L-valyl]-3-azabicyclo[3.1.0]hexane-2-carboxamide	1.729
7RN0	5ZB	(2R)-2-{acetyl[4-(1H-pyrrol-1-yl)phenyl]amino}-N-[(1S)-1-phenylethyl]-2-(pyridin-3-yl)acetamide	2.25
7RN1	5ZF	N-([1,1'-biphenyl]-4-yl)-2-chloro-N-[(1R)-2-oxo-2-{[(1S)-1-phenylethyl]amino}-1-(pyridin-3-yl)ethyl]acetamide	2.3
7RVN	7VB	N-[(benzyloxy)carbonyl]-L-valyl-N-{(2S)-1-hydroxy-3-[(3S)-2-oxopyrrolidin-3-yl]propan-2-yl}-4-methylidene-L-norvalinamide	1.63
7RVQ	7VW	N-[(benzyloxy)carbonyl]-O-tert-butyl-L-threonyl-N-{(2S)-1-hydroxy-3-[(3S)-2-oxopyrrolidin-3-yl]propan-2-yl}-4-methyl-L-leucinamide	2.48
7RVS	81L	N-[(benzyloxy)carbonyl]-3-methyl-L-valyl-3-cyclopropyl-N-{(2S)-1-hydroxy-3-[(3S)-2-oxopyrrolidin-3-yl]propan-2-yl}-L-alaninamide	1.85
7RVX	7YI	benzyl [(1S)-1-cyclopropyl-2-{[(2S)-3-cyclopropyl-1-({(2S)-1-hydroxy-3-[(3S)-2-oxopyrrolidin-3-yl]propan-2-yl}amino)-1-oxopropan-2-yl]amino}-2-oxoethyl]carbamate	1.85
7S6W	8G9	(1R,2S,5S)-3-[N-(tert-butylcarbamoyl)-3-methyl-L-valyl]-N-{(2S)-1-hydroxy-3-[(3S)-2-oxopyrrolidin-3-yl]propan-2-yl}-6,6-dimethyl-3-azabicyclo[3.1.0]hexane-2-carboxamide	2.29
7SET	I70	(1R,2S,5S)-N-{(2S,3R)-4-amino-3-hydroxy-4-oxo-1-[(3S)-2-oxopyrrolidin-3-yl]butan-2-yl}-3-[N-(tert-butylcarbamoyl)-3-methyl-L-valyl]-6,6-dimethyl-3-azabicyclo[3.1.0]hexane-2-carboxamide	1.7
7SF1	8ZI	(1R,2S,5S)-N-{(2S,3R)-4-amino-3-hydroxy-4-oxo-1-[(3S)-2-oxopyrrolidin-3-yl]butan-2-yl}-3-[N-(3,3-dimethylbutanoyl)-3-methyl-L-valyl]-6,6-dimethyl-3-azabicyclo[3.1.0]hexane-2-carboxamide	1.85
7SF3	90H	(1R,2S,5S)-N-{(2S,3R)-3-hydroxy-4-(methylamino)-4-oxo-1-[(3S)-2-oxopyrrolidin-3-yl]butan-2-yl}-6,6-dimethyl-3-[3-methyl-N-(trifluoroacetyl)-L-valyl]-3-azabicyclo[3.1.0]hexane-2-carboxamide	1.75
7SFB	90U	benzyl (1R,2S,5S)-2-({(2S,3R)-4-amino-3-hydroxy-4-oxo-1-[(3S)-2-oxopyrrolidin-3-yl]butan-2-yl}carbamoyl)-6,6-dimethyl-3-azabicyclo[3.1.0]hexane-3-carboxylate	1.9

7SFH	91I	(1R,2S,5S)-N-{(2S,3R)-4-amino-3-hydroxy-4-oxo-1-[(3S)-2-oxopyrrolidin-3-yl]butan-2-yl}-6,6-dimethyl-3-(3-phenylpropanoyl)-3-azabicyclo[3.1.0]hexane-2-carboxamide	1.4
7SFI	91Z	(1R,2S,5S)-N-{(2S,3R)-4-amino-3-hydroxy-4-oxo-1-[(3S)-2-oxopyrrolidin-3-yl]butan-2-yl}-6,6-dimethyl-3-[N-(2,4,6-trifluorophenyl)glycyl]-3-azabicyclo[3.1.0]hexane-2-carboxamide	1.95
7SGH	99W	(S)-N-((S)-1-imino-3-((S)-2-oxopyrrolidin-3-yl)propan-2-yl)-4-methyl-2-(2-((2,4,6-trifluorophenyl)amino)acetamido)pentanamide	1.85
7SH7	9GI	benzyl [(2S,3R)-3-tert-butoxy-1-{[(2S)-3-cyclohexyl-1-oxo-1-(2-{[(3S)-2-oxopyrrolidin-3-yl]methyl}-2-propanoylhydrazinyl)propan-2-yl]amino}-1-oxobutan-2-yl]carbamate (non-preferred name)	1.85
7SH8	GJ3	benzyl [(2S,3R)-1-{[(2S)-1-(2-acetyl-2-{[(3S)-2-oxopyrrolidin-3-yl]methyl}hydrazinyl)-3-cyclohexyl-1-oxopropan-2-yl]amino}-3-tert-butoxy-1-oxobutan-2-yl]carbamate (non-preferred name)	1.8
7SH9	9HA	benzyl [(2S,3R)-1-({(2S)-1-[2-acetyl-2-(3-amino-3-oxopropyl)hydrazinyl]-3-cyclohexyl-1-oxopropan-2-yl}amino)-3-tert-butoxy-1-oxobutan-2-yl]carbamate (non-preferred name)	1.85
7SI9	4WI	(1R,2S,5S)-N-{(1E,2S)-1-imino-3-[(3S)-2-oxopyrrolidin-3-yl]propan-2-yl}-6,6-dimethyl-3-[3-methyl-N-(trifluoroacetyl)-L-valyl]-3-azabicyclo[3.1.0]hexane-2-carboxamide	2
7T45	EW9	(1S,2S)-2-{[N-({[7-(tert-butoxycarbonyl)-7-azaspiro[3.5]nonan-2-yl]oxy}carbonyl)-L-leucyl]amino}-1-hydroxy-3-[(3S)-2-oxopyrrolidin-3-yl]propane-1-sulfonic acid	1.65
7T46	F8C	(1S,2S)-1-hydroxy-2-{[N-({[7-(2-methylpropanoyl)-7-azaspiro[3.5]nonan-2-yl]oxy}carbonyl)-L-leucyl]amino}-3-[(3S)-2-oxopyrrolidin-3-yl]propane-1-sulfonic acid	1.45
7T46	F5L	(1R,2S)-1-hydroxy-2-{[N-({[7-(2-methylpropanoyl)-7-azaspiro[3.5]nonan-2-yl]oxy}carbonyl)-L-leucyl]amino}-3-[(3S)-2-oxopyrrolidin-3-yl]propane-1-sulfonic acid	1.45
7T48	FHS	(1S,2S)-1-hydroxy-3-[(3S)-2-oxopyrrolidin-3-yl]-2-{[N-({[7-(phenylacetyl)-7-azaspiro[3.5]nonan-2-yl]oxy}carbonyl)-L-leucyl]amino}propane-1-sulfonic acid	1.9
7T48	FEY	(1R,2S)-2-{[N-({[(2r,4R)-7-acetyl-7-azaspiro[3.5]non-5-en-2-yl]oxy}carbonyl)-L-leucyl]amino}-1-hydroxy-3-[(3S)-2-oxopyrrolidin-3-yl]propane-1-sulfonic acid	1.9
7T49	FV5	(1R,2S)-1-hydroxy-2-{[N-({[7-(methanesulfonyl)-7-azaspiro[3.5]nonan-2-yl]oxy}carbonyl)-L-leucyl]amino}-3-[(3S)-2-oxopyrrolidin-3-yl]propane-1-sulfonic acid	1.75
7T49	FVE	(1S,2S)-1-hydroxy-2-{[N-({[7-(methanesulfonyl)-7-azaspiro[3.5]nonan-2-yl]oxy}carbonyl)-L-leucyl]amino}-3-[(3S)-2-oxopyrrolidin-3-yl]propane-1-sulfonic acid	1.75
7T4B	FWI	(1R,2S)-2-{[N-({[1-(tert-butoxycarbonyl)azetidin-3-yl]methoxy}carbonyl)-L-leucyl]amino}-1-hydroxy-3-[(3R)-2-oxo-2,3-dihydro-1H-pyrrol-3-yl]propane-1-sulfonic acid	1.6
7T4B	FZI	(1S,2S)-2-{[N-({[1-(tert-butoxycarbonyl)azetidin-3-yl]methoxy}carbonyl)-L-leucyl]amino}-1-hydroxy-3-[(3R)-2-oxo-2,3-dihydro-1H-pyrrol-3-yl]propane-1-sulfonic acid	1.6
7TDU	I1W	(1R,2S,5S)-N-{(1S,2S)-1-(1,3-benzothiazol-2-yl)-1-hydroxy-3-[(3S)-2-oxo(1-~2~H)pyrrolidin-3-yl]propan-2-yl}-3-{N-[tert-butyl(~2~H)carbamoyl]-3-methyl-L-(N-~2~H)valyl}-6,6-dimethyl-3-azabicyclo[3.1.0]hexane-2-(~2~H)carboxamide	1.85, 2.2
7TE0	4WI	(1R,2S,5S)-N-{(1E,2S)-1-imino-3-[(3S)-2-oxopyrrolidin-3-yl]propan-2-yl}-6,6-dimethyl-3-[3-methyl-N-(trifluoroacetyl)-L-valyl]-3-azabicyclo[3.1.0]hexane-2-carboxamide	2

7TEH	I1Z	(1R,2S,5S)-3-[N-(tert-butylcarbamoyl)-3-methyl-L-valyl]-N-{(1Z,2S)-1-imino-3-[(3S)-2-oxopyrrolidin-3-yl]propan-2-yl}-6,6-dimethyl-3-azabicyclo[3.1.0]hexane-2-carboxamide	1.8
7TFR	NB2	(1R,2S,5S)-N-{(1Z,2S)-1-imino-3-[(3S)-2-oxopyrrolidin-3-yl]propan-2-yl}-6,6-dimethyl-3-[3-methyl-N-({1-[(2-methylpropane-2-sulfonyl)methyl]cyclohexyl}carbamoyl)-L-valyl]-3-azabicyclo[3.1.0]hexane-2-carboxamide	1.8
7TGR	K36	(1S,2S)-2-({N-[(benzyloxy)carbonyl]-L-leucyl}amino)-1-hydroxy-3-[(3S)-2-oxopyrrolidin-3-yl]propane-1-sulfonic acid	1.68
7TGR	B1S	(1R,2S)-2-({N-[(benzyloxy)carbonyl]-L-leucyl}amino)-1-hydroxy-3-[(3S)-2-oxopyrrolidin-3-yl]propane-1-sulfonic acid	1.68
7TIA	XTP	benzyl [(2S)-3-cyclopropyl-1-({(2S)-1-hydroxy-3-[(3S)-2-oxopyrrolidin-3-yl]propan-2-yl}amino)-1-oxopropan-2-yl]carbamate	1.64
7TIU	V46	(1S,2S)-2-[(N-{[(3-chlorophenyl)methoxy]carbonyl}-L-leucyl)amino]-1-hydroxy-3-[(3S)-2-oxopyrrolidin-3-yl]propane-1-sulfonic acid	1.65
7TIV	W48	(1S,2S)-2-[(N-{[(3-chlorophenyl)methoxy]carbonyl}-3-cyclohexyl-L-alanyl)amino]-1-hydroxy-3-[(3R)-2-oxo-2,3-dihydro-1H-pyrrol-3-yl]propane-1-sulfonic acid	2.08
7TIW	I54	(1S,2S)-2-[(N-{[(2-chlorophenyl)methoxy]carbonyl}-L-leucyl)amino]-1-hydroxy-3-[(3S)-2-oxopyrrolidin-3-yl]propane-1-sulfonic acid	1.68
7TIX	Q56	N~2~-{[(naphthalen-2-yl)methoxy]carbonyl}-N-{(2S)-1-oxo-3-[(3S)-2-oxopyrrolidin-3-yl]propan-2-yl}-L-leucinamide	2
7TIY	Y48	(1S,2S)-1-hydroxy-3-[(3S)-2-oxopyrrolidin-3-yl]-2-[(N-{[(2,4,5-trifluorophenyl)methoxy]carbonyl}-L-leucyl)amino]propane-1-sulfonic acid	1.79
7TIZ	N63	(1S,2S)-1-hydroxy-3-[(3S)-2-oxopyrrolidin-3-yl]-2-{[N-({[3-(trifluoromethyl)phenyl]methoxy}carbonyl)-L-leucyl]amino}propane-1-sulfonic acid	1.55
7TJ0	S4L	(1S,2S)-2-({N-[(benzyloxy)carbonyl]-3-cyclohexyl-L-alanyl}amino)-1-hydroxy-3-[(3S)-2-oxopyrrolidin-3-yl]propane-1-sulfonic acid	2.17
7TQ2	ISG	N-{(2S)-1-oxo-3-[(3S)-2-oxopyrrolidin-3-yl]propan-2-yl}-N~2~-({[(1R,2R)-2-phenylcyclopropyl]methoxy}carbonyl)-L-leucinamide	2.3
7TQ3	IS5	N~2~-({[(1R,2R)-2-(3-fluorophenyl)cyclopropyl]methoxy}carbonyl)-N-{(2S)-1-oxo-3-[(3S)-2-oxopyrrolidin-3-yl]propan-2-yl}-L-leucinamide	2
7TQ4	IRZ	N~2~-({[(1R,2R)-2-(3-chlorophenyl)cyclopropyl]methoxy}carbonyl)-N-{(2S)-1-oxo-3-[(3S)-2-oxopyrrolidin-3-yl]propan-2-yl}-L-leucinamide	2.45
7U92	M0C	(1R,2S,5S)-N-{(2S,3R)-4-(azetidin-1-yl)-3-hydroxy-4-oxo-1-[(3S)-2-oxopyrrolidin-3-yl]butan-2-yl}-6,6-dimethyl-3-[3-methyl-N-(trifluoroacetyl)-L-valyl]-3-azabicyclo[3.1.0]hexane-2-carboxamide	1.8
7UJG	K36	(1S,2S)-2-({N-[(benzyloxy)carbonyl]-L-leucyl}amino)-1-hydroxy-3-[(3S)-2-oxopyrrolidin-3-yl]propane-1-sulfonic acid	1.8
7UJU	4WI	(1R,2S,5S)-N-{(1E,2S)-1-imino-3-[(3S)-2-oxopyrrolidin-3-yl]propan-2-yl}-6,6-dimethyl-3-[3-methyl-N-(trifluoroacetyl)-L-valyl]-3-azabicyclo[3.1.0]hexane-2-carboxamide	1.85
7UKK	K36	(1S,2S)-2-({N-[(benzyloxy)carbonyl]-L-leucyl}amino)-1-hydroxy-3-[(3S)-2-oxopyrrolidin-3-yl]propane-1-sulfonic acid	2
7UUB	7VB	N-[(benzyloxy)carbonyl]-L-valyl-N-{(2S)-1-hydroxy-3-[(3S)-2-oxopyrrolidin-3-yl]propan-2-yl}-4-methylidene-L-norvalinamide	1.63
7UUC	81L	N-[(benzyloxy)carbonyl]-3-methyl-L-valyl-3-cyclopropyl-N-{(2S)-1-hydroxy-3-[(3S)-2-oxopyrrolidin-3-yl]propan-2-yl}-L-alaninamide	1.6
7UUD	I71	(1R,2S,5S)-3-[N-(tert-butylcarbamoyl)-3-methyl-L-valyl]-N-{(2S,3R)-4-(ethylamino)-3-hydroxy-4-oxo-1-[(3S)-2-oxopyrrolidin-3-yl]butan-2-yl}-6,6-dimethyl-3-azabicyclo[3.1.0]hexane-2-carboxamide	1.85

7UUE	I65	benzyl [(2S,3R)-1-({(2S)-1-[2-(3-amino-3-oxopropyl)-2-propanoylhydrazinyl]-3-cyclohexyl-1-oxopropan-2-yl}amino)-3-tert-butoxy-1-oxobutan-2-yl]carbamate (non-preferred name)	1.85
7VLP	4WI	(1R,2S,5S)-N-{(1E,2S)-1-imino-3-[(3S)-2-oxopyrrolidin-3-yl]propan-2-yl}-6,6-dimethyl-3-[3-methyl-N-(trifluoroacetyl)-L-valyl]-3-azabicyclo[3.1.0]hexane-2-carboxamide	1.502519324
7VLQ	4WI	(1R,2S,5S)-N-{(1E,2S)-1-imino-3-[(3S)-2-oxopyrrolidin-3-yl]propan-2-yl}-6,6-dimethyl-3-[3-methyl-N-(trifluoroacetyl)-L-valyl]-3-azabicyclo[3.1.0]hexane-2-carboxamide	1.939106015
7VVP	80I	[(3~{S})-3-[[(2~{S})-2-[(4-methoxy-1~{H}-indol-2-yl)carbonylamino]-4-methyl-pentanoyl]amino]-2-oxidanylidene-4-[(3~{R})-2-oxidanylidene-3,4-dihydropyrrol-3-yl]butyl] dihydrogen phosphate	1.97
7VVT	80X	N-(3-chlorophenyl)-2-[(2R)-1-ethanoyl-3-oxidanylidene-piperazin-2-yl]ethanamide	2.51
7WO1	3XI	N-[(2S)-3-methyl-1-[[(2S)-4-methyl-1-oxidanylidene-1-[[(2S)-1-oxidanylidene-3-[(3S)-2-oxidanylidenepiperidin-3-yl]propan-2-yl]amino]pentan-2-yl]amino]-1-oxidanylidene-butan-2-yl]cyclohexanecarboxamide	2.15
7WOF	5IZ	(2S,3S)-3-methyl-N-[(2S)-1-oxidanylidene-3-[(3S)-2-oxidanylidenepiperidin-3-yl]propan-2-yl]-2-[[(E)-3-phenylprop-2-enoyl]amino]pentanamide	1.72
7WOH	5IW	(2S)-4-methyl-N-[(2S)-1-oxidanylidene-3-[(3S)-2-oxidanylidenepiperidin-3-yl]propan-2-yl]-2-[[(2S)-3-phenyl-2-[[(E)-3-phenylprop-2-enoyl]amino]propanoyl]amino]pentanamide	1.72
7WQK	ALD	N-[(benzyloxy)carbonyl]-L-leucyl-N-[(2S)-1-hydroxy-4-methylpentan-2-yl]-L-leucinamide	2.15
7WYM	G7L	N-methyl-N-[[4-(trifluoromethyl)-1,3-thiazol-2-yl]methyl]prop-2-enamide	2.05
7WYP	G7O	N-(1,3-benzothiazol-2-ylmethyl)-N-cyclopropyl-prop-2-enamide	2.3
7X6J	QNC	quinoline-2-carboxylic acid	1.5
7X6K	9FF	1H-indole-2-carbaldehyde	2.34
7XAR	BOV	4-fluoranyl-~{N}-[(2~{S})-1-[2-(2-fluoranylethanoyl)-2-[[(3~{S})-2-oxidanylidenepyrrolidin-3-yl]methyl]hydrazinyl]-4-methyl-1-oxidanylidene-pentan-2-yl]-1~{H}-indole-2-carboxamide	1.6
7XRS	HUR	N-[(2S)-1-[[(2S)-1-(1,3-benzothiazol-2-yl)-1-oxidanylidene-3-[(3S)-2-oxidanylidenepyrrolidin-3-yl]propan-2-yl]amino]-4-methyl-1-oxidanylidene-pentan-2-yl]-4-methoxy-1H-indole-2-carboxamide	1.93
7Z0P	I8H	(1~{R},2~{S},5~{S})-3-[(2~{S})-2-(~{tert}-butylcarbamoylamino)-3,3-dimethyl-butanoyl]-6,6-dimethyl-~{N}-[(2~{S},3~{R})-4-(methylamino)-3-oxidanyl-4-oxidanylidene-1-[(3~{S})-2-oxidanylidenepyrrolidin-3-yl]butan-2-yl]-3-azabicyclo[3.1.0]hexane-2-carboxamide	2.52
7Z3U	RN2	Calpeptin	1.72
7Z59	IFO	(3S)-4-[[2,4-bis(fluoranyl)phenyl]methoxy]-2-methyl-4-oxidanylidene-3-[[(Z)-3-oxidanylidene-2-(2-phenoxyethanoylamino)prop-1-enyl]amino]butane-2-sulfinic acid	2
7ZQV	XNV	ethyl (4R)-4-({(2S)-2-[3-{[(5-methyl-1,2-oxazol-3-yl)carbonyl]amino}-2-oxopyridin-1(2H)-yl]pent-4-ynoyl}amino)-5-[(3S)-2-oxopyrrolidin-3-yl]pentanoate	2.26
8A4T	O6K	~{tert}-butyl ~{N}-[1-[(2~{S})-3-cyclopropyl-1-oxidanylidene-1-[[(2~{S},3~{R})-3-oxidanyl-4-oxidanylidene-1-[(3~{S})-2-oxidanylidenepyrrolidin-3-yl]-4-[(phenylmethyl)amino]butan-2-yl]amino]propan-2-yl]-2-oxidanylidene-pyridin-3-yl]carbamate	2.5
8AEB	35J	N-(pyridin-3-ylmethyl)thioformamide	1.83

8AIQ	MXU	~{tert}-butyl ~{N}-[1-[(2~{S})-1-[[(2~{S},3~{R})-4-azanyl-3-oxidanyl-4-oxidanylidene-1-[(3~{S})-2-oxidanylidenepyrrolidin-3-yl]butan-2-yl]amino]-3-cyclopropyl-1-oxidanylidene-propan-2-yl]-2-oxidanylidene-pyridin-3-yl]carbamate	2.19
8AIU	M9X	tert-butyl N-[1-[(2S)-3-cyclopropyl-1-[[(2S,3R)-4-(cyclopropylamino)-3-oxidanyl-4-oxidanylidene-1-[(3S)-2-oxidanylidenepyrrolidin-3-yl]butan-2-yl]amino]-1-oxidanylidene-propan-2-yl]-2-oxidanylidene-pyridin-3-yl]carbamate	1.997
8AJ0	MJ0	(2R,3S)-3-[[(2S)-3-cyclopropyl-2-[2-oxidanylidene-3-(3-phenylpropanoylamino)pyridin-1-yl]propanoyl]amino]-N-methyl-2-oxidanyl-4-[(3S)-2-oxidanylidenepyrrolidin-3-yl]butanamide	2.519
8B2T	OW1	Nirmatrelvir (reacted form)	1.893
8B56	OZI	(2~{S})-4-(2-chloranylethanoyl)-1-(3,4-dichlorophenyl)-~{N}-(thiophen-2-ylmethyl)piperazine-2-carboxamide	1.823
8BGA	QQL	4-methoxy-~{N}-[(2~{S})-4-methyl-1-[[(2~{S})-4-nitro-1-[(3~{S})-2-oxidanylidenepyrrolidin-3-yl]butan-2-yl]amino]-1-oxidanylidene-pentan-2-yl]-1~{H}-indole-2-carboxamide	1.982
8BGD	QH0	(phenylmethyl) N-[(2S)-4-methyl-1-[[(2S)-4-nitro-1-[(3R)-2-oxidanylidenepyrrolidin-3-yl]butan-2-yl]amino]-1-oxidanylidene-pentan-2-yl]carbamate	1.621
8D4P	QAO	2-chloro-N-[(1R)-2-{[2-(3-fluorophenyl)ethyl]amino}-2-oxo-1-(pyridin-3-yl)ethyl]-N-[4-(pentafluoro-lambda~6~-sulfanyl)phenyl]acetamide	2.04
8DIB	TKX	5-bromo-3-[(4-chloro-3-nitrophenyl)methoxy]pyridine-2-carbaldehyde	2.17
8DIC	TNI	5-bromo-3-[(3-bromo-4-chlorophenyl)methoxy]pyridine-2-carbaldehyde	2.09
8DID	U0R	5-bromo-3-[(5-bromo-2-chlorophenyl)methoxy]pyridine-2-carbaldehyde	1.95
8DIE	U1J	5-bromo-3-[(4-methyl-3-nitrophenyl)methoxy]pyridine-2-carbaldehyde	1.9
8DIF	U1R	5-bromo-3-[(naphthalen-2-yl)methoxy]pyridine-2-carbaldehyde	1.98
8DL9	T4V	1-{4-[(naphthalen-1-yl)methyl]piperazin-1-yl}ethan-1-one	1.9
8DLB	SRU	1-[(5S)-5-(3,4-dimethoxyphenyl)-3-phenyl-4,5-dihydro-1H-pyrazol-1-yl]ethan-1-one	1.9
8DMD	SVL	1-[(3R)-4-[(3-chlorophenyl)methyl]-3-(2-methylpropyl)piperazin-1-yl]ethan-1-one	2
8DOX	T2L	(1R,2S,5S)-N-{(1S,2S)-1-(4-fluoro-1,3-benzothiazol-2-yl)-1-hydroxy-3-[(3S)-2-oxopyrrolidin-3-yl]propan-2-yl}-6,6-dimethyl-3-[3-methyl-N-(trifluoroacetyl)-L-valyl]-3-azabicyclo[3.1.0]hexane-2-carboxamide	1.46
8DOY	T1X	7-fluoro-N-[(2S)-1-({(1S,2S)-1-(4-fluoro-1,3-benzothiazol-2-yl)-1-hydroxy-3-[(3S)-2-oxopyrrolidin-3-yl]propan-2-yl}amino)-4-methyl-1-oxopentan-2-yl]-4-methoxy-1H-indole-2-carboxamide	1.59
8DPR	T43	2,2,2-trifluoro-N-{(2S)-1-[(1R,2S,5S)-2-({(2S)-1-(4-fluoro-1,3-benzothiazol-2-yl)-1-oxo-3-[(3S)-2-oxopyrrolidin-3-yl]propan-2-yl}carbamothioyl)-6,6-dimethyl-3-azabicyclo[3.1.0]hexan-3-yl]-3,3-dimethyl-1-oxobutan-2-yl}acetamide	2
8DSU	V2M	N-[(2S)-1-({(2S,3S)-3,4-dihydroxy-1-[(3S)-2-oxopyrrolidin-3-yl]butan-2-yl}amino)-4-methyl-1-oxopentan-2-yl]-4-methoxy-1H-indole-2-carboxamide	1.86
8DZ2	4WI	(1R,2S,5S)-N-{(1E,2S)-1-imino-3-[(3S)-2-oxopyrrolidin-3-yl]propan-2-yl}-6,6-dimethyl-3-[3-methyl-N-(trifluoroacetyl)-L-valyl]-3-azabicyclo[3.1.0]hexane-2-carboxamide	2.129
8DZB	U6Y	benzyl {(3S)-1-[(2S)-1-({(2S,3R)-4-(cyclopropylamino)-3-hydroxy-4-oxo-1-[(3S)-2-oxopyrrolidin-3-yl]butan-2-yl}amino)-4-methyl-1-oxopentan-2-yl]-5-oxopyrrolidin-3-yl}carbamate	1.85
8DZC	U76	(3,5-difluorophenyl)methyl {(3S)-1-[(2S)-1-({(2S,3R)-4-(cyclopropylamino)-3-hydroxy-4-oxo-1-[(3S)-2-oxopyrrolidin-3-	2.2

		yl]butan-2-yl}amino)-4-methyl-1-oxopentan-2-yl]-6-oxopiperidin-3-yl}carbamate	
8EZV	X6O	(1R,2S,5S)-N-{(2S,3R)-4-(azetidin-1-yl)-3-hydroxy-4-oxo-1-[(3S)-2-oxopiperidin-3-yl]butan-2-yl}-6,6-dimethyl-3-[3-methyl-N-(trifluoroacetyl)-L-valyl]-3-azabicyclo[3.1.0]hexane-2-carboxamide	1.8
8EZZ	X70	(1R,2S,5S)-N-{(2S,3R)-4-(3,3-difluoroazetidin-1-yl)-3-hydroxy-4-oxo-1-[(3S)-2-oxopiperidin-3-yl]butan-2-yl}-6,6-dimethyl-3-[3-methyl-N-(trifluoroacetyl)-L-valyl]-3-azabicyclo[3.1.0]hexane-2-carboxamide	1.85
8F02	X6T	(1R,2S,5S)-N-{(2S,3R)-4-(3,3-dimethylazetidin-1-yl)-3-hydroxy-4-oxo-1-[(3S)-2-oxopiperidin-3-yl]butan-2-yl}-6,6-dimethyl-3-[3-methyl-N-(trifluoroacetyl)-L-valyl]-3-azabicyclo[3.1.0]hexane-2-carboxamide	2
8F2C	X9Z	(1R,2S,5S)-N-[(2S,3R)-4-(azetidin-1-yl)-3-hydroxy-4-oxo-1-(2-oxopyrrolidin-1-yl)butan-2-yl]-6,6-dimethyl-3-[3-methyl-N-(trifluoroacetyl)-L-valyl]-3-azabicyclo[3.1.0]hexane-2-carboxamide	1.95
8F2D	XA8	(1R,2S,5S)-N-[(2S,3R)-4-(azetidin-1-yl)-3-hydroxy-4-oxo-1-(2-oxopiperidin-1-yl)butan-2-yl]-6,6-dimethyl-3-[3-methyl-N-(trifluoroacetyl)-L-valyl]-3-azabicyclo[3.1.0]hexane-2-carboxamide	1.95
8F45	XF8	(2-methyl-2-phenylsulfanyl-propyl) ~{N}-[(2~{S})-1-[[(2~{S},3~{S})-3-[bis(oxidanyl)-oxidanylidene-$l^{5}-sulfanyl]-4-(cyclopropylamino)-3-oxidanyl-4-oxidanylidene-1-[(3~{S})-2-oxidanylidenepyrrolidin-3-yl]butan-2-yl]amino]-4-methyl-1-oxidanylidene-pentan-2-yl]carbamate	1.65
8F46	XCK	N-{(1Z,2S)-1-imino-3-[(3S)-2-oxopyrrolidin-3-yl]propan-2-yl}-N~2~-{[2-methyl-2-(phenylsulfanyl)propoxy]carbonyl}-L-leucinamide	1.5
8FIV	Y0I	(3Z)-N-([1,1'-biphenyl]-4-yl)-3-imino-N-[(1R)-2-oxo-2-{[(1S)-1-phenylethyl]amino}-1-(pyridin-3-yl)ethyl]propanamide	2.51
8FIW	Y0E	N-([1,1'-biphenyl]-4-yl)-N-[(1R)-2-oxo-2-{[(1S)-1-phenylethyl]amino}-1-(pyridin-3-yl)ethyl]prop-2-enamide	2.54
8FTC	Y8O	(1R,2S,5S)-3-[N-(difluoroacetyl)-3-methyl-L-valyl]-N-{(1Z,2S)-1-imino-3-[(3S)-2-oxopiperidin-3-yl]propan-2-yl}-6,6-dimethyl-3-azabicyclo[3.1.0]hexane-2-carboxamide	2
8FTL	5ZF	N-([1,1'-biphenyl]-4-yl)-2-chloro-N-[(1R)-2-oxo-2-{[(1S)-1-phenylethyl]amino}-1-(pyridin-3-yl)ethyl]acetamide	2.08
8FY6	OW1	Nirmatrelvir (reacted form)	2
8FY7	YFK	4-methoxy-N-[(2S)-4-methyl-1-oxo-1-({(2S)-1-[(3S)-2-oxopyrrolidin-3-yl]but-3-en-2-yl}amino)pentan-2-yl]-1H-indole-2-carboxamide	1.94
8GXG	06Q	N-[(2S)-3-(4-fluorophenyl)-1-oxidanylidene-1-[[(2S,3S)-3-oxidanyl-4-oxidanylidene-1-[(3S)-2-oxidanylidenepiperidin-3-yl]-4-[(phenylmethyl)amino]butan-2-yl]amino]propan-2-yl]-1-benzofuran-2-carboxamide	1.69
8GXH	0AX	N-[(2S)-3-cyclohexyl-1-oxidanylidene-1-[[(2S,3R)-3-oxidanyl-4-oxidanylidene-1-[(3S)-2-oxidanylidenepiperidin-3-yl]-4-[(phenylmethyl)amino]butan-2-yl]amino]propan-2-yl]-1-benzofuran-2-carboxamide	1.59
8GXI	0BO	N-[(2S)-3-cyclohexyl-1-[[(2S,3R)-4-(cyclopropylamino)-3-oxidanyl-4-oxidanylidene-1-[(3S)-2-oxidanylidenepiperidin-3-yl]butan-2-yl]amino]-1-oxidanylidene-propan-2-yl]-1-benzofuran-2-carboxamide	1.69
8GZB	KM6	2-(4-chlorophenyl)-1,3,4-oxadiazole	2.7
8HHT	LV0	~{N}-[(2~{R},3~{S})-3-oxidanyl-4-oxidanylidene-1-phenyl-4-(1,3-thiazol-2-ylmethylamino)butan-2-yl]benzamide	1.95
8HI9	LKR	3,7-bis(oxidanyl)-2-[3,4,5-tris(oxidanyl)phenyl]chromen-4-one	2.28
8HTV	UZF	1-(5,6-dihydrobenzo[b][1]benzazepin-11-yl)-2-sulfanyl-ethanone	2.04
8I30	OF9	(2~{R})-1-[4,4-bis(fluoranyl)cyclohexyl]carbonyl-4,4-bis(fluoranyl)-~{N}-[(2~{R},3~{S})-3-oxidanyl-4-oxidanylidene-1-phenyl-4-(pyridin-2-ylmethylamino)butan-2-yl]pyrrolidine-2-carboxamide	2
8IFP	OZ6	(1R,2S,5S)-3-[(2S)-2-(tert-butylcarbamoylamino)-3,3-dimethyl-butanoyl]-6,6-dimethyl-N-[(2S)-1-oxidanylidene-3-[(3S)-2-	1.78

		oxidanylidenepyrrolidin-3-yl]propan-2-yl]-3-azabicyclo[3.1.0]hexane-2-carboxamide	
8IFQ	I1Z	(1R,2S,5S)-3-[N-(tert-butylcarbamoyl)-3-methyl-L-valyl]-N-{(1Z,2S)-1-imino-3-[(3S)-2-oxopyrrolidin-3-yl]propan-2-yl}-6,6-dimethyl-3-azabicyclo[3.1.0]hexane-2-carboxamide	1.96
8IFR	P0O	(1R,2S,5S)-3-[(2S)-2-(tert-butylcarbamoylamino)-3,3-dimethyl-butanoyl]-6,6-dimethyl-N-[(2S)-5-oxidanylidene-1-[(3S)-2-oxidanylidenepyrrolidin-3-yl]hex-3-en-2-yl]-3-azabicyclo[3.1.0]hexane-2-carboxamide	1.66
8IFS	OZL	(8~{S})-7-[(2~{S})-2-(~{tert}-butylcarbamoylamino)-3,3-dimethyl-butanoyl]-~{N}-[(1~{S})-1-cyano-2-[(3~{S})-2-oxidanylidenepyrrolidin-3-yl]ethyl]-1,4-dithia-7-azaspiro[4.4]nonane-8-carboxamide	2.46
8IFT	OZB	(8S)-N-[(1S)-1-cyano-2-[(3S)-2-oxidanylidenepyrrolidin-3-yl]ethyl]-7-[(2S)-2-[(1-fluoranylcyclopropyl)carbonylamino]-3,3-dimethyl-butanoyl]-1,4-dithia-7-azaspiro[4.4]nonane-8-carboxamide	1.8
8IGN	7ON	(3~{S},3~{a}~{S},6~{a}~{R})-2-[(2~{S})-2-cyclohexyl-2-[2,2,2-tris(fluoranyl)ethanoylamino]ethanoyl]-~{N}-[(2~{S})-4-(cyclopentylamino)-3,4-bis(oxidanylidene)-1-[(3~{S})-2-oxidanylidenepyrrolidin-3-yl]butan-2-yl]-3,3~{a},4,5,6,6~{a}-hexahydro-1~{H}-cyclopenta[c]pyrrole-3-carboxamide	2.02
8IGX	PQL	(8~{S})-~{N}-[(1~{S})-1-cyano-2-[(3~{S})-2-oxidanylidenepyrrolidin-3-yl]ethyl]-7-[(2~{S})-3,3-dimethyl-2-[2,2,2-tris(fluoranyl)ethanoylamino]butanoyl]-1,4-dithia-7-azaspiro[4.4]nonane-8-carboxamide	1.9
8IGY	4WI	(1R,2S,5S)-N-{(1E,2S)-1-imino-3-[(3S)-2-oxopyrrolidin-3-yl]propan-2-yl}-6,6-dimethyl-3-[3-methyl-N-(trifluoroacetyl)-L-valyl]-3-azabicyclo[3.1.0]hexane-2-carboxamide	1.96
8JOP	UWO	methyl (6~{R})-5-ethanoyl-7-oxidanylidene-6-[4-(trifluoromethyl)phenyl]-8,9,10,11-tetrahydro-6~{H}-benzo[b][1,4]benzodiazepine-2-carboxylate	2.7
8OKB	VQR	methyl (4~{S})-4-[[(2~{S})-4-methyl-2-(phenylmethoxycarbonylamino)pentanoyl]amino]-5-[(3~{S})-2-oxidanylidenepyrrolidin-3-yl]pentanoate	2.31
8OKC	VQN	(phenylmethyl) ~{N}-[(2~{R})-1-[[(~{Z},2~{S})-5-[4-[[1-[2-[(3~{R})-2,6-bis(oxidanylidene)piperidin-3-yl]-6-fluoranyl-1,3-bis(oxidanylidene)isoindol-5-yl]piperidin-4-yl]methyl]piperazin-1-yl]-5-oxidanylidene-1-[(3~{R})-2-oxidanylidenepyrrolidin-3-yl]pent-3-en-2-yl]amino]-4-methyl-1-oxidanylidene-pentan-2-yl]carbamate	2
8OKK	83F	tert-butyl-N-[(2S)-3-methyl-1-[(2S,4S)-4-methyl-2-[[(2S)-1-oxidanyl-3-[(3S)-2-oxidanylidenepyrrolidin-3-yl]propan-2-yl]carbamoyl]pyrrolidin-1-yl]-1-oxidanylidene-butan-2-yl]carbamate	1.63
8OKL	83N	tert-butyl-N-[(2S)-1-[(2S,4S)-4-methoxy-2-[[(2S)-1-oxidanyl-3-[(3S)-2-oxidanylidenepyrrolidin-3-yl]propan-2-yl]carbamoyl]pyrrolidin-1-yl]-3-methyl-1-oxidanylidene-butan-2-yl]carbamate	1.5
8OKM	84C	tert-butyl-N-[(2S)-1-[(3S,3aS,6aR)-3-[[(2S)-1-oxidanyl-3-[(3S)-2-oxidanylidenepyrrolidin-3-yl]propan-2-yl]carbamoyl]-3,3a,4,5,6,6a-hexahydro-1H-cyclopenta[c]pyrrol-2-yl]-3-methyl-1-oxidanylidene-butan-2-yl]carbamate	1.66
8OKN	83W	tert-butyl-N-[(2S,3R)-3-[(2-methylpropan-2-yl)oxy]-1-oxidanylidene-1-[(2S)-2-[[(2S)-1-oxidanyl-3-[(3S)-2-oxidanylidenepyrrolidin-3-yl]propan-2-yl]carbamoyl]pyrrolidin-1-yl]butan-2-yl]carbamate	1.35
8SK4	I3R	2-chloro-1-[(5R)-3-phenyl-5-(quinoxalin-5-yl)-4,5-dihydro-1H-pyrazol-1-yl]ethan-1-one	2
8SKH	A1W	2-chloro-1-[(4R,5R)-3,4,5-triphenyl-4,5-dihydro-1H-pyrazol-1-yl]ethan-1-one	1.882

8STY	WGE	benzyl (3S)-3-({(2S)-1-hydroxy-3-[(3S)-2-oxopyrrolidin-3-yl]propan-2-yl}carbamoyl)-2-azaspiro[4.4]nonane-2-carboxylate	1.9
8STZ	WGI	benzyl (3S)-3-({(2S)-1-hydroxy-3-[(3S)-2-oxopyrrolidin-3-yl]propan-2-yl}carbamoyl)-2-azaspiro[4.5]decane-2-carboxylate	1.85
8TBE	ZQB	Pomotrelvir bound form	2.15
8TPB	JVX	N-[(1R)-2-(tert-butylamino)-2-oxo-1-(pyridin-3-yl)ethyl]-N-(4-tert-butylphenyl)-2-chloroacetamide	1.88
8TPC	JJC	N-[(1R)-2-(benzylamino)-2-oxo-1-(pyridin-3-yl)ethyl]-N-[4-(2-chloroacetamido)phenyl]furan-2-carboxamide	1.73
8TPD	JJO	N-[(1R)-2-(benzylamino)-2-oxo-1-(pyridin-3-yl)ethyl]-N-[3-(2-chloroacetamido)phenyl]furan-2-carboxamide	1.68
8TPE	JK0	N-[(1R)-2-(benzylamino)-2-oxo-1-(pyridin-3-yl)ethyl]-N-(4-tert-butylphenyl)-3-hydroxypropanamide	1.61
8TPF	JWI	N-(4-tert-butylphenyl)-N-[(1R)-2-(cyclohexylamino)-2-oxo-1-(pyridin-3-yl)ethyl]-3-hydroxypropanamide	1.95
8TPH	JKL	(3R)-N-(4-tert-butylphenyl)-N-[(1R)-2-(cyclohexylamino)-2-oxo-1-(pyridin-3-yl)ethyl]-3-hydroxybutanamide	1.52
8TPI	JWO	N-(4-tert-butylphenyl)-N-[(1R)-2-(cyclohexylamino)-2-oxo-1-(pyridin-3-yl)ethyl]-2-hydroxy-2-methylpropanamide	1.98
8UAB	W28	N-[(2S)-1-({(2S)-1-hydroxy-3-[(3S)-2-oxopyrrolidin-3-yl]propan-2-yl}amino)-4-methyl-1-oxopentan-2-yl]-1H-indole-2-carboxamide	1.781
8UH5	WOK	(1R,2S,5S)-N-{(1S,2S)-1-(5-fluoro-1,3-benzothiazol-2-yl)-1-hydroxy-3-[(3S)-2-oxopyrrolidin-3-yl]propan-2-yl}-6,6-dimethyl-3-[3-methyl-N-(trifluoroacetyl)-L-valyl]-3-azabicyclo[3.1.0]hexane-2-carboxamide	1.74
8UIA	WTV	N-[(benzyloxy)carbonyl]-4-fluoro-L-phenylalanyl-N-{(2R)-1-[(2R)-oxolan-2-yl]-3-[(3R)-2-oxooxolan-3-yl]propan-2-yl}-L-leucinamide	1.75
8UIF	A1ADS	N-[(benzyloxy)carbonyl]-4-fluoro-L-phenylalanyl-N-{(2R)-1-[(2S)-oxolan-2-yl]-3-[(3S)-2-oxooxolan-3-yl]propan-2-yl}-L-leucinamide	2.02

**615 Non-covalent Inhibitors**			
PDB ID	Ligand ID	Ligand Name	Resolution (Å)
5R7Y	JFM	N-(2-phenylethyl)methanesulfonamide	1.65
5R7Z	HWH	~{N}-[2-(5-fluoranyl-1~{H}-indol-3-yl)ethyl]ethanamide	1.59
5R80	RZG	methyl 4-sulfamoylbenzoate	1.93

5R81	RZJ	1-methyl-3,4-dihydro-2~{H}-quinoline-7-sulfonamide	1.95
5R82	RZS	6-(ethylamino)pyridine-3-carbonitrile	1.31
5R83	K0G	N-phenyl-N'-pyridin-3-ylurea	1.58
5R84	GWS	2-cyclohexyl-~{N}-pyridin-3-yl-ethanamide	1.83
5RE4	SZY	N-(4-methylpyridin-3-yl)acetamide	1.88
5RE5	T0J	N~1~-phenylpiperidine-1,4-dicarboxamide	2.07
5RE6	O0S	N-{4-[(pyrimidin-2-yl)oxy]phenyl}acetamide	1.87
5RE7	T0S	N-[(4-sulfamoylphenyl)methyl]acetamide	1.79
5RE8	T0V	1-(3-fluorophenyl)-N-[(furan-2-yl)methyl]methanamine	1.81
5RE9	LPZ	2-(4-methylphenoxy)-1-(4-methylpiperazin-4-ium-1-yl)ethanone	1.72
5REA	JGP	(azepan-1-yl)(2H-1,3-benzodioxol-5-yl)methanone	1.63
5REB	T0Y	1-[(thiophen-3-yl)methyl]piperidin-4-ol	1.68
5REC	T1J	2-{[(1H-benzimidazol-2-yl)amino]methyl}phenol	1.73
5RED	JJG	4-[2-(phenylsulfanyl)ethyl]morpholine	1.47
5REE	T1M	(2R,3R)-1-benzyl-2-methylpiperidin-3-ol	1.77
5REF	6SU	methyl 3-(methylsulfonylamino)benzoate	1.61
5REG	LWA	(2~{S})-~{N}-(4-aminocarbonylphenyl)oxolane-2-carboxamide	1.67
5REH	AWP	1-cyclohexyl-3-(2-pyridin-4-ylethyl)urea	1.8
5REI	T1S	4-[(3-chlorophenyl)methyl]morpholine	1.82
5REJ	T1V	1-{4-[(thiophen-2-yl)sulfonyl]piperazin-1-yl}ethan-1-one	1.72
5REK	T1Y	1-{4-[(3-fluorophenyl)sulfonyl]piperazin-1-yl}ethan-1-one	1.74
5REL	T2G	1-{4-[(3-methylphenyl)methyl]piperazin-1-yl}ethan-1-one	1.62
5REM	T2J	1 1-(4-(2-nitrophenyl)piperazin-1-yl)ethan-1-one	1.96
5REN	T2V	1-[(3R)-3-(1,3-benzothiazol-2-yl)piperidin-1-yl]ethan-1-one	2.15
5REO	T2Y	N-[(2H-1,3-benzodioxol-5-yl)methyl]acetamide	1.88
5REP	T3G	1-{4-[(2,6-difluorophenyl)sulfonyl]piperazin-1-yl}ethan-1-one	1.81
5RER	T3J	1-[(2R)-2-(4-fluorophenyl)morpholin-4-yl]ethan-1-one	1.88
5RES	T3V	1-{4-[(2-fluorophenyl)sulfonyl]piperazin-1-yl}ethan-1-one	1.65
5RET	T47	1-{4-[(3-chlorophenyl)methyl]piperazin-1-yl}ethan-1-one	1.68
5REU	T4D	2-[(4-acetylpiperazin-1-yl)sulfonyl]benzonitrile	1.69
5REV	T4J	N-[3-(thiomorpholine-4-carbonyl)phenyl]acetamide	1.6
5REW	T4M	N-[(1R)-1-(naphthalen-1-yl)ethyl]acetamide	1.55
5REX	T4V	1-{4-[(naphthalen-1-yl)methyl]piperazin-1-yl}ethan-1-one	2.07
5REY	T4Y	1-{4-[(2-methylphenyl)methyl]-1,4-diazepan-1-yl}ethan-1-one	1.96
5REZ	T54	(1R,2S)-2-(thiophen-3-yl)cyclopentane-1-carboxamide	1.79
5RF0	T5D	[1-(pyridin-2-yl)cyclopentyl]methanol	1.65
5RF1	T5G	4-bromobenzene-1-sulfonamide	1.73
5RF2	HVB	1-azanylpropylideneazanium	1.53
5RF3	T5V	pyrimidin-5-amine	1.5
5RF4	T5Y	pyridin-2-ol	1.61
5RF5	HV2	1,1-bis(oxidanylidene)thietan-3-ol	1.74
5RF6	NTG	5-(1,4-oxazepan-4-yl)pyridine-2-carbonitrile	1.45
5RF7	T67	1-(4-methylpiperazin-1-yl)-2-(1H-pyrrolo[2,3-b]pyridin-3-yl)ethan-1-one	1.54
5RF8	SFY	4-amino-N-(pyridin-2-yl)benzenesulfonamide	1.44
5RF9	S7D	1-[(2~{S})-2-methylmorpholin-4-yl]-2-pyrazol-1-yl-ethanone	1.43
5RFA	JGY	1-methyl-N-{[(2S)-oxolan-2-yl]methyl}-1H-pyrazole-3-carboxamide	1.52
5RFB	K3S	N-[(1-methyl-1H-1,2,3-triazol-4-yl)methyl]ethanamine	1.48
5RFC	K1Y	methyl (2-methyl-4-phenyl-1,3-thiazol-5-yl)carbamate	1.4
5RFD	T6J	2-[(methylsulfonyl)methyl]-1H-benzimidazole	1.41
5RFE	JGG	N-[(4-cyanophenyl)methyl]morpholine-4-carboxamide	1.46
5RG1	T9J	Nalpha-acetyl-N-(3-bromoprop-2-yn-1-yl)-L-tyrosinamide	1.65
5RGG	NZD	4-methyl-N-phenylpiperazine-1-carboxamide	2.26

5RGH	U0M	5-fluoro-1-[(5-methyl-1,3,4-thiadiazol-2-yl)methyl]-1,2,3,6-tetrahydropyridine	1.7
5RGI	U0P	N'-cyclopropyl-N-methyl-N-[(5-methyl-1,2-oxazol-3-yl)methyl]urea	1.57
5RGJ	U0S	(5S)-7-(pyrazin-2-yl)-2-oxa-7-azaspiro[4.4]nonane	1.34
5RGK	U0V	2-fluoro-N-[2-(pyridin-4-yl)ethyl]benzamide	1.43
5RGQ	U1V	1-(4-fluoro-2-methylphenyl)methanesulfonamide	2.15
5RGS	S7V	[(2~{R})-4-(phenylmethyl)morpholin-2-yl]methanol	1.72
5RGU	UGD	N-(3-{[(2R)-4-oxoazetidin-2-yl]oxy}phenyl)-2-(pyrimidin-5-yl)acetamide	2.108
5RGV	UGG	2-(isoquinolin-4-yl)-N-phenylacetamide	1.82
5RGW	UGM	2-(5-cyanopyridin-3-yl)-N-(pyridin-3-yl)acetamide	1.43
5RGX	UGP	2-(3-cyanophenyl)-N-(4-methylpyridin-3-yl)acetamide	1.69
5RGY	UGS	N-(4-methoxypyridin-2-yl)-2-(naphthalen-2-yl)acetamide	1.976
5RGZ	UH1	2-(3-cyanophenyl)-N-(pyridin-3-yl)acetamide	1.52
5RH0	UH4	N-(5-methylthiophen-2-yl)-N'-pyridin-3-ylurea	1.916
5RH1	UGV	2-(5-chlorothiophen-2-yl)-N-(pyridin-3-yl)acetamide	1.96
5RH2	UH7	2-(3-chlorophenyl)-N-(4-methylpyridin-3-yl)acetamide	1.827
5RH3	UHA	(2R)-2-(3-chlorophenyl)-N-(4-methylpyridin-3-yl)propanamide	1.69
5RH4	UHG	(2R)-2-(6-chloro-9H-carbazol-2-yl)propanoic acid	1.34
5RH8	UHM	2-(cyanomethoxy)-N-[(1,2-thiazol-4-yl)methyl]benzamide	1.81
5SML	O3R	6-{[(3,4-dichlorophenyl)methyl](methyl)amino}pyridine-3-sulfonamide	1.53
5SMM	O46	N-[4-(3-fluorophenyl)oxan-4-yl]-2-(3-hydroxyphenyl)acetamide	1.58
5SMN	O4F	N-(1-cyanocyclopropyl)-1-(3-methylpyridin-4-yl)piperidine-4-carboxamide	1.36
6M2N	3WL	5,6,7-trihydroxy-2-phenyl-4H-chromen-4-one	2.198
6W63	X77	N-(4-tert-butylphenyl)-N-[(1R)-2-(cyclohexylamino)-2-oxo-1-(pyridin-3-yl)ethyl]-1H-imidazole-4-carboxamide	2.1
6YVF	A82	2-[[(1R)-1-(7-methyl-2-morpholin-4-yl-4-oxidanylidene-pyrido[1,2-a]pyrimidin-9-yl)ethyl]amino]benzoic acid	1.6
7ABU	R6Q	1'-[2-[4-(trifluoromethyl)phenyl]ethyl]spiro[1~{H}-3,1-benzoxazine-4,4'-piperidine]-2-one	1.6
7AF0	R9W	2,3,5,6,7,8-hexahydro-1~{H}-cyclopenta[b]quinolin-9-amine	1.7
7AGA	LZE	4-{[(2,6-dichlorophenyl)carbonyl]amino}-N-piperidin-4-yl-1H-pyrazole-3-carboxamide	1.68
7AMJ	RMZ	(3~{S})-3-[2-[4-(3,4-dimethylphenyl)piperazin-1-yl]ethyl]-2,3-dihydroisoindol-1-one	1.59
7ANS	RNW	2-[(diphenylmethyl)-oxidanyl-$l^{3}-sulfanyl]-~{N}-oxidanyl-ethanamide	1.7
7AP6	RQN	4-(4-ethyl-5-fluoranyl-2-oxidanyl-phenoxy)-3-fluoranyl-benzamide	1.78
7APH	RT2	Tofogliflozin	1.65
7AQE	RV5	N-1,2,3-Benzothiadiazol-6-yl-N'-[2-oxo-2-(1-piperidinyl)ethyl]urea also called unc-2327	1.39
7AQI	QEL	4-[(1R,2S)-2-(4-benzylpiperidin-1-yl)-1-hydroxypropyl]phenol	1.7
7AVD	S1W	3-[[5-[3-(dimethylamino)phenoxy]pyrimidin-2-yl]amino]phenol	1.8
7AWR	S7W	TEGAFUR	1.34
7AWW	CLU	2,6-DICHLORO-N-IMIDAZOLIDIN-2-YLIDENEANILINE	1.65
7AXM	93J	(2E)-N-{4-[(3-chloro-4-fluorophenyl)amino]-3-cyano-7-ethoxyquinolin-6-yl}-4-(dimethylamino)but-2-enamide	1.4
7AXO	QCP	AR-42	1.65
7B2J	SQ2	2-(1H-1,2,3-benzotriazol-1-yl)-1-(4-methylpiperidin-1-yl)ethan-1-one	1.55

7B2U	SQ5	(5S)-5-(cyclohexylmethyl)-3-(5-fluoropyridin-3-yl)imidazolidine-2,4-dione	1.55
7B5Z	SYH	2-(1H-benzo[d][1,2,3]triazol-1-yl)-1-(4-methylenepiperidin-1-yl)ethan-1-one	1.65
7B77	T0W	2-(benzotriazol-1-yl)-~{N}-ethyl-~{N}-(furan-3-ylmethyl)ethanamide	1.6
7BIJ	TU8	(3~{S})-3'-(5-fluoranylpyridin-3-yl)spiro[1,2-dihydroindene-3,5'-imidazolidine]-2',4'-dione	1.47
7DDC	H3F	Tafenoquine	2.175
7EN8	J7R	~{N}-[(1~{S},2~{R})-2-[[4-bromanyl-2-(methylcarbamoyl)-6-nitro-phenyl]amino]cyclohexyl]isoquinoline-4-carboxamide	1.83
7EN9	J7O	5-bromanyl-~{N}-methyl-3-nitro-2-[(4~{R},5~{S})-2-(7-oxidanylisoquinolin-4-yl)carbonyl-4-phenyl-2,7-diazaspiro[4.4]nonan-7-yl]benzamide	1.9
7GAV	KFU	(3S)-5-chloro-N-(isoquinolin-4-yl)-2,3-dihydro-1-benzofuran-3-carboxamide	1.77
7GAW	KG9	(4S)-6-chloro-2-[(1-cyanocyclopropyl)methanesulfonyl]-N-(isoquinolin-4-yl)-1,2,3,4-tetrahydroisoquinoline-4-carboxamide	1.812
7GAX	KJI	N-(4-methylpyridin-3-yl)-N~2~-(quinolin-4-yl)glycinamide	1.71
7GAY	KJO	N-phenyl-2-(pyridin-3-yl)prop-2-enamide	1.3
7GAZ	KL6	1-{2-[(methanesulfonyl)amino]ethyl}-1,2,3,4-tetrahydroquinoline-7-sulfonamide	1.753
7GB0	KLR	(2S)-N-tert-butyl-2-[4-(2-cyanoethyl)anilino]-2-(pyridin-3-yl)acetamide	1.423
7GB4	KO9	N-(5-cyanopyridin-3-yl)-2-(pyridin-3-yl)acetamide	1.865
7GB5	KOI	2-(3-chlorophenyl)-N-(pyridin-3-yl)acetamide	1.261
7GB6	KP0	N-(3-chlorophenyl)-N-(2-cyclohexylethyl)-N'-(pyridin-3-yl)urea	1.84
7GB7	KQ3	N-(3-chlorophenyl)-N-[2-(morpholin-4-yl)ethyl]-N'-(pyridin-3-yl)urea	1.55
7GB8	KQL	N-(4-methylpyridin-3-yl)-2-[3-(trifluoromethyl)phenyl]acetamide	1.957
7GB9	KQX	2-(4-methylphenyl)-N-(4-methylpyridin-3-yl)acetamide	1.4
7GBC	KSX	2-(5-cyanopyridin-3-yl)-N-(4-methylpyridin-3-yl)acetamide	1.655
7GBD	KT9	N-(3-methyl-5-{[(2S)-4-oxoazetidin-2-yl]oxy}phenyl)-2-(pyrimidin-5-yl)acetamide	1.78
7GBE	KU6	(4R)-6-chloro-N-(4-cyclopropyl-4H-1,2,4-triazol-3-yl)-3,4-dihydro-2H-1-benzopyran-4-carboxamide	1.224
7GBF	KUU	(2R)-2-(3-chlorophenyl)-3-methyl-N-(4-methylpyridin-3-yl)butanamide	1.769
7GBG	KVF	(2S)-2-(3-chlorophenyl)-N-(5-methylpyridazin-4-yl)butanamide	1.461
7GBH	KVO	2-(3-chlorophenyl)-N-(2,4-dimethylpyridin-3-yl)acetamide	1.581
7GBI	KVX	(3S)-5-chloro-N-[4-(hydroxymethyl)pyridin-3-yl]-2,3-dihydro-1-benzofuran-3-carboxamide	1.291
7GBJ	KW9	7-fluoro-N-[2-(2-methoxyphenoxy)ethyl]-2-oxo-1,2-dihydroquinoline-4-carboxamide	1.549
7GBK	KXF	2-(3-hydroxyphenyl)-N-(4-methylpyridin-3-yl)acetamide	1.5
7GBL	KWR	N-(3-{[(2S)-4-oxoazetidin-2-yl]oxy}phenyl)-2-(pyridin-3-yl)acetamide	2.16
7GBM	KX9	(2R)-2-(3-chlorophenyl)-N-[(4M)-4-(1H-pyrazol-1-yl)pyridin-3-yl]propanamide	1.8
7GBN	KXR	N-(3-fluoro-5-{[(2S)-4-oxoazetidin-2-yl]oxy}phenyl)-2-(pyrimidin-5-yl)acetamide	1.944
7GBO	KY0	1-(3-chlorophenyl)-N-(4-methylpyridin-3-yl)cyclopropane-1-carboxamide	1.619
7GBP	KYC	(2S)-2-(3-chlorophenyl)-2-(dimethylamino)-N-(4-methylpyridin-3-yl)acetamide	1.7

7GBQ	KYU	2-(3-chlorophenyl)-2,2-difluoro-N-(4-methylpyridin-3-yl)acetamide	1.72
7GBR	KZC	N-(2-anilinoethyl)-2-oxo-1,2-dihydroquinoline-4-carboxamide	1.49
7GBS	KZX	2-(3-fluorophenyl)-N-(4-methylpyridin-3-yl)acetamide	1.54
7GBT	L1F	N-[2-(2-methoxyphenoxy)ethyl]-N-methyl-2-oxo-1,2-dihydroquinoline-4-carboxamide	1.25
7GBU	L2I	(2S)-4-(methylamino)-2-phenyl-N-(pyridin-3-yl)butanamide	1.559
7GBV	L3I	2-(3-chlorophenyl)-2-methyl-N-(4-methylpyridin-3-yl)propanamide	1.67
7GBW	L6R	(2R)-2-(3-chlorophenyl)-N-(4-methylpyridin-3-yl)pentanamide	1.559
7GBX	L6D	N-(1H-benzimidazol-1-yl)-2-(3-chlorophenyl)acetamide	1.91
7GBY	L5I	(2R)-3-cyclopropyl-2-methyl-N-(4-methylpyridin-3-yl)propanamide	1.608
7GBZ	L4U	(3S)-3,4-dimethyl-N-(4-methylpyridin-3-yl)pentanamide	1.529
7GC0	L4N	(5R)-N-(4-methylpyridin-3-yl)spiro[2.4]heptane-5-carboxamide	1.579
7GC1	L7F	2-[(1S,5R)-bicyclo[3.1.0]hexan-1-yl]-N-(4-methylpyridin-3-yl)acetamide	1.59
7GC2	L7Q	3-methyl-N-(4-methylpyridin-3-yl)-3-phenylbutanamide	1.731
7GC3	L7V	1-[(2S)-2-(5-cyclopropyl-1,2,4-oxadiazol-3-yl)pyrrolidin-1-yl]-2-(pyridin-3-yl)ethan-1-one	1.56
7GC4	L83	N-(2-amino-4-methylpyridin-3-yl)-2-(3-chlorophenyl)acetamide	1.736
7GC7	L93	6-fluoro-N-[(2R)-2-(2-methoxyphenoxy)propyl]-2-oxo-1,2-dihydroquinoline-4-carboxamide	1.65
7GC8	L9F	(3P,5R)-3-(3-chlorophenyl)-5-(pyridin-3-yl)imidazolidine-2,4-dione	1.801
7GC9	L9O	(1S)-N-(4-methylpyridin-3-yl)spiro[3.3]heptane-1-carboxamide	1.771
7GCA	LB0	(1r,3r)-3-cyclopropyl-N-(4-methylpyridin-3-yl)cyclobutane-1-carboxamide	1.771
7GCB	LBC	2-(3-iodophenyl)-N-(4-methylpyridin-3-yl)acetamide	1.608
7GCC	LBO	2-(3-cyclopropylphenyl)-N-(4-methylpyridin-3-yl)acetamide	1.61
7GCD	LCU	2-[(1R,3s,5S)-bicyclo[3.1.0]hexan-3-yl]-N-(4-methylpyridin-3-yl)acetamide	1.811
7GCE	LDX	3-(2-fluorophenyl)-N-(4-methylpyridin-3-yl)propanamide	1.39
7GCF	LKX	2-(3-chlorophenyl)-N-(5-oxo-1,5-dihydro-4H-1,2,4-triazol-4-yl)acetamide	1.419
7GCG	LKI	3-chloro-N-(4-methylpyridin-3-yl)benzene-1-sulfonamide	1.887
7GCI	LJO	(3R)-3-cyano-N-(4-methylpyridin-3-yl)oxolane-3-carboxamide	1.541
7GCJ	LJ0	(1R,6S,7r)-N-(4-methylpyridin-3-yl)bicyclo[4.1.0]heptane-7-carboxamide	1.34
7GCK	LF3	2-(6-chloro-3-oxo-2,3-dihydro-4H-1,4-benzoxazin-4-yl)-N-(4-methylpyridin-3-yl)acetamide	1.4
7GCL	LR0	(7R)-N-(4-acetamidopyridin-3-yl)-4-fluorobicyclo[4.2.0]octa-1,3,5-triene-7-carboxamide	1.829
7GCN	LQ0	2-(6-chloro-1H-indol-1-yl)-N-(4-methylpyridin-3-yl)acetamide	1.62
7GCO	LO0	N-[2-(2-methoxyphenoxy)ethyl]-2-oxo-2,3-dihydropyridine-4-carboxamide	1.59
7GCQ	LRC	2-(5-chloropyridin-3-yl)-N-(4-methylpyridin-3-yl)acetamide	1.693
7GCS	LS0	2-(6-fluoro-1H-indol-1-yl)-N-(4-methylpyridin-3-yl)acetamide	1.618
7GCT	LSF	N-(4-methylpyridin-3-yl)-2-(3-{[(2R)-4-oxoazetidin-2-yl]oxy}phenyl)acetamide	1.857
7GCU	LT9	1-(3-chlorophenyl)-N-(4-methylpyridin-3-yl)-3-oxocyclobutane-1-carboxamide	1.541
7GCV	LUC	2-(3-chloro-5-{[(2R)-4-oxoazetidin-2-yl]oxy}phenyl)-N-(4-methylpyridin-3-yl)acetamide	1.964
7GCW	LV9	N-(4-benzyloxan-4-yl)-N'-(pyridin-3-yl)urea	1.58

7GCX	LVM	N-(4-methylpyridin-3-yl)-2-(spiro[2.3]hexan-5-yl)acetamide	1.75
7GCZ	LWO	2-(1H-benzotriazol-1-yl)-N-[4-(methylamino)phenyl]-N-[(thiophen-3-yl)methyl]acetamide	1.512
7GD0	M4L	(2S)-2-(3-bromophenyl)-2-hydroxy-N-(4-methoxypyridin-3-yl)acetamide	1.649
7GD1	M2X	(2R)-2-amino-2-(5-bromo-2-methoxyphenyl)-N-(4-methylpyridin-3-yl)acetamide	1.52
7GD2	M26	N-[(1R)-1-(3-bromophenyl)-2-methoxyethyl]-2-[(3S)-5-fluoro-2-oxo-2,3-dihydro-1H-indol-3-yl]acetamide	1.65
7GD3	Y6J	~{N}-[4-[2-(benzotriazol-1-yl)ethanoyl-(thiophen-3-ylmethyl)amino]phenyl]cyclopropanecarboxamide	1.751
7GD4	R30	N-{4-[(1H-benzotriazol-1-ylacetyl)(thiophen-3-ylmethyl)amino]phenyl}propanamide	1.749
7GD5	M0X	2-(1H-benzotriazol-1-yl)-N-[4-(dimethylamino)phenyl]-N-[(thiophen-3-yl)methyl]acetamide	1.809
7GD6	M0G	(2S)-2-(3-chlorophenyl)-2-hydroxy-N-(4-methylpyridin-3-yl)butanamide	1.551
7GD7	LZX	(2R)-4-[(methanesulfonyl)amino]-2-phenyl-N-(pyridin-3-yl)butanamide	1.509
7GD8	KU6	(4R)-6-chloro-N-(4-cyclopropyl-4H-1,2,4-triazol-3-yl)-3,4-dihydro-2H-1-benzopyran-4-carboxamide	1.64
7GDA	MF0	(2R)-2-(5-chloropyridin-3-yl)-N-(4-methylpyridin-3-yl)propanamide	1.709
7GDB	M9U	(4S)-6-chloro-N-(4-cyclopropyl-4H-1,2,4-triazol-3-yl)-3,4-dihydro-2H-1-benzopyran-4-carboxamide	1.701
7GDC	M93	(4R)-6-chloro-N-[4-(hydroxymethyl)pyridin-3-yl]-3,4-dihydro-2H-1-benzopyran-4-carboxamide	1.72
7GDD	860	2-(3-chlorophenyl)-N-(isoquinolin-4-yl)acetamide	1.34
7GDE	M7X	2-(3-chlorophenyl)-N-(5-methylpyridin-3-yl)acetamide	1.501
7GDF	M6U	N-(4-ethylpyridin-3-yl)-2-[6-(trifluoromethyl)pyridin-2-yl]acetamide	1.959
7GDG	M6I	N-(3-chlorophenyl)-N'-(pyridin-3-yl)urea	1.811
7GDI	M5I	N-(3-chlorophenyl)-2-(4-methylpyridin-3-yl)acetamide	1.8
7GDJ	M50	2-(3-chlorophenyl)-N-(5-methylpyridazin-4-yl)acetamide	1.769
7GDK	MJR	2-(3-chlorophenyl)-N-(3-methyl-1H-pyrazol-4-yl)acetamide	1.735
7GDL	MKI	2-(3-chlorophenyl)-N-methyl-N-(4-methylpyridin-3-yl)acetamide	1.864
7GDM	MQ3	(2R)-2-cyclohexyl-N-(4-methylpyridin-3-yl)propanamide	1.52
7GDN	MU3	(4R)-N-(4-cyclopropyl-4H-1,2,4-triazol-3-yl)-3,4-dihydro-2H-1-benzopyran-4-carboxamide	1.351
7GDO	MVR	N-[(1-methyl-1H-pyrazol-3-yl)methyl]-2-(pyridin-3-yl)-N-[4-(pyridin-2-yl)phenyl]acetamide	1.756
7GDP	MVX	(3S)-5-chloro-N-(4-cyclopropyl-4H-1,2,4-triazol-3-yl)-2,3-dihydro-1-benzofuran-3-carboxamide	1.821
7GDU	N0F	(3R)-3-(4-hydroxypiperidin-1-yl)-N-(4-methylpyridin-3-yl)-3-(thiophen-3-yl)propanamide	1.63
7GDV	N0O	(3S)-5-chloro-N-(4-phenyl-4H-1,2,4-triazol-3-yl)-2,3-dihydro-1-benzofuran-3-carboxamide	1.907
7GDW	N0X	(4R)-N-(4-cyclopropyl-4H-1,2,4-triazol-3-yl)-4-methyl-3,4-dihydro-2H-1-benzopyran-4-carboxamide	1.667
7GDZ	N3I	2-(3-chloro-5-methoxyphenyl)-N-(4-methylpyridin-3-yl)acetamide	1.839
7GE0	N3R	5-fluoro-N-[2-(2-methoxyphenoxy)ethyl]-2-oxo-1,2-dihydroquinoline-4-carboxamide	1.701
7GE1	N43	5-methoxy-N-[2-(2-methoxyphenoxy)ethyl]-2-oxo-1,2-dihydroquinoline-4-carboxamide	1.936

7GE2	N4L	N-[2-(2-methoxyphenoxy)ethyl]-5-methyl-2-oxo-1,2-dihydroquinoline-4-carboxamide	1.67
7GE3	N5L	N-(5-amino-4-methylpyridin-3-yl)-2-(3-chlorophenyl)acetamide	1.27
7GE4	N6X	N-(5-amino-4-methylpyridin-3-yl)-2-(3-cyanophenyl)acetamide	1.302
7GE5	N7L	(1R,2R)-2-(fluoromethyl)-N-(4-methylpyridin-3-yl)cyclopropane-1-carboxamide	1.641
7GE6	N8X	2-(3,5-dimethylphenyl)-N-(4-methyl-4H-1,2,4-triazol-3-yl)acetamide	1.62
7GE7	N9I	2-(4-methylpyridin-3-yl)-N-(1,2,3,4-tetrahydroisoquinolin-8-yl)acetamide	1.51
7GE8	NB0	4-[4-(2-fluorophenyl)piperazine-1-carbonyl]quinolin-2(1H)-one	1.799
7GE9	NB6	2-(3-bromophenyl)-N-(4-methylpyridin-3-yl)acetamide	1.49
7GEA	N6F	(2S)-N-(4-acetamidopyridin-3-yl)-2-(3-chlorophenyl)propanamide	1.831
7GEB	NDI	2-(4-acetylpiperazin-1-yl)-N-(4-methylpyridin-3-yl)acetamide	1.391
7GEC	NEL	2-(3-chlorophenyl)-N-(1H-indazol-4-yl)acetamide	1.25
7GED	NGX	(2S)-1-(3-chlorophenyl)-3-(1H-1,2,4-triazol-1-yl)propan-2-ol	1.479
7GEE	NIJ	3-(3-fluorophenyl)-N-(4-methylpyridin-3-yl)propanamide	1.29
7GEF	NJE	N-(4-methylpyridin-3-yl)-2-(piperidin-1-yl)acetamide	1.18
7GEG	NJU	2-(3-chlorophenyl)-N-(5,6,7,8-tetrahydroisoquinolin-4-yl)acetamide	1.729
7GEH	NKU	2-(1H-benzotriazol-1-yl)-N-[(3-chlorophenyl)methyl]-N-methylacetamide	1.23
7GEI	NM0	2-(3-chlorophenyl)-N-[(4R)-imidazo[1,2-a]pyridin-3-yl]acetamide	1.69
7GEJ	NO0	2-(3-ethynylphenyl)-N-(isoquinolin-4-yl)acetamide	1.742
7GEK	NOI	2-(3-chlorophenyl)-N-(1-methyl-1H-imidazol-5-yl)acetamide	1.41
7GEL	NQ3	(1M,3P)-1-(3-chlorophenyl)-3-(4-methylpyridin-3-yl)-1,3-dihydro-2H-imidazol-2-one	1.49
7GEM	NQO	2-(3-chlorophenyl)-N-(4-methylpyridazin-3-yl)acetamide	1.32
7GEN	NRC	methyl (2R)-2-(3-chlorophenyl)-3-[(4-methylpyridin-3-yl)amino]-3-oxopropanoate	1.529
7GEO	NRX	2-(3-chlorophenyl)-N-[(4S)-imidazo[1,5-a]pyridin-1-yl]acetamide	1.499
7GEQ	NSR	(4R)-6-chloro-N-(isoquinolin-4-yl)-3,4-dihydro-2H-1-benzopyran-4-carboxamide	2.057
7GER	NU0	2-(3-chlorophenyl)-N-(2,6-naphthyridin-4-yl)acetamide	1.711
7GES	NUR	2-(3-chlorophenyl)-N-(isoquinolin-4-yl)-N-methylacetamide	1.47
7GET	NV9	2-(3-chlorophenyl)-N-(4-phenylpyridin-3-yl)acetamide	1.919
7GEU	NVO	2-(3-chlorophenyl)-N-(phthalazin-1-yl)acetamide	1.4
7GEV	NW0	3-(3-chlorophenyl)-N-(4-methylpyridin-3-yl)propanamide	1.542
7GEW	NWI	2-(3-chlorophenyl)-N-(1,6-naphthyridin-8-yl)acetamide	1.461
7GEX	NX9	2-(3-chlorophenyl)-N-(1H-pyrazol-4-yl)acetamide	1.989
7GEY	NYR	N-(3-chlorophenyl)-2-(3-methyl-1H-pyrazol-4-yl)acetamide	1.341
7GEZ	NZK	2-(4-acetylpiperazin-1-yl)-N-(isoquinolin-4-yl)acetamide	1.34
7GF0	O0C	2-(3-chlorophenyl)-N-(2,7-naphthyridin-4-yl)acetamide	1.579
7GF1	O0R	2-(3-chlorophenyl)-N-(4-cyclopropylpyridin-3-yl)acetamide	1.881
7GF2	O0X	2-(3-chlorophenyl)-N-(1,7-naphthyridin-5-yl)acetamide	1.38
7GF3	O1I	(2S)-2-(3-chlorophenyl)-3-hydroxy-N-(4-methylpyridin-3-yl)propanamide	1.628
7GF4	O1X	(2S)-2-(difluoromethoxy)-N-(4-methylpyridin-3-yl)propanamide	1.561
7GF5	O2R	2-(2-butoxy-5-chlorophenyl)-N-(4-methylpyridin-3-yl)acetamide	1.37
7GF6	O3I	2-[(1M)-5-chloro-2',3'-difluoro-4'-methyl[1,1'-biphenyl]-3-yl]-N-(4-methylpyridin-3-yl)acetamide	1.45
7GFA	Z26	2-(5-chloro-2-methoxyphenyl)-N-(isoquinolin-4-yl)acetamide	1.971
7GFB	NSR	(4R)-6-chloro-N-(isoquinolin-4-yl)-3,4-dihydro-2H-1-benzopyran-4-carboxamide	1.609

7GFC	O87	4-{4-[3-(trifluoromethyl)phenyl]piperazine-1-carbonyl}quinolin-2(1H)-one	2.23
7GFD	O8L	(2S)-2-(3-chloro-5-{[(2R)-4-oxoazetidin-2-yl]oxy}phenyl)-N-(4-methylpyridin-3-yl)propanamide	1.851
7GFE	O9O	N-{3-chloro-5-[(6-methoxypyridin-2-yl)oxy]phenyl}-2-(isoquinolin-4-yl)acetamide	1.65
7GFF	O9X	(2R)-2-(difluoromethoxy)-N-(4-methylpyridin-3-yl)propanamide	1.745
7GFG	OAO	2-{3-chloro-5-[(3-methyl-1,2,4-oxadiazol-5-yl)methoxy]phenyl}-N-(4-methylpyridin-3-yl)acetamide	1.8
7GFH	OBO	2-(3-chlorophenyl)-N-(1H-imidazo[4,5-c]pyridin-7-yl)acetamide	1.46
7GFI	OCI	2-(3-chloro-5-sulfamamidophenyl)-N-(4-methylpyridin-3-yl)acetamide	1.6
7GFJ	OD7	2-(6-methoxy-1H-benzotriazol-1-yl)-N-[4-(piperidin-4-yl)phenyl]-N-[(pyridin-2-yl)methyl]acetamide	1.59
7GFK	ODX	2-(3-chloro-5-{[(1S,2S)-2-hydroxycyclopentyl]amino}phenyl)-N-(4-methylpyridin-3-yl)acetamide	1.5
7GFL	OE6	N-(1H-benzotriazol-1-yl)-2-(3-chlorophenyl)acetamide	1.771
7GFM	OEO	N-[4-(dimethylamino)phenyl]-2-(isoquinolin-4-yl)-N-[(thiophen-3-yl)methyl]acetamide	1.7
7GFN	OFX	2-(1H-benzotriazol-1-yl)-N-[4-(dimethylamino)phenyl]-N-[(1H-pyrazol-5-yl)methyl]acetamide	1.641
7GFO	OGF	2-(1H-benzotriazol-1-yl)-N-[4-(methylcarbamamido)phenyl]-N-[(thiophen-3-yl)methyl]acetamide	1.5
7GFP	OHC	2-[3-(acetamidomethyl)-5-chlorophenyl]-N-(4-methylpyridin-3-yl)acetamide	1.769
7GFQ	OI4	(4R)-6-chloro-N-(4-methylpyridin-3-yl)-3,4-dihydro-2H-1-benzopyran-4-carboxamide	1.551
7GFR	OIE	2-(4-chloropyridin-2-yl)-N-(isoquinolin-4-yl)acetamide	1.46
7GFS	OIK	(isoquinolin-4-yl)(4-phenylpiperazin-1-yl)methanone	1.679
7GFT	OIX	N-[2-(2-methoxyphenoxy)ethyl]isoquinoline-4-carboxamide	1.639
7GFU	OJ9	2-(5-chloro-1-benzofuran-7-yl)-N-(isoquinolin-4-yl)acetamide	1.6
7GFX	OKW	2-(3-chlorophenyl)-N-(6,7-dihydro-5H-cyclopenta[c]pyridin-4-yl)acetamide	1.55
7GFZ	ONU	4-[3-(2-methoxyanilino)azetidine-1-carbonyl]quinolin-2(1H)-one	1.43
7GG0	OGV	2-(3-chlorophenyl)-N-[(4S)-[1,2,4]triazolo[4,3-a]pyridin-3-yl]acetamide	1.512
7GG1	OGO	4-[3-(2-methoxyphenoxy)azetidine-1-carbonyl]quinolin-2(1H)-one	1.481
7GG2	OO6	(3S,4R)-6-chloro-N-(isoquinolin-4-yl)-3-methyl-3,4-dihydro-2H-1-benzopyran-4-carboxamide	1.721
7GG4	OPU	(4R)-6-chloro-N-[(4S)-7-methyl[1,2,4]triazolo[4,3-a]pyridin-8-yl]-3,4-dihydro-2H-1-benzopyran-4-carboxamide	1.572
7GG5	OQF	2-(1H-benzotriazol-1-yl)-N-[4-(dimethylamino)phenyl]-N-[(1,3-thiazol-4-yl)methyl]acetamide	1.509
7GG6	OQL	(4R)-6-chloro-N-(isoquinolin-4-yl)-1,2,3,4-tetrahydroquinoline-4-carboxamide	1.599
7GG7	OQX	2-(1H-benzotriazol-1-yl)-N-[(3-chlorophenyl)methyl]-N-(4-methoxyphenyl)acetamide	1.51
7GG8	ORR	1-(5-amino-3,4-dihydro-1,7-naphthyridin-1(2H)-yl)-2-(3-chlorophenyl)ethan-1-one	1.58
7GG9	SQ2	2-(1H-1,2,3-benzotriazol-1-yl)-1-(4-methylpiperidin-1-yl)ethan-1-one	1.54
7GGA	OSI	(4R)-6-chloro-N-(isoquinolin-4-yl)-4-methyl-3,4-dihydro-2H-1-benzopyran-4-carboxamide	1.49

7GGB	OT6	2-{3-chloro-5-[(2-cyano-2-methylpropyl)amino]phenyl}-N-(4-methylpyridin-3-yl)acetamide	1.381
7GGC	OTV	2-(3-chloro-5-{[(1S,2R)-2-(trifluoromethyl)cyclopropyl]amino}phenyl)-N-(4-methylpyridin-3-yl)acetamide	1.936
7GGD	OUF	N-[(furan-2-yl)methyl]-N'-(2-methyl-1-oxo-1,2-dihydroisoquinolin-4-yl)-N-{3-[(propan-2-yl)oxy]propyl}urea	1.48
7GGE	OV4	2-(1H-benzotriazol-1-yl)-N-benzyl-N-[4-(dimethylamino)phenyl]acetamide	1.63
7GGF	OVF	2-(1H-benzotriazol-1-yl)-N-[(3-chlorophenyl)methyl]-N-[4-(dimethylamino)phenyl]acetamide	1.831
7GGG	OVX	1-(3-chlorophenyl)-4-(isoquinoline-4-carbonyl)piperazin-2-one	1.74
7GGH	OWC	(3S)-3-(3-chlorophenyl)-1-(isoquinolin-4-yl)piperidin-2-one	1.675
7GGI	OWX	2-(3-chlorophenyl)-N-(1,2,3,4-tetrahydro-1,7-naphthyridin-5-yl)acetamide	1.65
7GGJ	OYF	(4R)-6-chloro-N-(2,7-naphthyridin-4-yl)-3,4-dihydro-2H-1-benzopyran-4-carboxamide	1.801
7GGK	OYX	N-(2-cyclohexylethyl)-2-(isoquinolin-4-yl)-N-[(thiophen-2-yl)methyl]acetamide	1.73
7GGL	OZC	(3S)-3-(4-chlorophenyl)-1-(isoquinolin-4-yl)piperidin-2-one	2.218
7GGM	OZX	2-(6-chloropyridin-2-yl)-N-(isoquinolin-4-yl)acetamide	1.839
7GGN	P0X	4-[4-(3-chlorophenyl)-3-oxopiperazine-1-carbonyl]quinolin-2(1H)-one	1.928
7GGO	P3L	(4R)-6-chloro-N-[(4R)-2-oxopiperidin-4-yl]-3,4-dihydro-2H-1-benzopyran-4-carboxamide	1.687
7GGP	P4R	(1'M,4S)-6-chloro-1'-(isoquinolin-4-yl)-2,3-dihydrospiro[[1]benzopyran-4,4'-imidazolidine]-2',5'-dione	1.902
7GGQ	OQL	(4R)-6-chloro-N-(isoquinolin-4-yl)-1,2,3,4-tetrahydroquinoline-4-carboxamide	1.811
7GGR	P6O	(4S)-6-chloro-4-hydroxy-N-(isoquinolin-4-yl)-2-oxo-1,2,3,4-tetrahydroquinoline-4-carboxamide	1.87
7GGS	P7R	(4R)-6-chloro-N-(1-methyl-2-oxo-2,3-dihydro-1H-imidazo[4,5-c]pyridin-7-yl)-3,4-dihydro-2H-1-benzopyran-4-carboxamide	1.49
7GGT	P9O	(4R)-6-chloro-N-(5,6,7,8-tetrahydro-2,6-naphthyridin-4-yl)-3,4-dihydro-2H-1-benzopyran-4-carboxamide	1.82
7GGU	PJ6	(4S)-6-chloro-N-(isoquinolin-4-yl)-4-methoxy-3,4-dihydro-2H-1-benzopyran-4-carboxamide	2.29
7GGV	PJX	(4R)-6-chloro-N-(isoquinolin-4-yl)-1-methyl-1,2,3,4-tetrahydroquinoline-4-carboxamide	1.97
7GGW	PKW	(4R)-6-chloro-N-(1-methyl-1H-imidazo[4,5-c]pyridin-7-yl)-3,4-dihydro-2H-1-benzopyran-4-carboxamide	1.92
7GGX	PQ6	(4R)-4-(aminomethyl)-6-chloro-N-(isoquinolin-4-yl)-3,4-dihydro-2H-1-benzopyran-4-carboxamide	1.85
7GGY	PUU	(4R)-1-acetyl-6-chloro-N-(isoquinolin-4-yl)-1,2,3,4-tetrahydroquinoline-4-carboxamide	2.17
7GGZ	PVR	2-(4-acetylpiperazin-1-yl)-N-(4-cyclopropylpyridin-3-yl)acetamide	1.46
7GH0	PWR	(4R)-6-chloro-N-(2-oxo-2lambda~5~-isoquinolin-4-yl)-3,4-dihydro-2H-1-benzopyran-4-carboxamide	1.69
7GH1	PZ6	(4S)-4-(aminomethyl)-6-chloro-N-(isoquinolin-4-yl)-3,4-dihydro-2H-1-benzopyran-4-carboxamide	1.52
7GH3	Q1C	2-(4-methylpiperidin-1-yl)-N-(4-methylpyridin-3-yl)acetamide	1.901
7GH4	Q1U	2-(3-chlorophenyl)-N-(6-methoxyisoquinolin-4-yl)acetamide	2.27
7GH5	Q2G	N-(3-chlorophenyl)-N'-(4-methylpyridin-3-yl)urea	1.66

7GH6	Q2U	N-[2-(2-methoxyphenoxy)ethyl]-2-oxo-1,2-dihydroquinoline-4-carboxamide	1.57
7GH7	Q36	(2R)-2-(3-cyanophenyl)-N-(4-methylpyridin-3-yl)propanamide	1.67
7GH8	Q3U	N-(5-aminopyridin-3-yl)-N'-(3-chlorophenyl)urea	1.52
7GH9	Q45	2-[(2S)-2-{2-[(methanesulfonyl)amino]ethyl}piperidin-1-yl]-N-(pyridin-3-yl)acetamide	1.57
7GHB	Q4R	N~2~-methyl-N-(4-methylpyridin-3-yl)-N~2~-(quinoline-8-sulfonyl)glycinamide	1.56
7GHC	Q5C	N-(4-methylpyridin-3-yl)-N~2~-[(pyridin-3-yl)acetyl]glycinamide	1.48
7GHD	Q5K	(2S)-N-{2-[(4-fluorobenzene-1-sulfonyl)amino]phenyl}-2-hydroxy-2-(pyridin-3-yl)acetamide	1.97
7GHE	Q5R	4-(4-phenylpiperazine-1-carbonyl)quinolin-2(1H)-one	2.168
7GHF	Q60	N'-[(1-methyl-1H-1,2,3-triazol-4-yl)methyl]-N-(2-phenylethyl)-N-[(pyridin-3-yl)methyl]urea	1.66
7GHK	Q8I	1-(5-fluoro-1H-indol-3-yl)-N-methylmethanamine	1.52
7GHL	Q8O	2-(1H-benzimidazol-6-yl)-N-(4-methylpyridin-3-yl)acetamide	1.41
7GHM	Q99	N-[2-(3-chloro-5-{[(2S)-4-oxoazetidin-2-yl]oxy}phenoxy)ethyl]-2-oxo-1,2-dihydroquinoline-4-carboxamide	1.69
7GHN	QBR	N-(4-tert-butylphenyl)-N-[(1R)-2-{[2-(3-fluorophenyl)ethyl]amino}-2-oxo-1-(pyridin-3-yl)ethyl]furan-2-carboxamide	1.73
7GHO	QC3	(3S)-3-(3-chlorophenyl)-1-(isoquinolin-4-yl)pyrrolidin-2-one	1.861
7GHP	OYF	(4R)-6-chloro-N-(2,7-naphthyridin-4-yl)-3,4-dihydro-2H-1-benzopyran-4-carboxamide	1.54
7GHQ	OYX	N-(2-cyclohexylethyl)-2-(isoquinolin-4-yl)-N-[(thiophen-2-yl)methyl]acetamide	1.72
7GHR	OZC	(3S)-3-(4-chlorophenyl)-1-(isoquinolin-4-yl)piperidin-2-one	1.655
7GHS	OVX	1-(3-chlorophenyl)-4-(isoquinoline-4-carbonyl)piperazin-2-one	1.659
7GHT	OWC	(3S)-3-(3-chlorophenyl)-1-(isoquinolin-4-yl)piperidin-2-one	1.79
7GHU	P4R	(1'M,4S)-6-chloro-1'-(isoquinolin-4-yl)-2,3-dihydrospiro[[1]benzopyran-4,4'-imidazolidine]-2',5'-dione	1.638
7GHV	OZX	2-(6-chloropyridin-2-yl)-N-(isoquinolin-4-yl)acetamide	1.63
7GHW	P3L	(4R)-6-chloro-N-[(4R)-2-oxopiperidin-4-yl]-3,4-dihydro-2H-1-benzopyran-4-carboxamide	1.58
7GHX	P7R	(4R)-6-chloro-N-(1-methyl-2-oxo-2,3-dihydro-1H-imidazo[4,5-c]pyridin-7-yl)-3,4-dihydro-2H-1-benzopyran-4-carboxamide	1.49
7GHY	P9O	(4R)-6-chloro-N-(5,6,7,8-tetrahydro-2,6-naphthyridin-4-yl)-3,4-dihydro-2H-1-benzopyran-4-carboxamide	1.62
7GHZ	PJX	(4R)-6-chloro-N-(isoquinolin-4-yl)-1-methyl-1,2,3,4-tetrahydroquinoline-4-carboxamide	1.666
7GI0	OQL	(4R)-6-chloro-N-(isoquinolin-4-yl)-1,2,3,4-tetrahydroquinoline-4-carboxamide	1.58
7GI1	PJ6	(4S)-6-chloro-N-(isoquinolin-4-yl)-4-methoxy-3,4-dihydro-2H-1-benzopyran-4-carboxamide	1.625
7GI2	PQ6	(4R)-4-(aminomethyl)-6-chloro-N-(isoquinolin-4-yl)-3,4-dihydro-2H-1-benzopyran-4-carboxamide	1.61
7GI4	QCC	2-(3-chloro-5-{[(2S)-4-oxoazetidin-2-yl]oxy}phenyl)-N-(4-methylpyridin-3-yl)acetamide	1.72
7GI5	QCO	N-[(1R)-2-{[2-(3-fluorophenyl)ethyl]amino}-2-oxo-1-(pyridin-3-yl)ethyl]-N-{4-[(propan-2-yl)oxy]phenyl}-1H-imidazole-4-carboxamide	2
7GI6	QD4	(4R)-6-chloro-N~4~-(isoquinolin-4-yl)-3,4-dihydroquinoline-1,4(2H)-dicarboxamide	1.694
7GI7	QD9	N-[(3-chlorophenyl)methyl]-N-[5-(dimethylamino)pyridin-2-yl]-2-(isoquinolin-4-yl)acetamide	1.74

7GI8	QDF	(4R)-6-chloro-N-(isoquinolin-4-yl)-2-oxo-1,2,3,4-tetrahydroquinoline-4-carboxamide	1.8
7GI9	QDU	2-(2,5-difluorophenyl)-N-(isoquinolin-4-yl)acetamide	1.91
7GIA	QE3	2-(5-chloropyridin-2-yl)-N-(isoquinolin-4-yl)acetamide	2.04
7GIB	QER	2-(3-fluorophenyl)-N-(isoquinolin-4-yl)acetamide	1.68
7GIC	QEX	N-(isoquinolin-4-yl)-2-(3-methylphenyl)acetamide	1.92
7GID	QF5	N-(isoquinolin-4-yl)-2-phenylacetamide	1.9
7GIE	QF9	2-(3-chloro-5-cyanophenyl)-N-(isoquinolin-4-yl)acetamide	2.17
7GIF	QFL	2-(4-fluorophenyl)-N-(isoquinolin-4-yl)acetamide	1.68
7GIG	QFU	2-(3-cyanophenyl)-N-(isoquinolin-4-yl)acetamide	1.94
7GIH	QG3	2-(3,5-difluorophenyl)-N-(isoquinolin-4-yl)acetamide	1.63
7GII	QGC	(4R)-6-chloro-N-(isoquinolin-4-yl)-1-[(4H-1,2,4-triazol-3-yl)methyl]-1,2,3,4-tetrahydroquinoline-4-carboxamide	2.12
7GIJ	QGO	(4S)-6-chloro-4-[2-(dimethylamino)-2-oxoethyl]-N-(isoquinolin-4-yl)-3,4-dihydro-2H-1-benzopyran-4-carboxamide	1.9
7GIK	QGX	(2R)-2-amino-2-(3,4-dichlorophenyl)-N-(isoquinolin-4-yl)acetamide	2.09
7GIL	QH6	2-(3-chloro-4-fluorophenyl)-N-(isoquinolin-4-yl)acetamide	2.07
7GIM	QHI	(4R)-6,8-dichloro-N-(isoquinolin-4-yl)-3,4-dihydro-2H-1-benzopyran-4-carboxamide	2.03
7GIN	QHU	(4R)-6,7-dichloro-N-(isoquinolin-4-yl)-3,4-dihydro-2H-1-benzopyran-4-carboxamide	1.858
7GIO	QI4	(2S)-2-(3,4-dichlorophenyl)-N-(isoquinolin-4-yl)propanamide	2.035
7GIP	QI7	(4R)-6-chloro-1-[(1H-imidazol-2-yl)methyl]-N-(isoquinolin-4-yl)-1,2,3,4-tetrahydroquinoline-4-carboxamide	2.05
7GIQ	QIM	(4R)-6-chloro-N-[6-(methanesulfonyl)isoquinolin-4-yl]-3,4-dihydro-2H-1-benzopyran-4-carboxamide	1.916
7GIR	QIB	(4S)-6-chloro-4-hydroxy-N-(isoquinolin-4-yl)-3,4-dihydro-2H-1-benzopyran-4-carboxamide	2.18
7GIS	QIQ	2-(5-chloropyridin-3-yl)-N-(isoquinolin-4-yl)acetamide	1.838
7GIT	QIT	(2S)-2-(3,4-dichlorophenyl)-N-(isoquinolin-4-yl)-2-(methylamino)acetamide	2.06
7GIU	QIZ	(4R)-6-chloro-7-fluoro-N-(isoquinolin-4-yl)-3,4-dihydro-2H-1-benzopyran-4-carboxamide	2
7GIV	QJ6	(2R)-2-(3,4-dichlorophenyl)-N-(isoquinolin-4-yl)-2-methoxyacetamide	2.198
7GIW	QJF	(4R)-6-chloro-N-(6-methoxyisoquinolin-4-yl)-3,4-dihydro-2H-1-benzopyran-4-carboxamide	2.45
7GIX	P4R	(1'M,4S)-6-chloro-1'-(isoquinolin-4-yl)-2,3-dihydrospiro[[1]benzopyran-4,4'-imidazolidine]-2',5'-dione	2.07
7GIY	OI4	(4R)-6-chloro-N-(4-methylpyridin-3-yl)-3,4-dihydro-2H-1-benzopyran-4-carboxamide	2.04
7GIZ	PUU	(4R)-1-acetyl-6-chloro-N-(isoquinolin-4-yl)-1,2,3,4-tetrahydroquinoline-4-carboxamide	1.97
7GJ0	QJL	(4R)-6-chloro-4-{[(N,N-dimethylglycyl)amino]methyl}-N-(isoquinolin-4-yl)-3,4-dihydro-2H-1-benzopyran-4-carboxamide	1.81
7GJ1	QJR	2-(3,4-dichlorophenyl)-N-(2,7-naphthyridin-4-yl)acetamide	1.95
7GJ2	QK3	(4R)-6-chloro-4-{[2-(1H-imidazol-1-yl)acetamido]methyl}-N-(isoquinolin-4-yl)-3,4-dihydro-2H-1-benzopyran-4-carboxamide	1.87
7GJ3	PJ6	(4S)-6-chloro-N-(isoquinolin-4-yl)-4-methoxy-3,4-dihydro-2H-1-benzopyran-4-carboxamide	1.924
7GJ4	QKB	(4R)-6-chloro-N-(6-fluoroisoquinolin-4-yl)-3,4-dihydro-2H-1-benzopyran-4-carboxamide	2.128
7GJ5	PJX	(4R)-6-chloro-N-(isoquinolin-4-yl)-1-methyl-1,2,3,4-tetrahydroquinoline-4-carboxamide	2.13

7GJ6	QKI	(2S)-2-(3,4-dichlorophenyl)-2-hydroxy-N-(isoquinolin-4-yl)acetamide	2.03
7GJ8	QKR	2-(3,4-dichlorophenyl)-2,2-difluoro-N-(isoquinolin-4-yl)acetamide	2.003
7GJ9	QL3	N-(6-acetamidopyridin-3-yl)-N-[(3-chlorophenyl)methyl]-2-(isoquinolin-4-yl)acetamide	2.09
7GJA	QLC	(2R)-2-(3,4-dichlorophenyl)-N-(isoquinolin-4-yl)-2-(2-methoxyethoxy)acetamide	2.097
7GJB	QLO	(4S)-6-chloro-N-(isoquinolin-4-yl)-4-[2-(methylamino)-2-oxoethyl]-3,4-dihydro-2H-1-benzopyran-4-carboxamide	2.03
7GJC	QM3	(1'P,3'S)-6-chloro-1'-(isoquinolin-4-yl)-2,3-dihydrospiro[[1]benzopyran-4,3'-pyrrolidine]-2',5'-dione	1.932
7GJD	QM9	(4S)-4-amino-6-chloro-N-(isoquinolin-4-yl)-3,4-dihydro-2H-1-benzopyran-4-carboxamide	1.79
7GJF	QMX	(4R)-6-chloro-N-(isoquinolin-4-yl)-4-({[(1-methyl-1H-pyrazol-3-yl)methyl]amino}methyl)-3,4-dihydro-2H-1-benzopyran-4-carboxamide	1.96
7GJG	QN9	(4S)-6-chloro-4-{2-[4-(3-hydroxypropyl)piperazin-1-yl]-2-oxoethyl}-N-(isoquinolin-4-yl)-3,4-dihydro-2H-1-benzopyran-4-carboxamide	1.99
7GJH	QNU	(4S)-6-chloro-N-(isoquinolin-4-yl)-1,2,3,4-tetrahydroisoquinoline-4-carboxamide	1.92
7GJI	QO0	(4S)-6,7-dichloro-N-(isoquinolin-4-yl)-4-methoxy-3,4-dihydro-2H-1-benzopyran-4-carboxamide	1.59
7GJJ	QOO	2-{3-chloro-5-[4-(ethanesulfonyl)piperazin-1-yl]phenyl}-N-(isoquinolin-4-yl)acetamide	1.75
7GJK	QOU	(4S)-6-chloro-N-(isoquinolin-4-yl)-4-(2-methoxyethyl)-1,2,3,4-tetrahydroquinoline-4-carboxamide	2.1
7GJL	QOC	(3R)-3-(3,4-dichlorophenyl)-1-(isoquinolin-4-yl)piperidin-2-one	2.17
7GJM	QP0	(3R,4R)-6-chloro-N-(isoquinolin-4-yl)-3-methyl-3,4-dihydro-2H-1-benzopyran-4-carboxamide	1.92
7GJN	QP6	(4S)-6,8-difluoro-N-(isoquinolin-4-yl)-4-methoxy-3,4-dihydro-2H-1-benzopyran-4-carboxamide	2.06
7GJO	QIZ	(4R)-6-chloro-7-fluoro-N-(isoquinolin-4-yl)-3,4-dihydro-2H-1-benzopyran-4-carboxamide	2.2
7GJP	QPQ	(4S)-6-chloro-7-fluoro-N-(isoquinolin-4-yl)-4-methoxy-3,4-dihydro-2H-1-benzopyran-4-carboxamide	1.967
7GJQ	QQ6	(4R)-6-chloro-N-(isoquinolin-4-yl)-3,4-dihydro-2H-1-benzothiopyran-4-carboxamide	1.97
7GJR	QQF	2-(3,4-dichlorophenyl)-N-(isoquinolin-4-yl)-N~2~-(methoxyacetyl)-L-alaninamide	2.25
7GJS	QQO	(2R)-N-{(2R)-2-(3,4-dichlorophenyl)-1-[(isoquinolin-4-yl)amino]-1-oxopropan-2-yl}-4-(propan-2-yl)morpholine-2-carboxamide	2
7GJT	QQU	(4S)-6-chloro-N-(isoquinolin-4-yl)-4-{2-oxo-2-[(2R)-2-(1H-pyrazol-4-yl)piperidin-1-yl]ethyl}-3,4-dihydro-2H-1-benzopyran-4-carboxamide	2.12
7GJU	QR5	(2R)-2-[2-(3-cyclopropyl-2-oxoimidazolidin-1-yl)acetamido]-2-(3,4-dichlorophenyl)-N-(isoquinolin-4-yl)propanamide	1.94
7GJV	QP6	(4S)-6,8-difluoro-N-(isoquinolin-4-yl)-4-methoxy-3,4-dihydro-2H-1-benzopyran-4-carboxamide	2.09
7GJW	QR9	(4S)-4-{2-[(1R,4R)-5-acetyl-2,5-diazabicyclo[2.2.1]heptan-2-yl]-2-oxoethyl}-6-chloro-N-(isoquinolin-4-yl)-3,4-dihydro-2H-1-benzopyran-4-carboxamide	2.456
7GJX	QNU	(4S)-6-chloro-N-(isoquinolin-4-yl)-1,2,3,4-tetrahydroisoquinoline-4-carboxamide	2.06

7GJY	QRS	(3S)-N-{(2R)-2-(3,4-dichlorophenyl)-1-[(isoquinolin-4-yl)amino]-1-oxopropan-2-yl}-1-methylpyrrolidine-3-carboxamide	2.177
7GJZ	QRF	2-{3-chloro-5-[4-(furan-2-carbonyl)piperazin-1-yl]phenyl}-N-(isoquinolin-4-yl)acetamide	1.65
7GK0	QS3	(1'M,4S)-6-chloro-1'-(isoquinolin-4-yl)-3'-methyl-2,3-dihydrospiro[[1]benzopyran-4,4'-imidazolidine]-2',5'-dione	1.96
7GK1	QSF	(3S)-5-chloro-N-(isoquinolin-4-yl)-3-methyl-2-oxo-2,3-dihydro-1H-indole-3-carboxamide	1.937
7GK2	QSX	(4S)-6-chloro-N-(isoquinolin-4-yl)-4-{2-[(1S,4S)-5-methyl-2,5-diazabicyclo[2.2.1]heptan-2-yl]-2-oxoethyl}-3,4-dihydro-2H-1-benzopyran-4-carboxamide	2.01
7GK3	QT3	(1R)-7-chloro-N-(isoquinolin-4-yl)-2-methyl-1,2,3,4-tetrahydroisoquinoline-1-carboxamide	2.19
7GK4	QTC	2-(3-chloro-5-ethylphenyl)-N-(isoquinolin-4-yl)acetamide	2.23
7GK5	QC3	(3S)-3-(3-chlorophenyl)-1-(isoquinolin-4-yl)pyrrolidin-2-one	1.97
7GK6	QTL	(1R)-7-chloro-N-(isoquinolin-4-yl)-1,2,3,4-tetrahydroisoquinoline-1-carboxamide	1.98
7GK7	QU9	(4S)-6-chloro-4-(2-{(2R)-2-[(1H-imidazol-1-yl)methyl]pyrrolidin-1-yl}-2-oxoethyl)-N-(isoquinolin-4-yl)-3,4-dihydro-2H-1-benzopyran-4-carboxamide	2.18
7GK8	QUQ	(4S)-2-acetyl-6-chloro-N-(isoquinolin-4-yl)-1,2,3,4-tetrahydroisoquinoline-4-carboxamide	2.2
7GK9	QV0	(3'R)-6-chloro-1'-(isoquinolin-4-yl)-2,3-dihydrospiro[[1]benzopyran-4,3'-piperidin]-2'-one	2.02
7GKA	QV9	(3R)-3-(3-chlorophenyl)-1-(isoquinolin-4-yl)pyrrolidin-2-one	2.06
7GKB	QVG	(4R)-6-chloro-7-fluoro-N-(6-fluoroisoquinolin-4-yl)-3,4-dihydro-2H-1-benzopyran-4-carboxamide	1.96
7GKC	QVJ	(4R)-6-chloro-N-(isoquinolin-4-yl)-4-{[2-(methylamino)-2-oxoethoxy]methyl}-3,4-dihydro-2H-1-benzopyran-4-carboxamide	1.94
7GKD	QVU	(4R)-6,7-dichloro-N-(2,7-naphthyridin-4-yl)-1,2,3,4-tetrahydroquinoline-4-carboxamide	2.243
7GKE	QW1	(4S)-6-chloro-N~4~-(isoquinolin-4-yl)-3,4-dihydroisoquinoline-2,4(1H)-dicarboxamide	1.97
7GKF	QWL	(4R)-6,7-dichloro-N-(6-fluoroisoquinolin-4-yl)-1,2,3,4-tetrahydroquinoline-4-carboxamide	1.85
7GKG	QWU	(3S)-5-chloro-N-(isoquinolin-4-yl)-3-methyl-2,3-dihydro-1H-indole-3-carboxamide	2.03
7GKH	QX3	(4R)-6,7-dichloro-N-(4-cyclopropylpyridin-3-yl)-1,2,3,4-tetrahydroquinoline-4-carboxamide	1.99
7GKI	QX9	2-(3-chlorophenyl)-N-(6-methylisoquinolin-4-yl)acetamide	2
7GKJ	QXI	(4S)-6-chloro-N-(isoquinolin-4-yl)-4-{2-[(4R,8S)-8-methyl-5,6-dihydro[1,2,4]triazolo[4,3-a]pyrazin-7(8H)-yl]-2-oxoethyl}-3,4-dihydro-2H-1-benzopyran-4-carboxamide	1.76
7GKK	QXR	N-(4-tert-butoxypyridin-3-yl)-2-(3-chlorophenyl)acetamide	1.8
7GKL	QXX	2-(3-chlorophenyl)-N-[4-(trifluoromethyl)pyridin-3-yl]acetamide	1.93
7GKM	QY6	(3R)-6'-chloro-1-(isoquinolin-4-yl)-2',3'-dihydro-1'H-spiro[piperidine-3,4'-quinolin]-2-one	1.95
7GKN	QYI	(4S)-6-chloro-N-(6-fluoroisoquinolin-4-yl)-4-methoxy-3,4-dihydro-2H-1-benzopyran-4-carboxamide	1.92
7GKO	QYN	(4S)-6-chloro-2-(1H-imidazole-2-sulfonyl)-N-(isoquinolin-4-yl)-1,2,3,4-tetrahydroisoquinoline-4-carboxamide	2.05
7GKP	QYR	(4S)-6-chloro-2-(cyclopropanesulfonyl)-N-(isoquinolin-4-yl)-1,2,3,4-tetrahydroisoquinoline-4-carboxamide	1.794
7GKQ	QZ0	(4S)-6-chloro-N~4~-(isoquinolin-4-yl)-N~2~,N~2~-dimethyl-3,4-dihydroisoquinoline-2,4(1H)-dicarboxamide	2.07

7GKR	QZC	(4S)-6-chloro-2-(dimethylsulfamoyl)-N-(isoquinolin-4-yl)-1,2,3,4-tetrahydroisoquinoline-4-carboxamide	2.02
7GKS	QZL	(4S)-6-chloro-N-(isoquinolin-4-yl)-2-[(3R)-3-methylpyrrolidine-1-sulfonyl]-1,2,3,4-tetrahydroisoquinoline-4-carboxamide	1.809
7GKT	QZU	(4R)-6-chloro-N-(isoquinolin-4-yl)-4-[(prop-2-enamido)methyl]-3,4-dihydro-2H-1-benzopyran-4-carboxamide	1.839
7GKU	R08	(4S)-6-chloro-N-(isoquinolin-4-yl)-2-(1-methyl-1H-pyrazole-5-carbonyl)-1,2,3,4-tetrahydroisoquinoline-4-carboxamide	1.866
7GKV	R0F	(4S)-6-chloro-N-(isoquinolin-4-yl)-2-[2-(methylamino)-2-oxoethyl]-1,2,3,4-tetrahydroisoquinoline-4-carboxamide	1.876
7GKW	R0Q	methyl ({(4R)-6-chloro-4-[(isoquinolin-4-yl)carbamoyl]-3,4-dihydro-2H-1-benzopyran-4-yl}methyl)carbamate	1.911
7GKX	R1I	(4S)-6-chloro-N-(isoquinolin-4-yl)-2-(2-methoxyethyl)-1,2,3,4-tetrahydroisoquinoline-4-carboxamide	1.866
7GKY	R1U	2-(3-chlorophenyl)-N-(1-methyl-1H-pyrazolo[4,3-c]pyridin-7-yl)acetamide	1.969
7GKZ	R2L	(4R)-6-chloro-N-[4-methyl-5-(methylamino)pyridin-3-yl]-3,4-dihydro-2H-1-benzopyran-4-carboxamide	1.808
7GL0	R2X	(2S,5R)-N-{(1R)-1-(3-chlorophenyl)-2-[(isoquinolin-4-yl)amino]-2-oxoethyl}-5-(pyrrolidine-1-carbonyl)oxolane-2-carboxamide (non-preferred name)	1.821
7GL1	R3I	(3S)-5-chloro-1'-(6-fluoroisoquinolin-4-yl)-2H-spiro[[1]benzofuran-3,3'-pyrrolidin]-2'-one	1.709
7GL2	R43	(4S)-6-chloro-2-(3-cyanoazetidine-1-sulfonyl)-N-(isoquinolin-4-yl)-1,2,3,4-tetrahydroisoquinoline-4-carboxamide	1.808
7GL3	R4X	(4S)-2-(azetidine-1-sulfonyl)-6-chloro-N-(isoquinolin-4-yl)-1,2,3,4-tetrahydroisoquinoline-4-carboxamide	1.751
7GL4	R5H	(4S)-6-chloro-N-(isoquinolin-4-yl)-2-(3-methoxyazetidine-1-sulfonyl)-1,2,3,4-tetrahydroisoquinoline-4-carboxamide	1.868
7GL5	R5O	(4S)-6-chloro-N-{6-[(methanesulfonyl)amino]isoquinolin-4-yl}-4-methoxy-3,4-dihydro-2H-1-benzopyran-4-carboxamide	1.678
7GL6	R66	(3R)-3-(3-chlorophenyl)-3-hydroxy-1-(isoquinolin-4-yl)pyrrolidin-2-one	1.721
7GL7	R6L	(4S)-6-chloro-4-methoxy-N-[7-(methylsulfamoyl)isoquinolin-4-yl]-3,4-dihydro-2H-1-benzopyran-4-carboxamide	1.851
7GL8	KG9	(4S)-6-chloro-2-[(1-cyanocyclopropyl)methanesulfonyl]-N-(isoquinolin-4-yl)-1,2,3,4-tetrahydroisoquinoline-4-carboxamide	1.637
7GL9	QM3	(1'P,3'S)-6-chloro-1'-(isoquinolin-4-yl)-2,3-dihydrospiro[[1]benzopyran-4,3'-pyrrolidine]-2',5'-dione	1.944
7GLA	R7F	(4R)-6-chloro-N-[6-(2-hydroxypropan-2-yl)isoquinolin-4-yl]-1,2,3,4-tetrahydroquinoline-4-carboxamide	1.684
7GLB	R76	(4S)-6-chloro-N-(isoquinolin-4-yl)-2-[2-(methylamino)-2-oxoethyl]-1-oxo-1,2,3,4-tetrahydroisoquinoline-4-carboxamide	1.95
7GLC	R87	(4S)-6-chloro-N-(7-fluoroisoquinolin-4-yl)-4-methoxy-3,4-dihydro-2H-1-benzopyran-4-carboxamide	1.954
7GLD	R8I	(4S)-6-chloro-2-{2-[(cyanomethyl)amino]-2-oxoethyl}-N-(isoquinolin-4-yl)-1,2,3,4-tetrahydroisoquinoline-4-carboxamide	1.78
7GLE	R8O	(4S)-6-chloro-2-[2-(cyclopropylamino)-2-oxoethyl]-N-(isoquinolin-4-yl)-1,2,3,4-tetrahydroisoquinoline-4-carboxamide	1.984
7GLF	R8X	(4S)-6-chloro-N-(isoquinolin-4-yl)-2-[(2-methoxyethyl)(methyl)sulfamoyl]-1,2,3,4-tetrahydroisoquinoline-4-carboxamide	1.67
7GLG	R95	(4S)-6-chloro-2-[(cyanomethyl)(methyl)sulfamoyl]-N-(isoquinolin-4-yl)-1,2,3,4-tetrahydroisoquinoline-4-carboxamide	1.673

7GLH	R9E	2-(3-chlorophenyl)-N-(7-fluoro-6-methoxyisoquinolin-4-yl)acetamide	1.906
7GLI	R9I	methyl N-[(4S)-6-chloro-4-[(isoquinolin-4-yl)carbamoyl]-3,4-dihydroisoquinoline-2(1H)-sulfonyl]-N-methylglycinate	1.839
7GLJ	R9R	(4S)-6-chloro-N-(isoquinolin-4-yl)-2-[(3-methyl-1,1-dioxo-1lambda~6~-thietan-3-yl)methanesulfonyl]-1,2,3,4-tetrahydroisoquinoline-4-carboxamide	1.814
7GLK	R9Z	(4S)-6-chloro-N-(isoquinolin-4-yl)-2-[(1-methoxycyclopropyl)methanesulfonyl]-1,2,3,4-tetrahydroisoquinoline-4-carboxamide	1.744
7GLL	RAQ	(4S)-6-chloro-N-(isoquinolin-4-yl)-2-[(oxan-4-yl)methanesulfonyl]-1,2,3,4-tetrahydroisoquinoline-4-carboxamide	1.905
7GLM	RBM	(4S)-6-chloro-2-[(2-cyanoethyl)(methyl)sulfamoyl]-N-(isoquinolin-4-yl)-1,2,3,4-tetrahydroisoquinoline-4-carboxamide	1.906
7GLN	RBX	(4S)-6-chloro-2-[ethyl(methyl)sulfamoyl]-N-(isoquinolin-4-yl)-1,2,3,4-tetrahydroisoquinoline-4-carboxamide	1.836
7GLO	L6D	N-(1H-benzimidazol-1-yl)-2-(3-chlorophenyl)acetamide	1.927
7GLP	860	2-(3-chlorophenyl)-N-(isoquinolin-4-yl)acetamide	1.917
7GLQ	NM0	2-(3-chlorophenyl)-N-[(4R)-imidazo[1,2-a]pyridin-3-yl]acetamide	2.063
7GLR	O0X	2-(3-chlorophenyl)-N-(1,7-naphthyridin-5-yl)acetamide	1.788
7GLS	OE6	N-(1H-benzotriazol-1-yl)-2-(3-chlorophenyl)acetamide	1.731
7GLT	RC9	(4S)-6-chloro-2-[(1-cyanocyclobutyl)methanesulfonyl]-N-(isoquinolin-4-yl)-1,2,3,4-tetrahydroisoquinoline-4-carboxamide	1.863
7GLU	RD5	2-(5-chloro-2-{[(methanesulfonyl)amino]methyl}phenyl)-N-(isoquinolin-4-yl)acetamide	1.627
7GLV	R76	(4S)-6-chloro-N-(isoquinolin-4-yl)-2-[2-(methylamino)-2-oxoethyl]-1-oxo-1,2,3,4-tetrahydroisoquinoline-4-carboxamide	1.716
7GLW	RDK	(4S)-6-chloro-N-(isoquinolin-4-yl)-4-methyl-1,1-dioxo-1,2,3,4-tetrahydro-1lambda~6~,2-benzothiazine-4-carboxamide	1.587
7GLX	RDQ	(4S)-6-chloro-N-{6-[(methanesulfonyl)amino]isoquinolin-4-yl}-2-[2-(methylamino)-2-oxoethyl]-1-oxo-1,2,3,4-tetrahydroisoquinoline-4-carboxamide	1.494
7GLY	RDX	(4S)-6-chloro-2-[(2-hydroxyethyl)(methyl)sulfamoyl]-N-(isoquinolin-4-yl)-1,2,3,4-tetrahydroisoquinoline-4-carboxamide	1.805
7GLZ	REU	(4S)-6-chloro-N-(isoquinolin-4-yl)-2-[(2S)-1-(methylamino)-1-oxopropan-2-yl]-1,2,3,4-tetrahydroisoquinoline-4-carboxamide	1.678
7GM0	RFF	(4S)-6-chloro-2-[(1-cyanocyclopropyl)methanesulfonyl]-N-(1-methyl-1H-pyrazolo[4,3-c]pyridin-7-yl)-1,2,3,4-tetrahydroisoquinoline-4-carboxamide	1.76
7GM1	RFR	(4S)-6-chloro-N-{6-[(methanesulfonyl)amino]isoquinolin-4-yl}-2-[2-(methylamino)-2-oxoethyl]-1,2,3,4-tetrahydroisoquinoline-4-carboxamide	1.939
7GM2	RG3	(4S)-6-chloro-N-[7-(methanesulfonyl)isoquinolin-4-yl]-2-[2-(methylamino)-2-oxoethyl]-1-oxo-1,2,3,4-tetrahydroisoquinoline-4-carboxamide	1.891
7GM3	RG9	2-(3-chlorophenyl)-N-[7-(2-hydroxypropan-2-yl)isoquinolin-4-yl]acetamide	2.02
7GM4	RGQ	(4S)-6-chloro-N-(7-fluoroisoquinolin-4-yl)-2-[2-(methylamino)-2-oxoethyl]-1,2,3,4-tetrahydroisoquinoline-4-carboxamide	1.751
7GM5	RGX	(4S)-6-chloro-2-(cyclopropylsulfamoyl)-N-(isoquinolin-4-yl)-1,2,3,4-tetrahydroisoquinoline-4-carboxamide	1.709
7GM6	RHI	(4S)-6-chloro-N-[7-(methanesulfonyl)isoquinolin-4-yl]-2-[2-(methylamino)-2-oxoethyl]-1,2,3,4-tetrahydroisoquinoline-4-carboxamide	1.87

7GM7	RI1	(4S)-6-chloro-N-(isoquinolin-4-yl)-1,1-dioxo-1,2,3,4-tetrahydro-1lambda~6~,2-benzothiazine-4-carboxamide	1.844
7GM8	RI6	(4S)-6-chloro-N-(7-chloroisoquinolin-4-yl)-2-[2-(methylamino)-2-oxoethyl]-1,2,3,4-tetrahydroisoquinoline-4-carboxamide	1.73
7GM9	RIJ	2-[(1'P,3'S)-6-chloro-1'-(isoquinolin-4-yl)-2',5'-dioxo-1H-spiro[isoquinoline-4,3'-pyrrolidin]-2(3H)-yl]-N-methylacetamide	1.826
7GMA	R2X	(2S,5R)-N-{(1R)-1-(3-chlorophenyl)-2-[(isoquinolin-4-yl)amino]-2-oxoethyl}-5-(pyrrolidine-1-carbonyl)oxolane-2-carboxamide (non-preferred name)	1.865
7GMB	RIU	2-(3-chlorophenyl)-N-{6-[2-(dimethylamino)ethoxy]isoquinolin-4-yl}acetamide	2.207
7GMC	RIY	(4S)-6-chloro-2-(ethylsulfamoyl)-N-(isoquinolin-4-yl)-1,2,3,4-tetrahydroisoquinoline-4-carboxamide	1.819
7GMD	R2X	(2S,5R)-N-{(1R)-1-(3-chlorophenyl)-2-[(isoquinolin-4-yl)amino]-2-oxoethyl}-5-(pyrrolidine-1-carbonyl)oxolane-2-carboxamide (non-preferred name)	1.937
7GME	RJ3	4-[2-(3-chlorophenyl)acetamido]isoquinoline-7-carboxylic acid	1.846
7GMF	RJF	(4S)-6-chloro-2-[2-(methylamino)-2-oxoethyl]-N-(5-methylisoquinolin-4-yl)-1-oxo-1,2,3,4-tetrahydroisoquinoline-4-carboxamide	1.805
7GMG	RJO	(4S)-6-chloro-N-(6-fluoroisoquinolin-4-yl)-2-[2-(methylamino)-2-oxoethyl]-1-oxo-1,2,3,4-tetrahydroisoquinoline-4-carboxamide	1.81
7GMH	RJX	4-[2-(3-chlorophenyl)acetamido]isoquinoline-6-carboxylic acid	1.848
7GMI	RK6	4-[2-(3-chlorophenyl)acetamido]-N-methylisoquinoline-7-carboxamide	2.01
7GMJ	RKC	(3'R)-6-chloro-1'-(isoquinolin-4-yl)-2,3-dihydrospiro[[1]benzopyran-4,3'-pyrrolidin]-2'-one	1.84
7GMK	RKR	2-(3-chlorophenyl)-N-[6-(dimethylamino)isoquinolin-4-yl]acetamide	1.845
7GML	RL0	2-(3-chlorophenyl)-N-{6-[(methanesulfonyl)(methyl)amino]isoquinolin-4-yl}acetamide	1.912
7GMM	RL8	4-[2-(3-chlorophenyl)acetamido]-N-methylisoquinoline-6-carboxamide	1.842
7GMN	RLH	2-(3-chlorophenyl)-N-{7-[2-(pyrrolidin-1-yl)ethoxy]isoquinolin-4-yl}acetamide	2.195
7GMO	RLR	(4S)-6-chloro-N-(isoquinolin-4-yl)-2-[(3R)-2-oxopyrrolidin-3-yl]-1,2,3,4-tetrahydroisoquinoline-4-carboxamide	1.855
7GMP	RM3	2-(3-chlorophenyl)-N-[7-(dimethylamino)isoquinolin-4-yl]acetamide	2.083
7GMQ	RMI	2-(3-chlorophenyl)-N-{7-[2-(dimethylamino)ethoxy]isoquinolin-4-yl}acetamide	1.859
7GMR	RN0	4-[2-(3-chlorophenyl)acetamido]isoquinolin-6-yl methanesulfonate	1.837
7GMS	RNI	(4S)-6-chloro-2-[(1-cyanocyclopropyl)methanesulfonyl]-N-{7-[(methanesulfonyl)amino]isoquinolin-4-yl}-1,2,3,4-tetrahydroisoquinoline-4-carboxamide	1.897
7GMT	ROZ	4-[2-(3-chlorophenyl)acetamido]isoquinoline-6-carboxamide	1.738
7GMU	RPK	N-(6-acetamidoisoquinolin-4-yl)-2-(3-chlorophenyl)acetamide	1.737
7GMV	RPZ	2-[(3'S)-6-chloro-1'-(isoquinolin-4-yl)-2'-oxo-1H-spiro[isoquinoline-4,3'-pyrrolidin]-2(3H)-yl]-N-methylacetamide	1.899
7GMW	RQ6	(4S)-6-chloro-N-(7-fluoroisoquinolin-4-yl)-2-[2-(methylamino)-2-oxoethyl]-1-oxo-1,2,3,4-tetrahydroisoquinoline-4-carboxamide	1.826
7GMX	RQO	4-[2-(3-chlorophenyl)acetamido]-N,N-dimethylisoquinoline-6-carboxamide	2.03

7GMY	RQF	(4S)-6-chloro-N-(7-fluoroisoquinolin-4-yl)-1-oxo-2-[2-oxo-2-(propylamino)ethyl]-1,2,3,4-tetrahydroisoquinoline-4-carboxamide	1.838
7GMZ	RR0	(2r,4r)-6-chloro-N-(7-fluoroisoquinolin-4-yl)-2,3-dihydro-4H-2,4-methano-1-benzopyran-4-carboxamide	1.617
7GN0	RRD	(4S)-6-chloro-N-(isoquinolin-4-yl)-2-{2-[(oxetan-3-yl)amino]-2-oxoethyl}-1-oxo-1,2,3,4-tetrahydroisoquinoline-4-carboxamide	1.806
7GN1	RRU	1-{[(3'S)-6-chloro-1'-(isoquinolin-4-yl)-2'-oxo-1H-spiro[isoquinoline-4,3'-pyrrolidine]-2(3H)-sulfonyl]methyl}cyclopropane-1-carbonitrile	1.903
7GN2	RS6	(4S)-6-chloro-N-(7-fluoroisoquinolin-4-yl)-1-oxo-2-{2-oxo-2-[(propan-2-yl)amino]ethyl}-1,2,3,4-tetrahydroisoquinoline-4-carboxamide	1.735
7GN3	RSL	4-[2-(3-chlorophenyl)acetamido]isoquinolin-7-yl methanesulfonate	2.012
7GN4	RT4	(4S)-6-chloro-2-[2-(cyclopropylamino)-2-oxoethyl]-N-(isoquinolin-4-yl)-1-oxo-1,2,3,4-tetrahydroisoquinoline-4-carboxamide	1.72
7GN5	RT9	(4S)-6-chloro-N-(isoquinolin-4-yl)-2-[(1-methoxycyclopropyl)methanesulfonyl]-1-oxo-1,2,3,4-tetrahydroisoquinoline-4-carboxamide	1.863
7GN6	RTS	(4S)-6-chloro-N-(isoquinolin-4-yl)-4-methyl-2-[2-(methylamino)-2-oxoethyl]-1,1-dioxo-1,2,3,4-tetrahydro-1lambda~6~,2-benzothiazine-4-carboxamide	2.44
7GN7	RV0	(4S)-6-chloro-4-ethyl-N-(isoquinolin-4-yl)-1,1-dioxo-1,2,3,4-tetrahydro-1lambda~6~,2-benzothiazine-4-carboxamide	2.349
7GN8	RPZ	2-[(3'S)-6-chloro-1'-(isoquinolin-4-yl)-2'-oxo-1H-spiro[isoquinoline-4,3'-pyrrolidin]-2(3H)-yl]-N-methylacetamide	1.903
7GN9	RVL	(4S)-6-chloro-N-(isoquinolin-4-yl)-2-[1-(methylcarbamoyl)cyclopropyl]-1-oxo-1,2,3,4-tetrahydroisoquinoline-4-carboxamide	2.155
7GNA	RVR	(4S)-6-chloro-1,1-dioxo-N-(5,6,7,8-tetrahydroisoquinolin-4-yl)-1,2,3,4-tetrahydro-1lambda~6~,2-benzothiazine-4-carboxamide	2.093
7GNB	RW0	(3R,4S)-6-chloro-N-[7-(methanesulfonyl)isoquinolin-4-yl]-3-methyl-2-[2-(methylamino)-2-oxoethyl]-1-oxo-1,2,3,4-tetrahydroisoquinoline-4-carboxamide	1.671
7GNC	RW9	2-[(3'S)-6-chloro-1'-(isoquinolin-4-yl)-1,2'-dioxo-1H-spiro[isoquinoline-4,3'-pyrrolidin]-2(3H)-yl]-N-methylacetamide	1.683
7GND	RVR	(4S)-6-chloro-1,1-dioxo-N-(5,6,7,8-tetrahydroisoquinolin-4-yl)-1,2,3,4-tetrahydro-1lambda~6~,2-benzothiazine-4-carboxamide	1.655
7GNE	RWO	(4R)-6-chloro-4-methyl-1,1-dioxo-N-(5,6,7,8-tetrahydroisoquinolin-4-yl)-1,2,3,4-tetrahydro-1lambda~6~,2-benzothiazine-4-carboxamide	1.739
7GNF	RWT	(4S)-6-chloro-4-methyl-1,1-dioxo-N-(5,6,7,8-tetrahydroisoquinolin-4-yl)-1,2,3,4-tetrahydro-1lambda~6~,2-benzothiazine-4-carboxamide	1.69
7GNG	RXU	2-[(3'S)-6-chloro-2'-oxo-1'-(5,6,7,8-tetrahydroisoquinolin-4-yl)-1H-spiro[isoquinoline-4,3'-pyrrolidin]-2(3H)-yl]-N-methylacetamide	1.769
7GNH	RYB	1-{[(3'S)-6-chloro-1'-{6-[2-(dimethylamino)ethoxy]isoquinolin-4-yl}-2'-oxo-1H-spiro[isoquinoline-4,3'-pyrrolidine]-2(3H)-sulfonyl]methyl}cyclopropane-1-carbonitrile	1.785
7GNI	RZF	2-[(3'S)-6-chloro-1'-(6-chloroisoquinolin-4-yl)-1,2'-dioxo-1H-spiro[isoquinoline-4,3'-pyrrolidin]-2(3H)-yl]-N-methylacetamide	1.644

7GNJ	RZU	1-[(3'S)-6-chloro-1'-(isoquinolin-4-yl)-1,2'-dioxo-1H-spiro[isoquinoline-4,3'-pyrrolidin]-2(3H)-yl]-N-methylcyclopropane-1-carboxamide	1.45
7GNK	S0X	2-[(3'S)-6-chloro-1'-(isoquinolin-4-yl)-2'-oxo-1H-spiro[isoquinoline-4,3'-pyrrolidin]-2(3H)-yl]-N-(cyclopropylmethyl)acetamide	1.72
7GNL	S1U	(4S)-6-chloro-2-{2-[4-(4-ethylpiperazin-1-yl)anilino]-2-oxoethyl}-N-(isoquinolin-4-yl)-1,2,3,4-tetrahydroisoquinoline-4-carboxamide	1.681
7GNM	S1L	(4S)-6-chloro-N-(isoquinolin-4-yl)-2-{2-[3-(morpholin-4-yl)anilino]-2-oxoethyl}-1,2,3,4-tetrahydroisoquinoline-4-carboxamide	1.75
7GNN	S39	(4S)-2-{2-[(1,3-benzothiazol-5-yl)amino]-2-oxoethyl}-6-chloro-N-(isoquinolin-4-yl)-1,2,3,4-tetrahydroisoquinoline-4-carboxamide	1.81
7GNO	S3X	(4S)-6-chloro-N-(isoquinolin-4-yl)-2-(2-{[(1S)-1-(4-nitrophenyl)ethyl]amino}-2-oxoethyl)-1,2,3,4-tetrahydroisoquinoline-4-carboxamide	1.661
7GNP	S4X	(4S)-6-chloro-2-(2-{[(1r,3R,5R,7S)-3-hydroxyadamantan-1-yl]amino}-2-oxoethyl)-N-(isoquinolin-4-yl)-1,2,3,4-tetrahydroisoquinoline-4-carboxamide	1.55
7GNQ	S5L	(4S)-2-[2-(4-acetamidoanilino)-2-oxoethyl]-6-chloro-N-(isoquinolin-4-yl)-1,2,3,4-tetrahydroisoquinoline-4-carboxamide	1.531
7GNR	RZU	1-[(3'S)-6-chloro-1'-(isoquinolin-4-yl)-1,2'-dioxo-1H-spiro[isoquinoline-4,3'-pyrrolidin]-2(3H)-yl]-N-methylcyclopropane-1-carboxamide	1.819
7GNS	S6K	1-{[(3'S,4'R)-6-chloro-1'-(isoquinolin-4-yl)-4'-methyl-2'-oxo-1H-spiro[isoquinoline-4,3'-pyrrolidine]-2(3H)-sulfonyl]methyl}cyclopropane-1-carbonitrile	1.617
7GNT	S7C	1-{[(3'S,4'R)-6-chloro-4'-ethyl-1'-(isoquinolin-4-yl)-2'-oxo-1H-spiro[isoquinoline-4,3'-pyrrolidine]-2(3H)-sulfonyl]methyl}cyclopropane-1-carbonitrile	1.561
7GNU	S7U	1-{[(3'S)-6-chloro-1'-(isoquinolin-4-yl)-2'-oxo-1H-spiro[isoquinoline-4,3'-piperidine]-2(3H)-sulfonyl]methyl}cyclopropane-1-carbonitrile	1.48
7GRE	XWH	4-[3-(trifluoromethyl)-1H-pyrazol-5-yl]pyridine	1.66
7JU7	G65	Masitinib	1.6
7KX5	X7V	N-([1,1'-biphenyl]-4-yl)-N-[(1R)-2-oxo-2-{[(1S)-1-phenylethyl]amino}-1-(pyridin-3-yl)ethyl]furan-2-carboxamide	2.6
7L0D	0EN	N-[(1R)-2-(tert-butylamino)-2-oxo-1-(pyridin-3-yl)ethyl]-N-(4-tert-butylphenyl)furan-2-carboxamide	2.39
7L10	XEY	2-[3-(3,5-dichlorophenyl)-2-oxo[2H-[1,3'-bipyridine]]-5-yl]benzonitrile	1.63
7L11	XF1	2-[3-(3-chloro-5-propoxyphenyl)-2-oxo[2H-[1,3'-bipyridine]]-5-yl]benzonitrile	1.8
7L12	XF4	(5S)-5-{3-[3-(benzyloxy)-5-chlorophenyl]-2-oxo[2H-[1,3'-bipyridine]]-5-yl}pyrimidine-2,4(3H,5H)-dione	1.8
7L13	XF7	(5S)-5-(3-{3-chloro-5-[(2-chlorophenyl)methoxy]phenyl}-2-oxo[2H-[1,3'-bipyridine]]-5-yl)pyrimidine-2,4(3H,5H)-dione	2.17
7L14	XFD	2-{3-[3-chloro-5-(cyclopropylmethoxy)phenyl]-2-oxo[2H-[1,3'-bipyridine]]-5-yl}benzonitrile	1.8
7L5D	XNJ	N-(4-methyl-3-{[4-(pyridin-3-yl)-1,3-thiazol-2-yl]amino}phenyl)-4-[(piperazin-1-yl)methyl]benzamide	1.58
7LCT	XU4	N-{(2S)-1-hydroxy-3-[(3S)-2-oxopyrrolidin-3-yl]propan-2-yl}-N~2~-{[(1S)-1-phenylethoxy]carbonyl}-L-leucinamide	1.93

7LMD	Y6A	2-(benzotriazol-1-yl)-~{N}-[4-(1~{H}-pyrazol-4-yl)phenyl]-~{N}-(thiophen-3-ylmethyl)ethanamide	1.96
7LME	Y6J	~{N}-[4-[2-(benzotriazol-1-yl)ethanoyl-(thiophen-3-ylmethyl)amino]phenyl]cyclopropanecarboxamide	2.1
7LMF	Y6G	2-(benzotriazol-1-yl)-~{N}-[4-(1~{H}-imidazol-4-yl)phenyl]-~{N}-(thiophen-3-ylmethyl)ethanamide	2.2
7LTJ	YD1	6-[4-(3,4-dichlorophenyl)piperazin-1-yl]carbonyl-1~{H}-pyrimidine-2,4-dione	1.8
7M8M	YSG	5-[3-(3-chloro-5-propoxyphenyl)-2-oxo-2H-[1,3'-bipyridin]-5-yl]pyrimidine-2,4(1H,3H)-dione	1.78
7M8N	YSP	5-(3-{3-chloro-5-[(2-methylphenyl)methoxy]phenyl}-2-oxo-2H-[1,3'-bipyridin]-5-yl)pyrimidine-2,4(1H,3H)-dione	1.96
7M8O	YSM	5-(3-{3-chloro-5-[(3-fluorophenyl)methoxy]phenyl}-2-oxo-2H-[1,3'-bipyridin]-5-yl)pyrimidine-2,4(1H,3H)-dione	2.44
7M8P	YSJ	5-(3-{3-chloro-5-[(2-chlorophenyl)methoxy]-4-fluorophenyl}-2-oxo-2H-[1,3'-bipyridin]-5-yl)pyrimidine-2,4(1H,3H)-dione	2.23
7M8X	YTJ	2-{3-[3-chloro-5-(2-methoxyethoxy)phenyl]-2-oxo-2H-[1,3'-bipyridin]-5-yl}benzonitrile	1.74
7M8Y	YTM	5-{3-[3-chloro-5-(2-phenylethoxy)phenyl]-2-oxo-2H-[1,3'-bipyridin]-5-yl}pyrimidine-2,4(1H,3H)-dione	1.75
7M8Z	YTV	5-{3-[3-chloro-5-(3-hydroxy-3-methylbutoxy)phenyl]-2-oxo-2H-[1,3'-bipyridin]-5-yl}pyrimidine-2,4(1H,3H)-dione	1.79
7M90	YTS	5-(3-{3-chloro-5-[2-(3-oxopiperazin-1-yl)ethoxy]phenyl}-2-oxo-2H-[1,3'-bipyridin]-5-yl)pyrimidine-2,4(1H,3H)-dione	2.19
7M91	YU4	5-{3-[3-chloro-5-(3,3,3-trifluoropropoxy)phenyl]-2-oxo-2H-[1,3'-bipyridin]-5-yl}pyrimidine-2,4(1H,3H)-dione	1.95
7N44	06I	5-(3-{3-chloro-5-[(5-methyl-1,3-thiazol-4-yl)methoxy]phenyl}-2-oxo-2H-[1,3'-bipyridin]-5-yl)pyrimidine-2,4(1H,3H)-dione	1.94
7N8C	YD1	6-[4-(3,4-dichlorophenyl)piperazin-1-yl]carbonyl-1~{H}-pyrimidine-2,4-dione	2.2, 2.5
7NBT	U7W	2-(benzotriazol-1-yl)-1-[(4~{S})-4-methyl-6,7-dihydro-4~{H}-thieno[3,2-c]pyridin-5-yl]ethanone	1.63
7NEO	U9H	2-cyclobutyl-7-(5-fluoropyridin-3-yl)-5,7-diazaspiro[3.4]octane-6,8-dione	1.64
7NT3	UQZ	~{N}-[(1~{S})-2-(1,3-benzodioxol-5-ylmethylamino)-1-(3-hydroxyphenyl)-2-oxidanylidene-ethyl]-~{N}-propyl-prop-2-enamide	2.325
7O46	V18	2-cyclobutyl-7-isoquinolin-4-yl-5,7-diazaspiro[3.4]octane-6,8-dione	2.23
7P2G	4N0	(4~{R})-~{N}-(4-iodophenyl)-2-oxidanylidene-3,4-dihydro-1~{H}-quinoline-4-carboxamide	2.5
7PFM	7IL	N-[(1R)-2-(tert-butylamino)-2-oxidanylidene-1-pyridin-3-yl-ethyl]-N-(4-tert-butylphenyl)-1H-imidazole-5-carboxamide	2
7PHZ	X77	N-(4-tert-butylphenyl)-N-[(1R)-2-(cyclohexylamino)-2-oxo-1-(pyridin-3-yl)ethyl]-1H-imidazole-4-carboxamide	1.66
7QBB	V1B	7-isoquinolin-4-yl-2-phenyl-5,7-diazaspiro[3.4]octane-6,8-dione	2
7QT5	GWS	2-cyclohexyl-~{N}-pyridin-3-yl-ethanamide	2.26
7QT6	RZJ	1-methyl-3,4-dihydro-2~{H}-quinoline-7-sulfonamide	2.11
7RLS	5YN	6-[4-(3,4,5-trichlorophenyl)piperazine-1-carbonyl]pyrimidine-2,4(1H,3H)-dione	2
7RM2	5YJ	6-[4-(3,5-dichloro-4-methylphenyl)piperazine-1-carbonyl]pyrimidine-2,4(1H,3H)-dione	2
7RMB	5Z7	6-[4-(4-bromo-3-chlorophenyl)piperazine-1-carbonyl]pyrimidine-2,4(1H,3H)-dione	2

7RME	5Z3	6-{4-[4-chloro-3-(trifluoromethyl)phenyl]piperazine-1-carbonyl}pyrimidine-2,4(1H,3H)-dione	2
7RMT	5ZN	2-chloro-4-[4-(2,6-dioxo-1,2,5,6-tetrahydropyrimidine-4-carbonyl)piperazin-1-yl]benzaldehyde	2
7RMZ	5ZJ	6-{4-[3-chloro-4-(trifluoromethyl)phenyl]piperazine-1-carbonyl}pyrimidine-2,4(1H,3H)-dione	2.1
7RN4	H69	6-[4-(3,4-dichlorophenyl)piperidine-1-carbonyl]pyrimidine-2,4(1H,3H)-dione	1.85
7RNH	5ZW	6-[4-(4-chlorophenyl)piperazine-1-carbonyl]pyrimidine-2,4(1H,3H)-dione	2
7RNK	5ZT	6-{4-[3-chloro-4-(hydroxymethyl)phenyl]piperazine-1-carbonyl}pyrimidine-2,4(3H,5H)-dione	2.1
7S3K	Z26	2-(5-chloro-2-methoxyphenyl)-N-(isoquinolin-4-yl)acetamide	1.9
7S3S	860	2-(3-chlorophenyl)-N-(isoquinolin-4-yl)acetamide	2
7S4B	87H	(2R)-2-(3-fluorophenyl)-N-(isoquinolin-4-yl)propanamide	2
7TEK	I2D	N-[(3-chlorophenyl)methyl]-N-[4-(1H-pyrazol-4-yl)phenyl]-2-(pyridin-3-yl)acetamide	2.2
7TEL	I2N	N-[(3-chloro-5-fluorophenyl)methyl]-N-[4-(1H-imidazol-4-yl)phenyl]-2-(isoquinolin-4-yl)acetamide	2.4
7URB	O5O	(2P)-2-(isoquinolin-4-yl)-1-[(1s,3R)-3-(methylcarbamoyl)cyclobutyl]-N-{(1S)-1-[4-(trifluoromethyl)phenyl]butyl}-1H-benzimidazole-7-carboxamide	2.14
7US4	O69	(2P)-2-(isoquinolin-4-yl)-1-[(1s,3R)-3-(methylcarbamoyl)cyclobutyl]-N-[(1S)-1-(naphthalen-2-yl)ethyl]-1H-benzimidazole-7-carboxamide	2.07
7V1T	5IL	5,8-bis(oxidanylidene)-7-[(2-piperazin-1-ylphenyl)amino]naphthalene-1-sulfonamide	2.562
7VIC	ODN	(1beta,6beta,7beta,8alpha,9beta,10alpha,13alpha,14R,16beta)-1,6,7,14-tetrahydroxy-7,20-epoxykauran-15-one	2.1
7VTH	7XB	2-[4-[[4-[bis(fluoranyl)methoxy]-2-methyl-phenyl]amino]-2,6-bis(oxidanylidene)-3-[[3,4,5-tris(fluoranyl)phenyl]methyl]-1,3,5-triazin-1-yl]-N-methyl-ethanamide	2
7VU6	7YY	6-[(6-chloranyl-2-methyl-indazol-5-yl)amino]-3-[(1-methyl-1,2,4-triazol-3-yl)methyl]-1-[[2,4,5-tris(fluoranyl)phenyl]methyl]-1,3,5-triazine-2,4-dione	1.8
7WO3	59S	(2S)-2-[[(2S)-2-[[(E)-3-(4-methoxyphenyl)prop-2-enoyl]amino]-3-methyl-butanoyl]amino]-4-methyl-N-[(2S)-1-oxidanylidene-3-[(3S)-2-oxidanylidenepiperidin-3-yl]propan-2-yl]pentanamide	2.01
8A4Q	V9R	~{tert}-butyl ~{N}-[1-[(2~{R})-3-cyclopropyl-1-oxidanylidene-1-[[(2~{S},3~{S})-3-oxidanyl-4-oxidanylidene-1-[(3~{S})-2-oxidanylidenepyrrolidin-3-yl]-4-[(phenylmethyl)amino]butan-2-yl]amino]propan-2-yl]-2-oxidanylidene-pyridin-3-yl]carbamate	1.75
8ACD	LQ6	(2~{S})-4-[[2,4-bis(oxidanylidene)-1~{H}-pyrimidin-6-yl]carbonyl]-1-(3,4-dichlorophenyl)-~{N}-(thiophen-2-ylmethyl)piperazine-2-carboxamide	1.39
8ACL	LQL	(2~{S})-1-(3,4-dichlorophenyl)-4-pyridin-3-ylcarbonyl-~{N}-(thiophen-2-ylmethyl)piperazine-2-carboxamide	1.4
8CYU	P5X	N-[(4-chlorothiophen-2-yl)methyl]-N-[4-(dimethylamino)phenyl]-2-(isoquinolin-4-yl)acetamide	1.8
8CYZ	P6I	N-[(4-chlorothiophen-2-yl)methyl]-2-(isoquinolin-4-yl)-N-[4-(methylsulfanyl)phenyl]acetamide	1.9
8CZ4	P6R	N-(4-tert-butylphenyl)-N-[(4-chlorothiophen-2-yl)methyl]-2-(isoquinolin-4-yl)acetamide	2.1
8CZ7	P7L	N-[(4-chlorothiophen-2-yl)methyl]-2-(isoquinolin-4-yl)-N-(4-methoxyphenyl)acetamide	2

8DIG	U26	(3P)-1-[(4-fluorophenyl)methyl]-3-(isoquinolin-4-yl)imidazolidine-2,4-dione	2.45
8DIH	U2B	(1P,1'R)-1-(isoquinolin-4-yl)-2',3'-dihydrospiro[imidazolidine-4,1'-indene]-2,5-dione	2.12
8DII	U2I	(2S)-N-(isoquinolin-4-yl)-2-methyl-2,3-dihydro-1,4-benzoxazepine-4(5H)-carboxamide	2.59
8DZ0	7YY	6-[(6-chloranyl-2-methyl-indazol-5-yl)amino]-3-[(1-methyl-1,2,4-triazol-3-yl)methyl]-1-[[2,4,5-tris(fluoranyl)phenyl]methyl]-1,3,5-triazine-2,4-dione	2.29
8GQT	QUE	3,5,7,3',4'-PENTAHYDROXYFLAVONE	2.09
8GTV	KAE	4-[(2~{S})-4-(3,4-dichlorophenyl)-2-(morpholin-4-ylmethyl)piperazin-1-yl]carbonyl-1~{H}-quinolin-2-one	1.8
8GTW	K9U	(2S)-4-(3,4-dichlorophenyl)-1-[(2-oxidanylidene-1H-quinolin-4-yl)carbonyl]-N-[3,3,3-tris(fluoranyl)propyl]piperazine-2-carboxamide	1.85
8HBK	7YY	6-[(6-chloranyl-2-methyl-indazol-5-yl)amino]-3-[(1-methyl-1,2,4-triazol-3-yl)methyl]-1-[[2,4,5-tris(fluoranyl)phenyl]methyl]-1,3,5-triazine-2,4-dione	1.8
8HEF	7YY	6-[(6-chloranyl-2-methyl-indazol-5-yl)amino]-3-[(1-methyl-1,2,4-triazol-3-yl)methyl]-1-[[2,4,5-tris(fluoranyl)phenyl]methyl]-1,3,5-triazine-2,4-dione	1.51
8HUR	7YY	6-[(6-chloranyl-2-methyl-indazol-5-yl)amino]-3-[(1-methyl-1,2,4-triazol-3-yl)methyl]-1-[[2,4,5-tris(fluoranyl)phenyl]methyl]-1,3,5-triazine-2,4-dione	1.64
8I4S	OU3	3-(4-fluoranyl-3-methyl-phenyl)-2-(2-methylpropyl)-5,6,7-tris(oxidanyl)quinazolin-4-one	2.2
8PH4	YQN	~{N}-(4-~{tert}-butylphenyl)-~{N}-[(1~{S})-2-(cyclohexylamino)-2-oxidanylidene-1-pyridin-3-yl-ethyl]-2,6-bis(oxidanylidene)-5~{H}-pyrimidine-5-carboxamide	1.69
8Q71	KKO	(2~{S})-1-(3,4-dichlorophenyl)-4-(4-methoxypyridin-3-yl)carbonyl-~{N}-(thiophen-2-ylmethyl)piperazine-2-carboxamide	2.322
8R11	XI0	1-[(2~{S})-2-(3-chlorophenyl)pyrrolidin-1-yl]-2-(5-methylpyridin-3-yl)ethanone	1.31
8R12	XH9	2-[[4-(5-chloranylpyridin-3-yl)carbonyl-1,4-diazepan-1-yl]methyl]benzenecarbonitrile	1.587
8R14	XHW	(5-chloranylpyridin-3-yl)-[4-[(2-chlorophenyl)methyl]-1,4-diazepan-1-yl]methanone	1.336
8R16	XJ9	1-[6,7-bis(chloranyl)-3,4-dihydro-1H-isoquinolin-2-yl]-2-(5-methylpyridin-3-yl)ethanone	1.3
8SXR	WZK	N-[(4-chlorothiophen-2-yl)methyl]-N-[4-(dimethylamino)phenyl]-2-(5-hydroxyisoquinolin-4-yl)acetamide	2.114
8TPG	JKL	(3R)-N-(4-tert-butylphenyl)-N-[(1R)-2-(cyclohexylamino)-2-oxo-1-(pyridin-3-yl)ethyl]-3-hydroxybutanamide	1.692

Table S2. Intermolecular interaction energies between F2F-2020198-00X and its interacting residues from Mpro.

Residue	Interaction Mode	ΔE_{Int}^{gas}(kcal/mol)	ΔE_{Deh}(kcal/mol)	ΔE_{Int}^{aq}(kcal/mol)
His41	CH-π	-1.8	-0.9	-2.7
Ser144	HB	-3.6	+1.5	-2.1
Glu166	HB	-21.7	+15.9	-5.8
Gly143	HB	-3.8	+2.3	-1.5
His163	HB	-12.0	+10.1	-1.9
His164	HB	-7.4	+6.3	-1.1

Table S3. Intermolecular interaction energies between EDG-MED-10fcb19e-1 and its interacting residues from Mpro.

Residue	Interaction Mode	ΔE_{Int}^{gas}(kcal/mol)	ΔE_{Deh}(kcal/mol)	ΔE_{Int}^{aq}(kcal/mol)
His41	π-π	-4.1	+1.7	-2.4
Met49	CH-π	-3.6	+0.2	-3.4
Met165	CH-π	-6.8	+3.4	-3.4
Asn142	CH-π	-5.3	+1.9	-3.4
Ser144	HB	-1.2	+0.7	-0.5
His163	HB	-8.7	+5.9	-2.8
Glu166	HB	-12.3	+9.0	-3.3

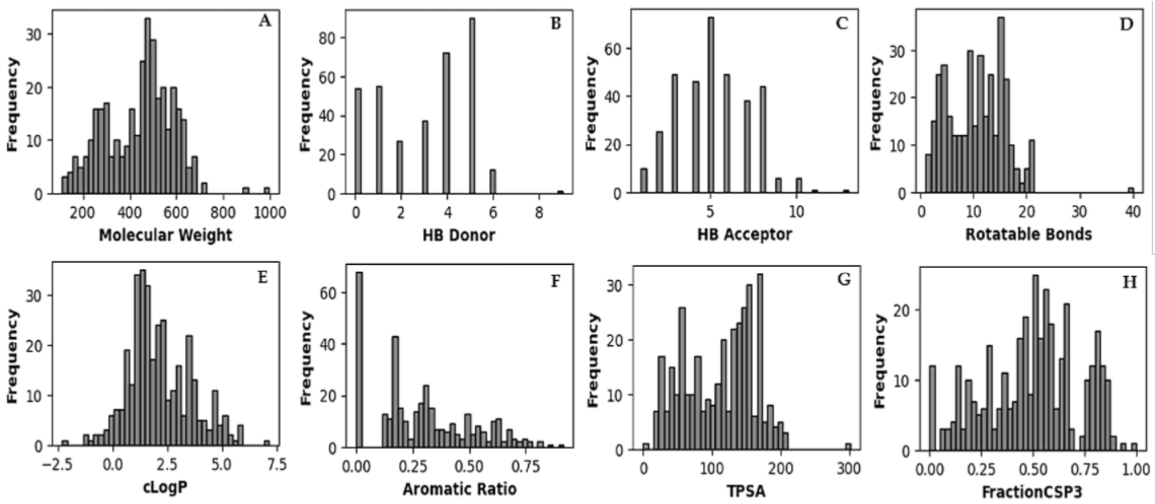

Figure S1. Distribution of molecular descriptors for covalent inhibitors. (A) Molecular weight (in Daltons); (B) number of HB donor atoms; (C) number of HB acceptor atoms; (D) number of rotatable bonds; (E) calculated partition coefficient between octanol and water; (F) aromatic ratio; (G) topological polar surface area (in $Å^2$); and (H) fraction of sp^3 hybridized carbon atoms.

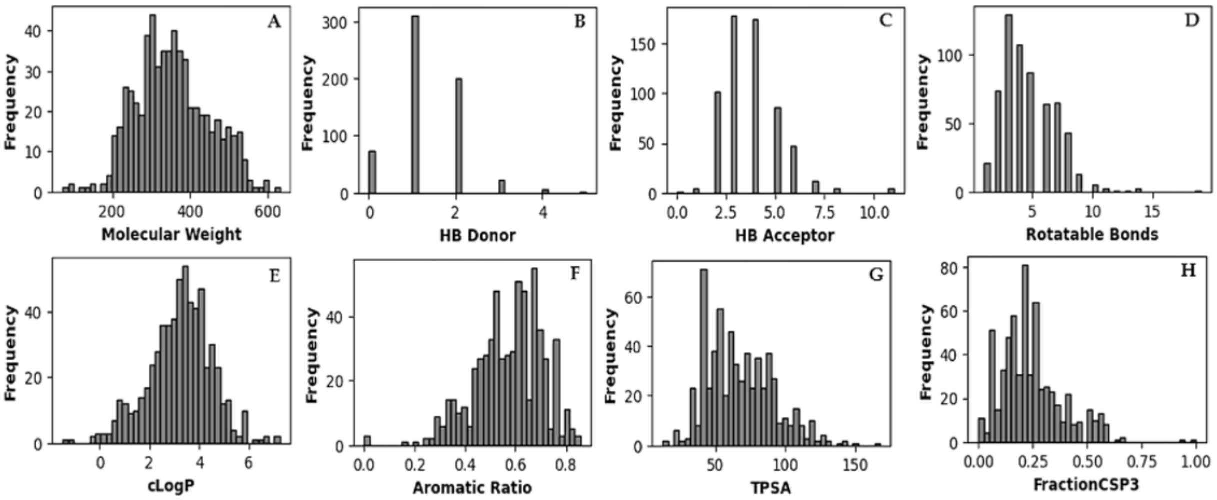

Figure S2. Distribution of molecular descriptors for non-covalent inhibitors. (A) Molecular weight (in Daltons); (B) number of HB donor atoms; (C) number of HB acceptor atoms; (D) number of rotatable bonds; (E) calculated partition coefficient between octanol and water; (F) aromatic ratio; (G) topological polar surface area (in Å^2); and (H) fraction of sp^3 hybridized carbon atoms.

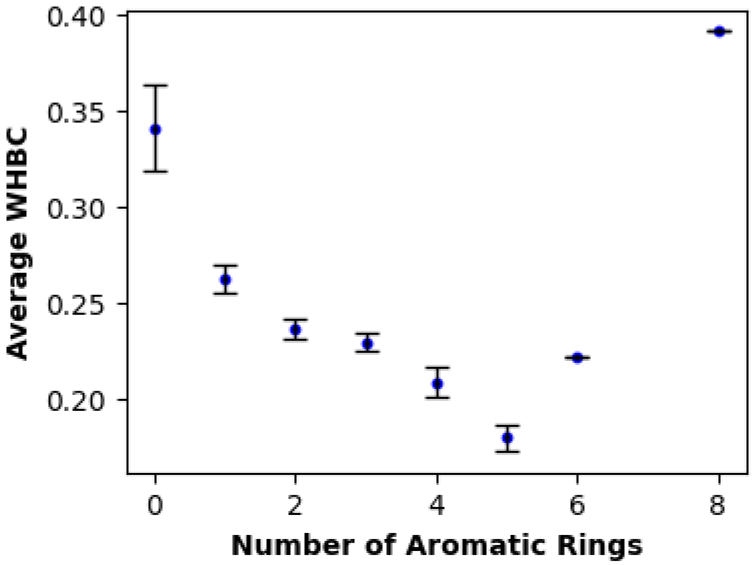

Figure S3. Plot of average of weighted hydrogen bond count versus number of aromatic rings for a dataset of 1000 compounds randomly extracted from the PubChem library (see text).

Validation of WHBC Descriptor A random dataset of 1000 compounds was generated from the PubChem library [59] using a Python script named PubChemPy [60] and subjected to the same cheminformatics analysis previously applied to covalent and non-covalent Mpro inhibitors. The average WHBC values were plotted against the number of aromatic rings to examine potential correlations. The resulting figure indicates that although an inverse correlation between WHBC and aromatic ring count may be apparent for the MPro inhibitors (see Section 2.2.2), it does not hold true in randomly selected compound set. Notably, the observed spike of WHBC at higher aromatic ring counts (e.g., eight rings) suggests that additional molecular features or broader structural diversity likely influence WHBC values beyond the aromatic ring count alone.

Figure S4A.   3D Structures of non-bonded interactions in covalent inhibitors

<table>
<tr><td colspan="2">PDB ID: 5RFJ</td></tr>
<tr><td>HB</td><td>Non-bonded π-interactions</td></tr>
<tr><td>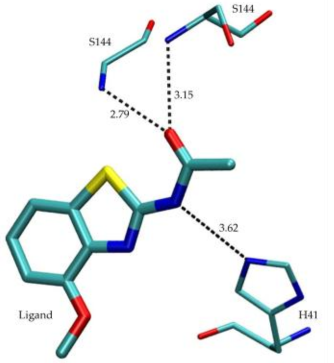
</td><td>None</td></tr>
<tr><td colspan="2">PDB ID: 6XR3</td></tr>
<tr><td>HB</td><td>Non-bonded π-interactions</td></tr>
<tr><td>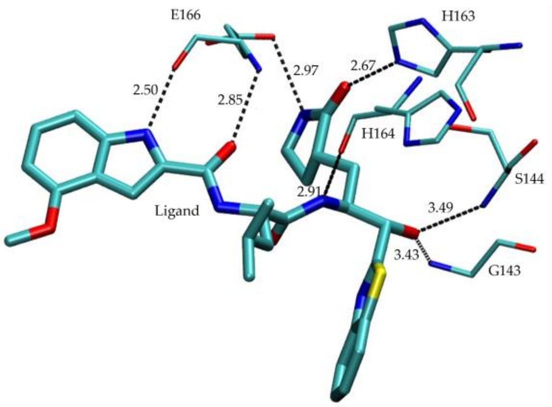
</td><td>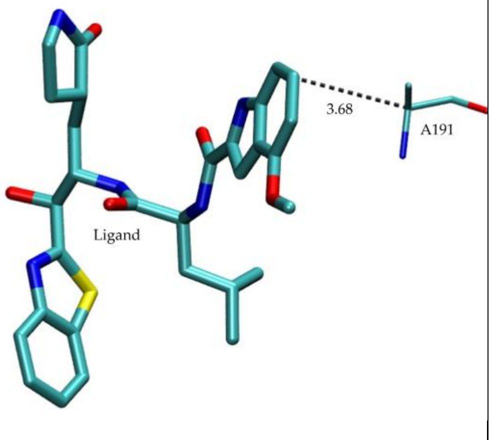
</td></tr>
<tr><td colspan="2">PDB ID: 7SF1</td></tr>
<tr><td>HB</td><td>Non-bonded π-interactions</td></tr>
<tr><td>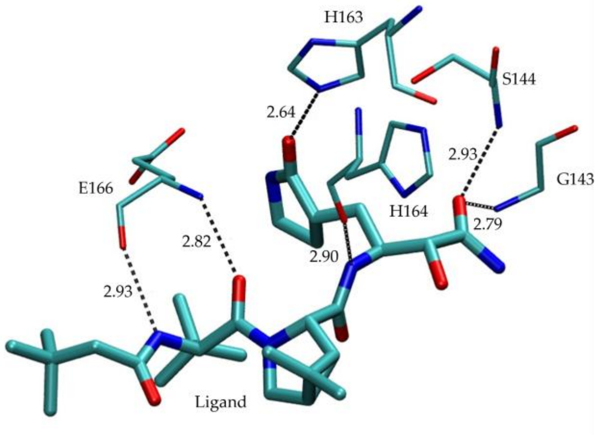
</td><td>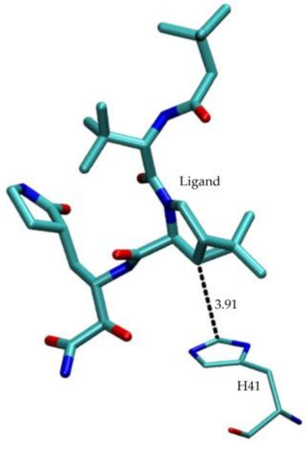
</td></tr>
</table>

<table>
<tr><td colspan="2">PDB ID: 7UUC</td></tr>
<tr><td>HB</td><td>Non-bonded π-interactions</td></tr>
<tr><td>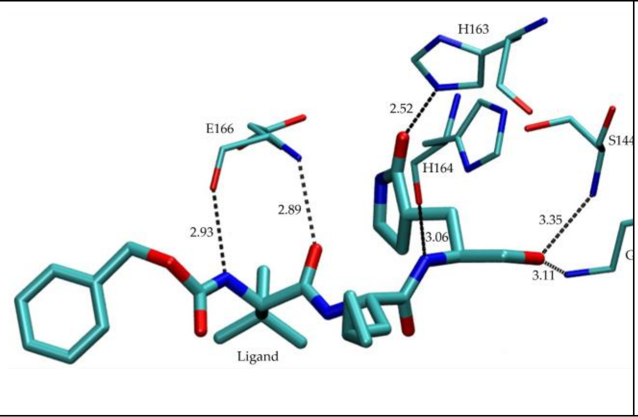
</td><td>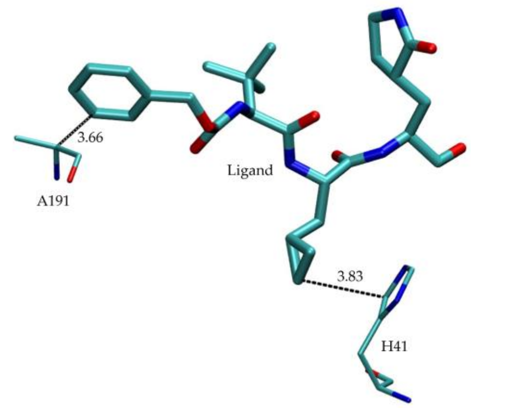
</td></tr>
<tr><td colspan="2">PDB ID: 8GXI</td></tr>
<tr><td>HB</td><td>Non-bonded π-interactions</td></tr>
<tr><td>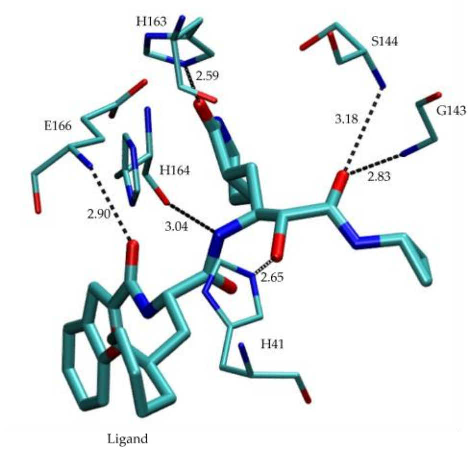
</td><td>None</td></tr>
<tr><td colspan="2">PDB ID: 8OKN</td></tr>
<tr><td>HB</td><td>Non-bonded π-interactions</td></tr>
<tr><td>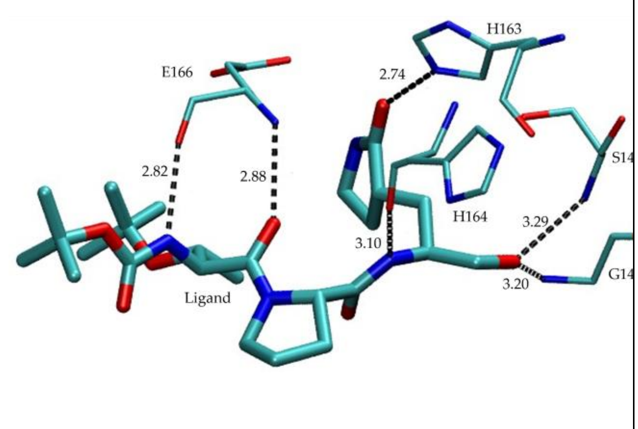
</td><td>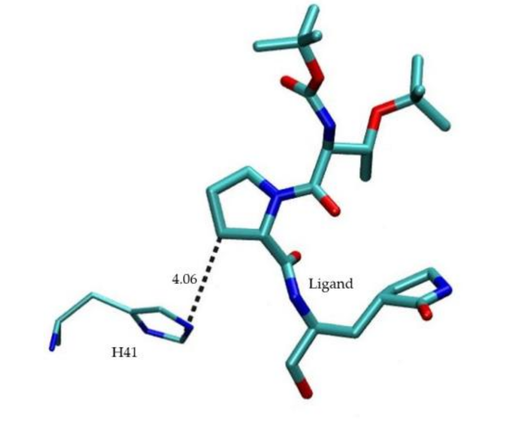
</td></tr>
</table>

Figure S4B. 3D Structures of non-bonded interactions in non-covalent inhibitors

<table>
<tr><td colspan="2">PDB ID: 7ANS</td></tr>
<tr><td>HB</td><td>Non-bonded π-interactions</td></tr>
<tr><td>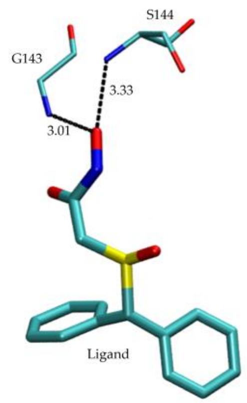</td><td>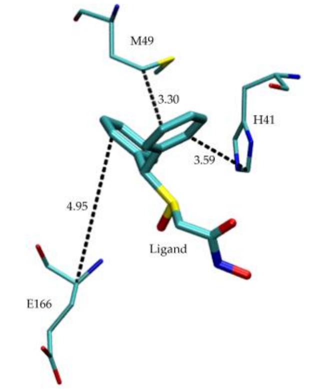</td></tr>
<tr><td colspan="2">PDB ID: 7GBS</td></tr>
<tr><td>HB</td><td>Non-bonded π-interactions</td></tr>
<tr><td>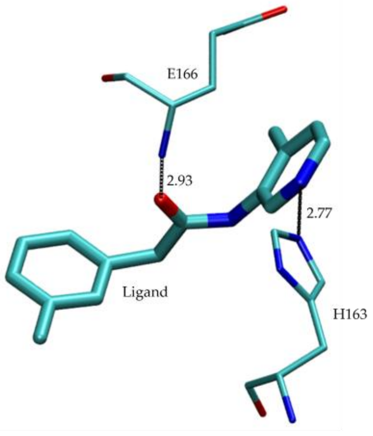</td><td>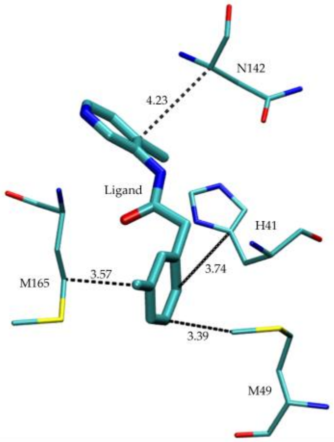</td></tr>
<tr><td colspan="2">PDB ID: 7GDJ</td></tr>
<tr><td>HB</td><td>Non-bonded π-interactions</td></tr>
<tr><td>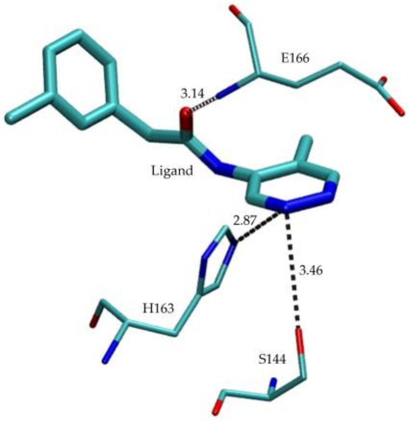</td><td>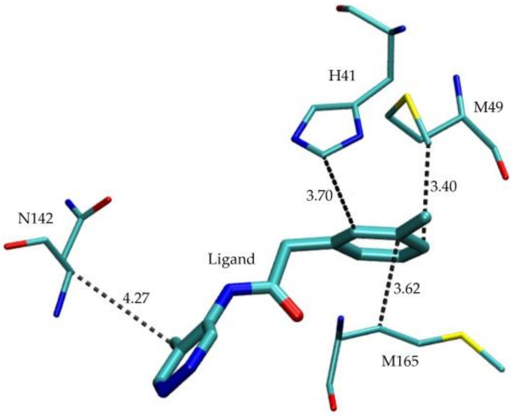</td></tr>
</table>

<table>
<tr><td colspan="2">PDB ID: 7GEM</td></tr>
<tr><td>HB</td><td>Non-bonded π-interactions</td></tr>
<tr><td>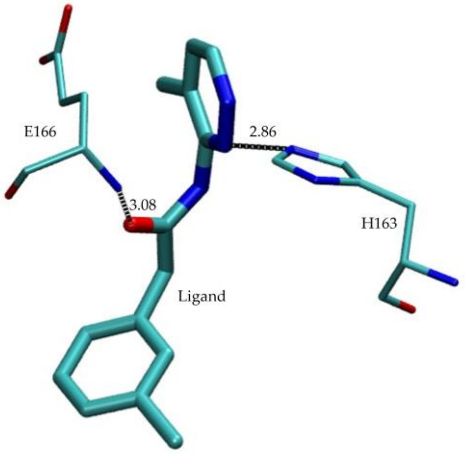
</td><td>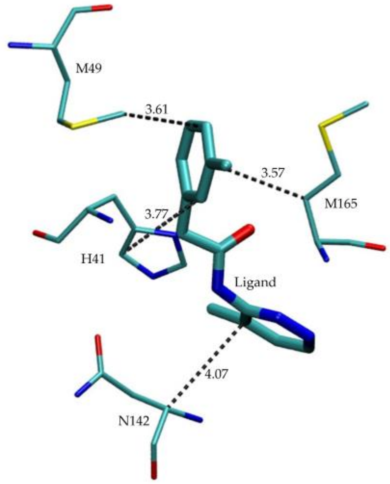
</td></tr>
<tr><td colspan="2">PDB ID: 7GK3</td></tr>
<tr><td>HB</td><td>Non-bonded π-interactions</td></tr>
<tr><td>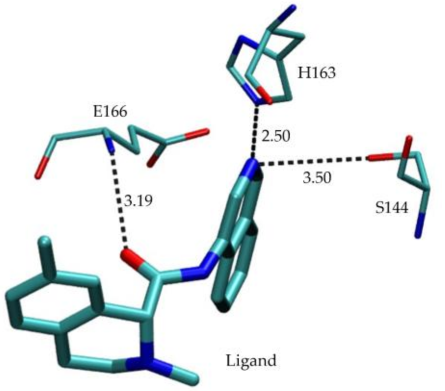
</td><td>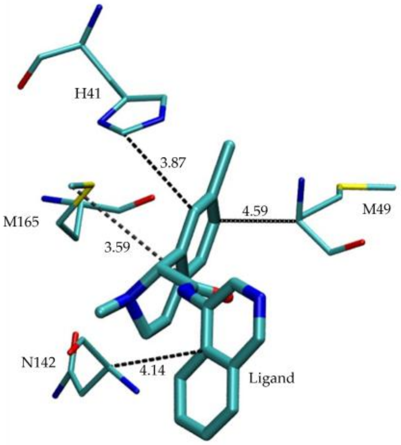
</td></tr>
<tr><td colspan="2">PDB ID: 7GNN</td></tr>
<tr><td>HB</td><td>Non-bonded π-interactions</td></tr>
<tr><td>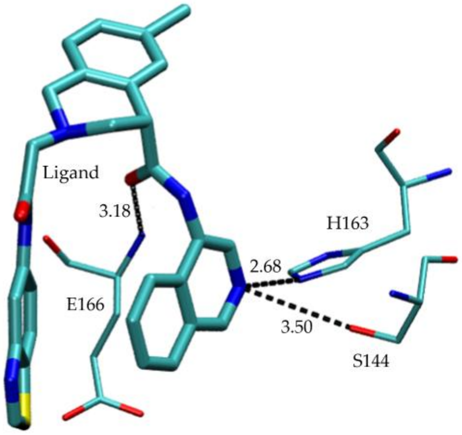
</td><td>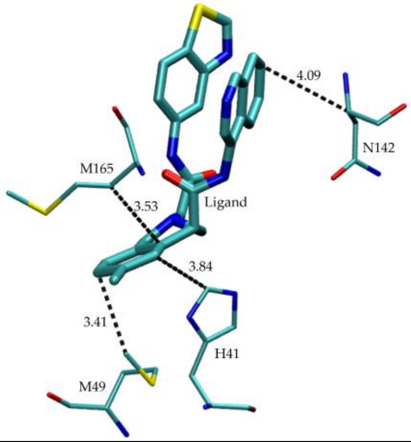
</td></tr>
</table>

Figure S4. Three-dimensional representations of distinct non-bonded interactions for representative covalent and non-covalent inhibitors. Left panels: ligand–protein interactions highlighting residues engaged exclusively in hydrogen bonding interactions. Right panels: ligand–protein interactions illustrating residues participating solely in non-bonded π-interactions.
